# In vitro gut microbiome response to carbohydrate supplementation is acutely affected by a sudden change in diet

**DOI:** 10.1186/s12866-023-02776-2

**Published:** 2023-01-28

**Authors:** Ida Gisela Pantoja-Feliciano, J. Philip Karl, Matthew Perisin, Laurel A. Doherty, Holly L. McClung, Nicholes J. Armstrong, Rebecca Renberg, Kenneth Racicot, Tobyn Branck, Steve Arcidiacono, Jason W. Soares

**Affiliations:** 1 Soldier Effectiveness Directorate (SED), U.S. Army DEVCOM Soldier Center, Natick, MA USA; 2 Military Nutrition Division, U.S. Army Research Institute of Environmental Medicine (USARIEM), Natick, MA USA; 3 U.S. Army DEVCOM Army Research Laboratory, Adelphi, MD USA; 4 General Technical Services, U.S. Army DEVCOM Army Research Laboratory, Adelphi, MD USA

**Keywords:** Gut microbiome, Carbohydrate metabolism, In vitro fermentation, Gut microbiota, Microbial ecology, Metabolic competition, Microbial functional potential, Next generation sequencing, Carbohydrate-active enzymes, Meal Ready-to-Eat (MRE)

## Abstract

**Background:**

Interactions between diet, stress and the gut microbiome are of interest as a means to modulate health and performance. Here, in vitro fermentation was used to explore the effects of a sudden change in diet, 21 days sole sustenance on the Meal, Ready-to-Eat (MRE) U.S. military combat ration, on inter-species competition and functional potential of the human gut microbiota. Human fecal samples collected before and after MRE intervention or consuming a habitual diet (HAB) were introduced to nutrient-rich media supplemented with starch for in vitro fermentation under ascending colon conditions. 16S rRNA amplicon and Whole-metagenome sequencing (WMS) were used to measure community composition and functional potential. Specific statistical analyses were implemented to detect changes in relative abundance from taxa, genes and pathways.

**Results:**

Differential changes in relative abundance of 11 taxa, *Dorea, Lachnospira*, *Bacteroides fragilis*, *Akkermansia muciniphila*, *Bifidobacterium adolescentis*, *Betaproteobacteria*, *Enterobacteriaceae*, *Bacteroides egerthii*, *Ruminococcus bromii*, *Prevotella*, and *Slackia,* and nine Carbohydrate-Active Enzymes, specifically GH13_14, over the 24 h fermentation were observed as a function of the diet intervention and correlated to specific taxa of interest.

**Conclusions:**

These findings suggest that consuming MRE for 21 days acutely effects changes in gut microbiota structure in response to carbohydrate but may induce alterations in metabolic capacity. Additionally, these findings demonstrate the potential of starch as a candidate supplemental strategy to functionally modulate specific gut commensals during stress-induced states.

**Supplementary Information:**

The online version contains supplementary material available at 10.1186/s12866-023-02776-2.

## Background

The human gut microenvironment is influenced by complex interactions between the host and gut microbiome. The network of these interactions is crucial to metabolic processes which maintain gut physiology and host health. Gut microbiota community structure can be modulated by host exposure to different stressors including psychological stress, sleep deprivation, environmental factors, and physical activity, which can, in turn, influence host health [1]. Changes in diet are of interest as both a potential source of stress on the gut ecosystem (e.g., a sudden or extreme diet change), but also as an intervention strategy to combat unfavorable stressor-induced shifts in that ecosystem.

Griffin et al. has demonstrated that microbiota responses to a dietary intervention vary among individuals and that metacommunity dynamics (e.g. when an individual’s microbiota is connected to other individuals’ communities by microbial exchange) can have implications in health. These microbiota responses could help on the understanding of how individuals practicing certain type of diets respond to the ingestion of particular foods in a determined period of time. Interestingly, correlations of differences in taxa, composition and richness, can be made with the abundance of diet-specific metabolic and functional biomarkers [2]. Griffin et al. stated that the process of designing probiotic and nutritional interventions includes identifying the microbes associated with specific diets, predicting the responses of the individuals to the diets, and determining if those microbes are related to that dietary practice. It has been shown that modulating the gut microbiota via dietary intervention can reduce symptoms of some metabolic disorders, such as obesity, and associated complications (e.g. systemic inflammation) [3]. Chen et al. presented a literature review focused on gut microbiota therapeutic interventions using diet as the mechanism to understand the cardioprotective effects on heart failure [4]. Haak et al. reviewed the potential benefits of employing the microbiota to treat sepsis [5]. In a recent longitudinal study by Johnson et al., the authors found that the responses to specific diets are highly personalized and although there were detectable changes in the microbiome relative abundances, some of these changes were not highly conserved across subjects [6]. These results could be taken into account at the time of developing dietary intervention practices.

Our group recently reported that fecal microbiota composition was changed after a sudden diet shift, namely a shift from consuming a habitual diet to the US military Meal, Ready-to-Eat (MRE) combat ration over 21 days, resulting in lower relative abundance of multiple genera of lactic acid bacteria (e.g. *Lactobacillus, Lactococcus, Leuconostoc*) and increased relative abundance of several saccharolytic genera (*Streptococcus* and *Clostridium*) [7]. An in vitro fermentation experiment was also used to assess the potential use of resistant starch (RS2) for restoring *Lactobacillus* following MRE consumption. The approach promoted an ideal environment that allowed inter-species competition for nutrients to occur in samples collected before and after the MRE intervention corresponding to a control group studied over the same time scale of hours, which is not feasible in in vivo human studies which commonly rely on daily stool samples. *Ruminococcus bromii*, a keystone taxon and resistant starch degrader, increased in relative abundance during the MRE diet in the presence of RS2 while the ability of *Lactobacillus* to compete in presence of RS2 appeared to be reduced [8]. However, those results were limited in that only a few selected taxa were measured, the identity of which *Lactobacillus* species affected could not be elucidated, and differences in functional capacity of the community could not be examined nor the whole community metabolic response to RS both compositionally and functionally. The results did provide initial insight into how in vitro studies can complement human study results and reveal microbial community functional understanding in response to stress.

Herein we report a comprehensive genomic analysis employing both 16S rRNA gene amplicon and Whole-metagenome sequencing (WMS) of samples collected during the in vitro fermentation experiment described in Pantoja-Feliciano et al. 2019 [8] to reveal influence of a sudden change in diet on whole bacterial community composition and functional potential. To explore stress-induced microbial community responses, carbohydrate content, specifically RS, in medium was increased five-fold to allow the study of nutrient:microbiome interactions that cannot easily be explored in vivo [8].

## Results

### Stressor-induced changes to microbial composition

Temporally sampled fecal aliquots were extracted to isolate DNA for 16S rRNA gene amplicon (16S) sequencing. A total of 65 samples were analyzed and 36 564 861 demultiplexed sequence counts obtained. After alpha-diversity analysis, differences in Faith’s Phylogenetic Diversity (PD) by Diet (Fig. S1A) and Study Day (Fig. S1B) were not evident; however, there were decreases in alpha diversity as the fermentation progressed (Fig. S1C), as shown by the PD plots. There were no observed differences in the interaction Diet*Study Day (Fig. S1D). Same pattern was observed when choosing a sequencing depth of 212,889 to generate Boxplots for Diet (Fig. S2A Panel a), Study Day (Panel b), Fermentation Time points (Panel c), and Diet_Study day (Panel d) for Faith_PD metric. Supplementary Figs. S3 and S4 show similar results for Observed OTUs (metric computed by default in QIIME2-2019.7 version) and Shannon Diversity Plots, respectively.

**Fig. 1:**
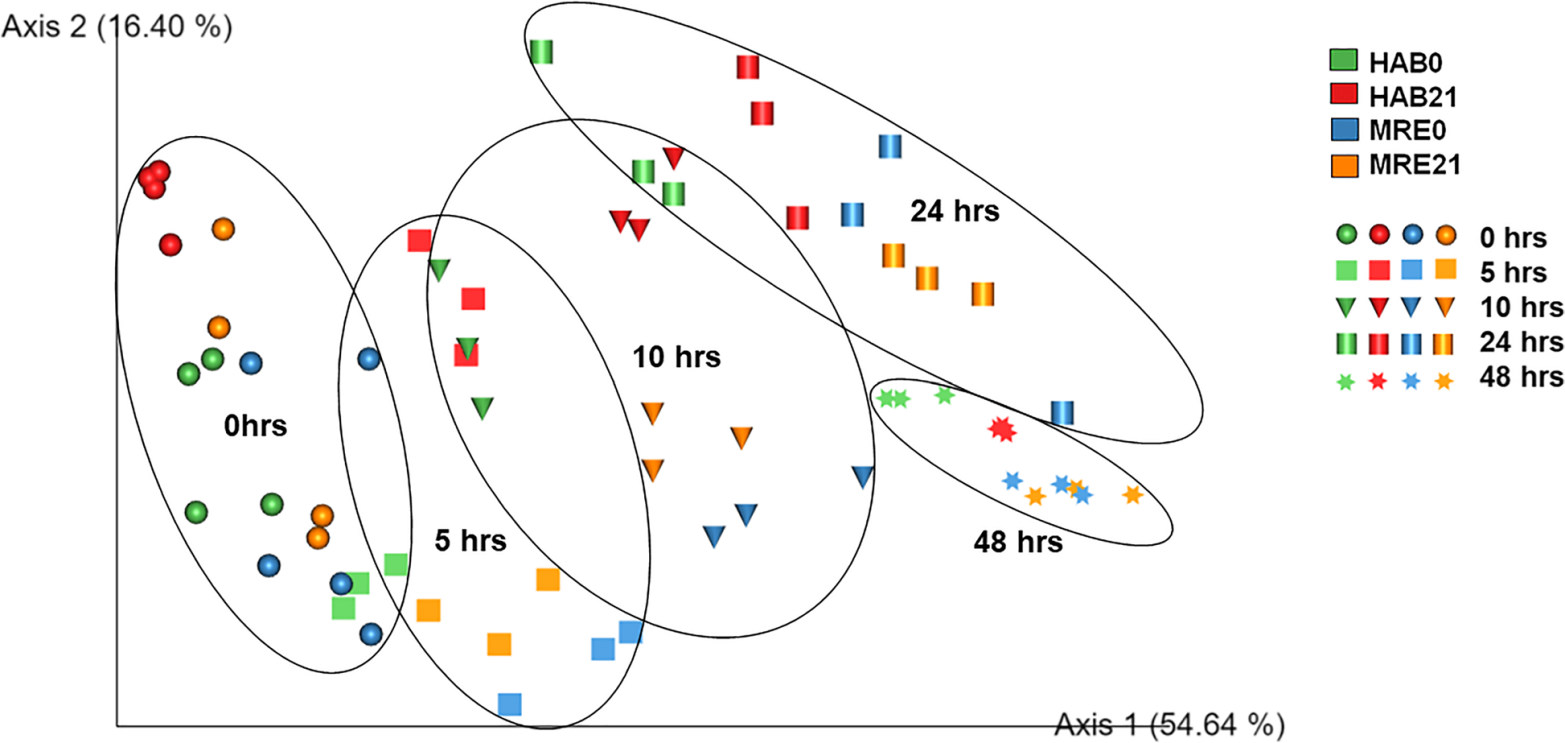
16S rRNA Weighted-Unifrac PCoA shows a divergence and clustering by Fermentation Time Points (0, 5, 10, 24, and 48 h) but not by Diet (MRE vs HAB) and Study Day (0d vs 21d)

Beta-diversity analysis represented by Weighted-Unifrac PCoA (Fig. 1) illustrated clustering by fermentation time points but not by Diet_Study day (HAB0, HAB21, MRE0, MRE21). Microbial communities from samples at 24 and 48 h after exposure to RS-supplemented medium appear to be similar in composition and clustered closer as observed in the PCoA (Fig. 1). As a result of the PCoA showing a close clustering of 24 h and 48 h samples, 48 h samples were excluded from further statistical analysis. The decision of excluding 48 h samples was made based on those results that showed 24 h and 48 h samples clustering together with significant similarity in terms of microbial communities. Also, within batch fermentations, 24 h represents stationary phase and any time points beyond 24 h tend to begin exhibiting degrees of proteolysis. The 48 h was only included to indicate a full gut transit cycle of 24-48 h and 24 h proved optimal for bioinformatic and biostatistical analysis. These factors provided the rationale for excluding 48 h as that time point does not add any additional information to the primary outcomes of the work.

**Fig. 2:**
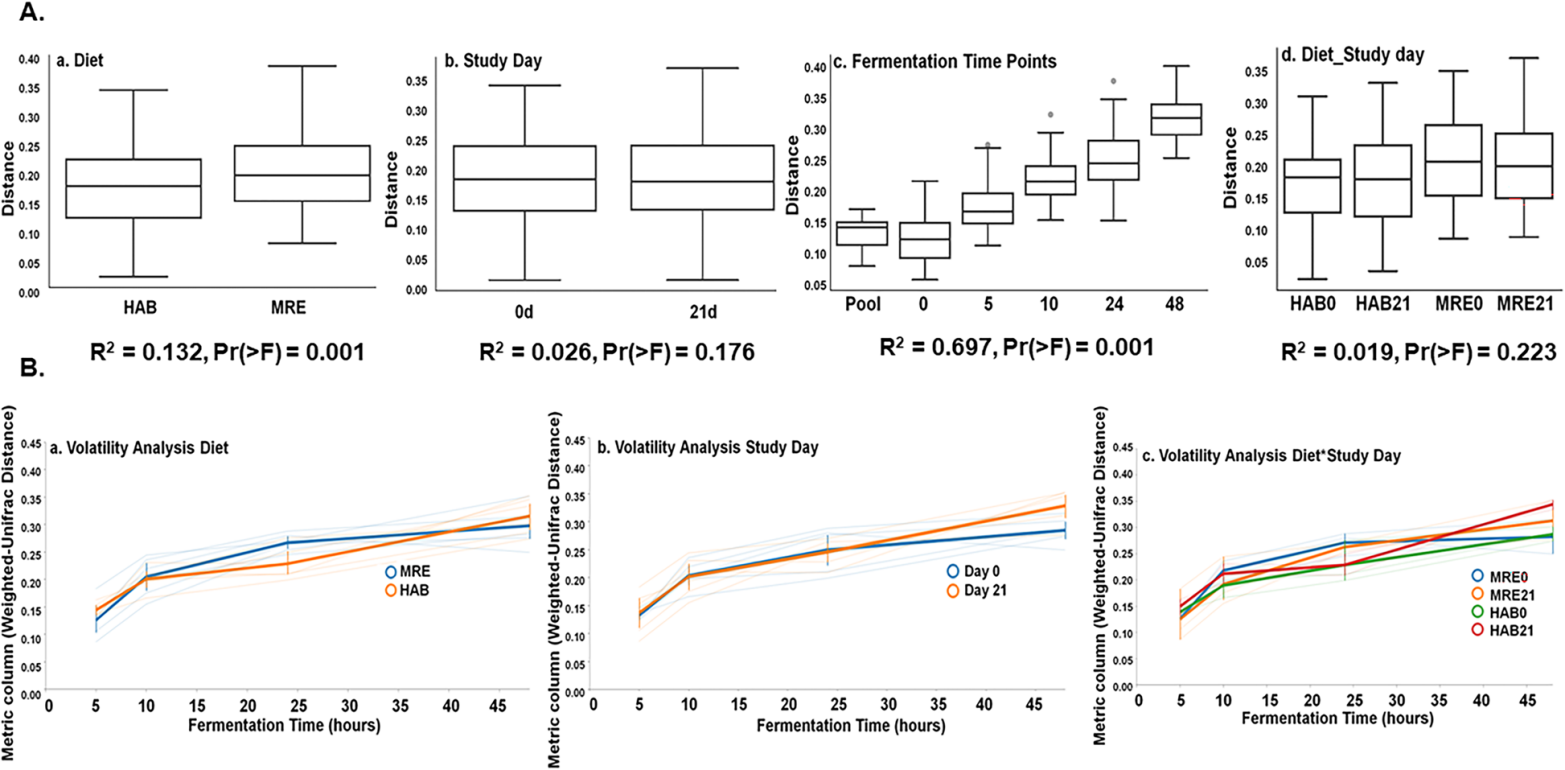
Weighted-Unifrac Distances Metric Boxplots with PERMANOVA Analysis (**A**) and Beta-Diversity Volatility Analysis (**B**). Panel A corresponds to the Weighted_Unifrac Distance Metric and Adonis PERMANOVA analysis by Diet (a), Study Day (b), Fermentation Time Points (c) and Diet_Study Day (d) groups, respectively. *P*-values are for comparisons to the Pool time point. Panel B corresponds to the Volatility Analysis for the Beta-Diversity Weighted_Unifrac Distance Metric for (a) Diet, (b) Study Day and (c) Diet_Study Day

Weighted-Unifrac Distances Metric Boxplots and Adonis PERMANOVA analysis by diet (PERMANOVA, R^2 =^ 0.13, Pr(> F) = 0.001), study day (PERMANOVA, R^2 =^ 0.03, Pr(> F) = 0.18), fermentation residence time (i.e. how long the bacteria is in the medium) (PERMANOVA, R^2 =^ 0.70, Pr(> F) = 0.001), and distances between diet and study day together (PERMANOVA, R^2 =^ 0.02, Pr(> F) = 0.22), are represented in Fig. 2A, Panels a, b, c, and d respectively. Diet alone has a visible effect on the microbial community as presented by the PCoA and in the PERMANOVA Analysis, but from the PCoA we can see that there is not a clear separation of the groups corresponding to the interaction of diet with study day (which we call HAB0, HAB21, MRE0, MRE21 as groups; the number in this nomenclature refers to the day on each diet: 0 before starting, 21 to 21 days after starting the diet), and the R^2^ in Fig. 2 panel d is 0.019, with no statistically significant difference (Pr > F) = 0.223. Unstable microbiome could be linked to harmful responses to stress [9]. Volatility analysis was computed for alpha and beta diversity (Faith_PD and Weighted-Unifrac metrics, respectively) to see if there were differences in volatility based on our stressor, the diet. For alpha diversity, differences were not apparent for Diet, Study Day and Diet_Study Day (Fig. S2 B Panel a, b, c respectively). For beta diversity, differences were visible in Diet and Diet_Study Day at 24 h of fermentation (Fig. 2 B Panels a and c, respectively).

In total, 201 taxa were identified by 16S but a visible pattern of differences in community structure due to diet, date or fermentation residence time is not evident in the taxonomy bar plot (Fig. S5).

Features that were present in less than half of the samples in all diet-day combinations at 0 h (start of fermentation) were removed. The remaining 127 taxa were arcsine transformed. A linear mixed model analysis using a multivariate ANOVA with repeated measures was employed to the 127 taxa to detect abundance variations as a function of the MRE-diet intervention (Diet*StudyDay*Fermentation Time interaction). 52 Taxa were identified as having a statistically significant interaction between diet, study day, and fermentation residence time as shown in Table S1. *Dorea*, *Lachnospira, Bacteroides fragilis, Akkermansia muciniphila, Bifidobacterium adolescentis Betaproteobacteria, Enterobacteriaceae, Bacteroides eggerthii, Ruminococcus bromii, Prevotella, and Slackia,* showed interesting patterns of MRE21 relative to the other groups (HAB0, HAB21, MRE0), mainly to MRE0 (Table S2). Patterns comparing other groups (not focusing on MRE21 as the main group) are not included for further analysis and discussion.

**Fig. 3:**
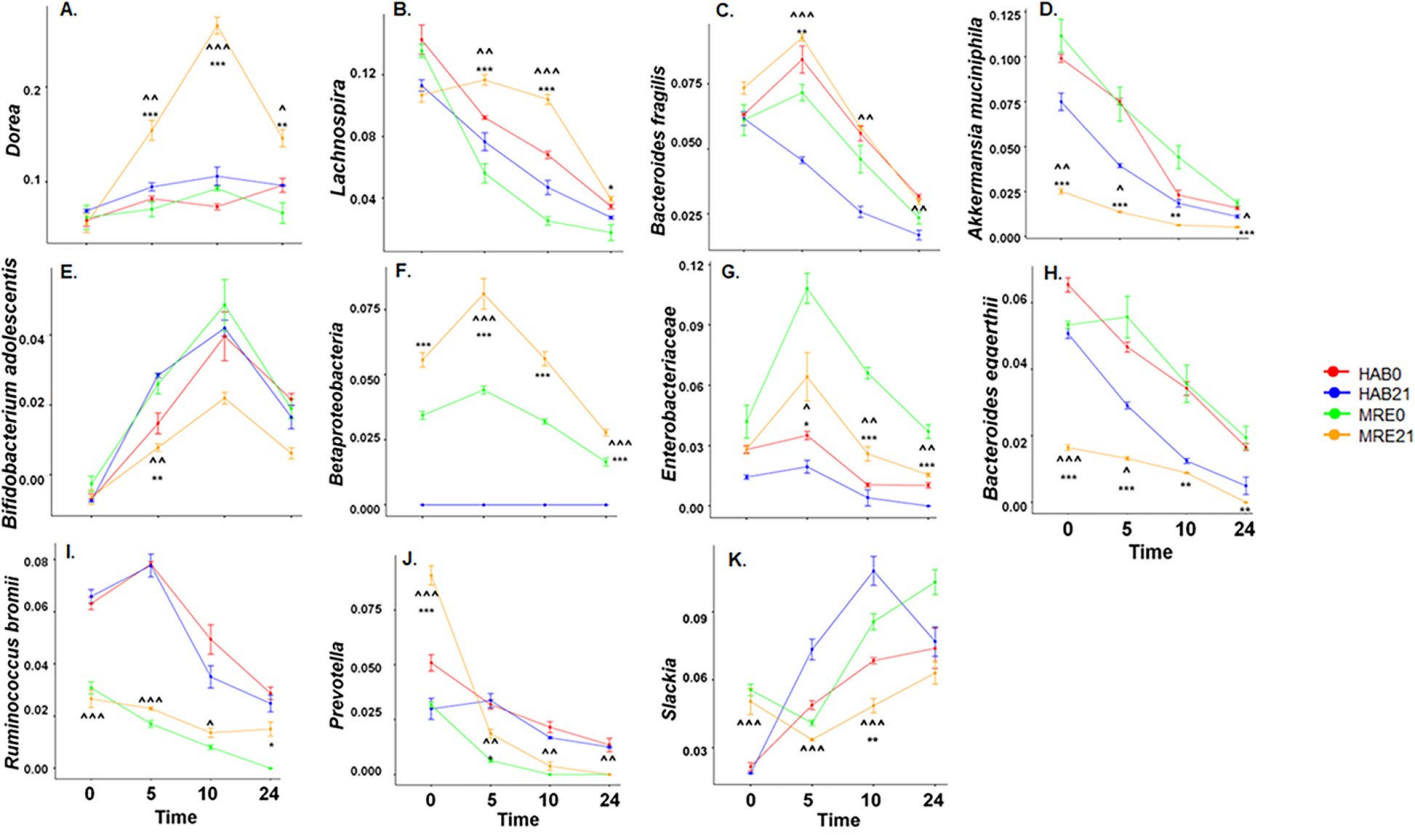
16S rRNA Linear Mixed Model Analysis for 11 organisms. Linear mixed model analysis (multivariate ANOVA with repeated measures) for Diet*StudyDay*Fermentation Time Points interaction. Linear graphs representing the 11 organisms out of 127 that have significant 3-way interaction for the different fermentation time points and Diet/Date groups in function of their relative abundance: Dorea (**A**), Lachnospira (**B**), Bacteroides fragilis (**C**), Akkermansia muciniphila (**D**), Bifidobacterium adolescentis (**E**), Betaproteobacteria (**F**), Enterobacteriaceae (**G**), Bacteroides egerthii (**H**), Ruminococcus bromii (**I**), Prevotella (**J**), and Slackia (**K**). Exact p-values from the test are also reported in Table S1. Pairwise multiple comparison (Tukeys HSD Analysis) for the 11 organisms obtained after the Linear mixed model analysis within each time-point, is represented by symbols: a (*) symbol indicates a difference between study days 0 and 21 for the same diet, and a (^) symbol indicates a difference between MRE and habitual (HAB) diets for the same study day. One symbol indicates *p* ≤ 0.05, two symbols indicates *p* ≤ 0.01, and three symbols indicates *p* ≤ 0.001. Exact p-values from the test are also reported in Table S2

*Dorea* spp. notably increases in the MRE day 21 group after 10 h of exposure to starch-supplemented medium relative to the other groups (Fig. 3A). *Lachnospira* also showed a notable increased in relative abundance in the MRE21 group relative to the other groups at 5 and 10 h of fermentation (Fig. 3B). *Bacteroides fragilis* (Fig. 3C) was statistically higher in MRE21 compared to MRE0 at 5 h of fermentation as represented by the Tukey analysis (Table S2). *Akkermansia muciniphila* was diminished in relative abundance in MRE day 21 compared to HAB diet day 0 and 21 and MRE day 0 throughout the fermentation; however, the rate of change over the course of fermentation differed in MRE day 21 compared to MRE day 0 (*p* < 0.050) as determined by an equality of slopes test (Fig. 3D, Fig. S6D). A similar case was observed for *Prevotella* where abundance at inoculation was higher but decreased as the fermentation proceeded (Fig. 3J); however, differences between MRE day 0 and MRE day 21 were not detected at later time-points, and after 24 h residence time, this organism was completely diminished in both groups (Fig. S6J). In addition, *Bacteroides adolescentis* showed no differences at the inoculation time point in MRE21 compared to the other groups but showed a notable decrease at 5 and 10 h of fermenation in the MRE21 group relative to MRE0 (Fig. 3E). *Betaproteobacteria* showed a higher relative abundance pattern in MRE21 when compared to the other groups (Fig. 3F). It was higher at inoculation time compared to MRE0 but the equality of slopes test showed that the rate of change over the course of fermentation didn’t differ in MRE day 21 compared to MRE day 0 (Fig. S6F). *Enterobacteriacea* family (Fig. 3G) presented a lower relative abundance in the MRE21 group as compared to MRE0 but higher than HAB21 (Table S2). Another species that showed an interesting pattern was *Bacteroides eggerthii* (Fig. 3H), which showed diminished abundances in MRE21 over the course of the fermenation compared to the other groups. In addition, the equality of slopes test showed that the rate of change over the course of fermentation differed in MRE21 relative to the other groups (Fig. S6H). At inoculation, *Ruminococcus bromii* were not different comparing MRE21 to MRE0 but at 24 h its relative abundance increased as confirmed by the Tukey analysis (Fig. 3I, Table S2). The equality of slopes test showed that the rate of change over the course of fermentation differed in MRE21 relative to the other groups (Fig. S6I). *Slackia*, our last interesting group to mention (Fig. 3K), was significantly lower in MRE21 compared to MRE0 at 10 h of fermenation (Table S2).

### Stressor-induced changes to Microbial Functional Potential

Whole-metagenome sequencing (WMS) was employed to complement 16S analyses and explore the influence of MRE-diet intervention at a functional level. Though the diet*study day interaction only resulted in subtle alterations in community composition as assessed by 16S sequencing, genomes for strains that have nearly identical ribosomal RNA sequences have been shown to possess different functional capabilities [10]. Using the assembly free program HUMAnN2 [11], we assessed community wide function and observed similar clustering patterns as those seen with 16S. PCoA of Bray–Curtis distances in gene family abundances (Fig. S7A) resulted in the first principal coordinate associating with fermentation time (PERMANOVA, R^2^ = 0.23, *p* = 0.001). The second principal coordinate was associated with diet (PERMANOVA, R^2^ = 0.17, *p* = 0.001) and there was a small effect of Diet*Date (PERMANOVA, R^2^ = 0.03, *p* = 0.001). At the pathway functional level (Fig. S7B), PCoA of Bray–Curtis distances in pathway abundances primarily clustered by fermentation time (PERMANOVA, R^2^ = 0.31, *p* = 0.001) with effects of Diet (PERMANOVA, R^2^ = 0.15, *p* = 0.001) and Diet*Date (PERMANOVA, R^2^ = 0.02, *p* = 0.001).

To parse whether there were finer scale differences for specific strains or functions, sequences from all samples were co-assembled, binned into metagenome assembled genomes (MAGs), and functionally annotated. After binning, we assembled 120 MAGs at > 50% completion and < 10% contamination including 57 MAGs at > 90% completion and < 5% contamination and 63 MAGs at > 50% completion, < 10% contamination. PCoA of Bray–Curtis distances for MAG abundances showed similar clustering by diet and fermentation residence time to the 16S PCoA (Fig. S8A). The first principal coordinate was associated with fermentation time (PERMANOVA, R^2^ = 0.27, *p* = 0.001) and the second with diet (PERMANOVA, R^2^ = 0.23, *p* = 0.001). There was a small, significant effect of Diet*Date (PERMANOVA, R^2^ = 0.03, *p* = 0.001). Thus, at the MAG level, there was not as large of an effect of MRE diet when comparing Day 0 to Day 21 (Diet*Date interaction) as compared to the effects of fermentation time and diet alone.

**Fig. 4:**
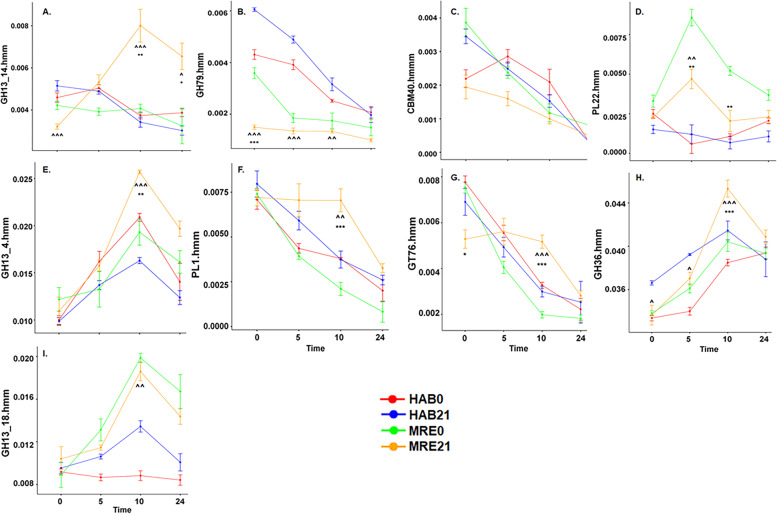
Linear Mixed Model Analysis for 9 CAZymes. Linear graphs representing the 9 CAZymes out of 20 that have significant 3-way interaction for the different fermentation time points and Diet/Date groups in function of their relative abundance: GH13_14 (**A**), GT79 (**B**), CBM40 (**C**), PL22 (**D**), GH13_4 (**E**), PL1 (**F**), GT76 (**G**), GH36 (**H**), and GH13_18 (**I**). (*) symbol indicates a difference between study days 0 and 21 for the same diet, and a (^) symbol indicates a difference between MRE and habitual (HAB) diets for the same study day. One symbol indicates p ≤ 0.05, two symbols indicates *p* ≤ 0.01, and three symbols indicates *p* ≤ 0.001

**Fig. 5:**
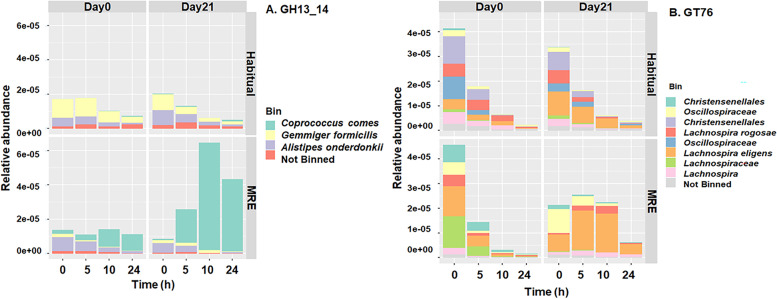
CAZyme’s Bar Plot by Species. Bar graphs linking CAZymes relative abundances with bacterial species bins. **A** corresponds to GH13_14, and **B** GT76. MAG and taxonomic breakdown of GH13_14 by Diet*Date indicated that the increased abundance in MRE Day 21 samples was due to a Coproccocus comes MAG (**A**) and CAZyme GT76 and its prevalence in the MRE day 21 group associated with Lachnospira eligens (**B**)

Due to the inclusion of starch supplementation during fermentation, we assessed whether specific functions for complex carbohydrate breakdown (Carbohydrate-active enzymes, CAZymes) were affected by Diet*Date. Hidden Markov models were used to identify and classify CAZymes in the metagenome assembly. PCoA of Bray–Curtis distances for CAZyme abundances showed similar clustering patterns to the pathway PCoA (Fig. S8B). The first principal component was primarily associated with fermentation time (PERMANOVA, R^2^ = 0.57, *p* = 0.001) with small effects of Diet (PERMANOVA, R^2^ = 0.06, *p* = 0.001) and Diet*Date (PERMANOVA, R^2^ = 0.03, *p* = 0.001). To uncover CAZymes which have abundance variations as a function of the MRE-diet intervention (Diet*StudyDay*Fermentation Time interaction), we employed a linear mixed model to 300 CAZymes after arcsine transformed (Fig. 4). 20 CAZymes passed the significance test (Table S3) and 9 showed interesting patterns due to MRE21 group (Fig. 4). Table S4 shows the p-values corresponding to the pairwise multiple comparison analysis Tukey HSD for each fermentation time point in the different groups, supporting Fig. 4. In the case of GH13_14, MRE day 21 group was significantly different from the other groups at 10 and 24 h after fermentation (Fig. 4A). GH13_14 was of particular interest because these enzymes catalyze the cleavage of branched RS2 breakdown products. MAG and taxonomic breakdown of GH13_14 by Diet*Date indicated that the increased abundance in MRE Day 21 samples was due to a *Coproccocus comes* MAG (Fig. 5A). Another interesting case was the CAZyme GT76 (Fig. 4G) and its prevalence in the MRE day 21 group associated with *Lachnospira eligens* (Fig. 5B). Correlation with selected CAZymes (e.g. GH13_14 and GT76) to taxa were completed but additional correlations were outside the scope of this paper.

The other CAZymes that showed interesting patters in MRE21 group were GT79 (Fig. 4B) which showed significantly lower relative abundances in MRE21 differences compared to the other groups (Tukey Table S4); PL22 CAZyme showing decreases in MRE21 at 5 and 10 h compared to MRE0; three CAZymes showed similar patterns of increased relative abundances in MRE21 at 10 h of fermentation to the CAZyme previously discussed GH13_14, namely GH13_4, GH36 and GH13_18 (Fig. 4E, H and I, respectively). Another type of CAZyme, PL1, showed MRE21 increases at 10 h as well (Fig. 4F). Although CBM40 (Fig. 4C) seems to be diminished at 5 h of fermentation in the MRE21 group, Tukey analysis didn’t confirm statistically significant differences between groups (Table S4). Thus, the MRE diet did result in subtle functional differences at the fine-scale CAZyme level.

Similar analysis was employed to find pathways that were important for differentiating Diet*StudyDay*Fermentation Time categories. A Linear Mixed Model approach was employed to 280 pathways after arcsine transformation. As a result of the analysis, no significant features relative to MRE21 were evident. However, we included analysis in Supplementary (Table S5).

## Discussion

This study used an in vitro fermentation system that simulated the conditions of the gut, to examine the effects of starch supplementation on gut microbial community composition and functional capacity in samples collected from volunteers that consumed two different diets, the Meal, Ready-to-Eat (MRE) U.S. military combat ration and a habitual diet (HAB) for 21 days. Subtle changes in gut microbiota structure and metabolic capacity in response to RS2 were observed as a result of MRE diet consumption, suggesting that the MRE diet does not substantially influence competitive dynamics within the gut microbiome for the model substrate. With the incorporation of the volatility concept, the degree of compositional changes in the microbiome due to a specific stress could be measured over time [9]. Here, we applied volatility to alpha and beta diversity data and in effect we observed differences in beta diversity in response to diet, in contrast to alpha diversity where no responses were apparent.

Several studies have identified changes in microbial communities due to RS consumption. Martinez et al. has previously shown fecal microbiota composition changes after a RS2 diet in a human study, specifically a significant increase in the *Ruminococcus bromii* and *Eubacterium rectale* proportions [12]. A more recent study reported gut microbiota changes in mice when introducing a RS diet. Researchers observed a significant increase in members of the *Proteobacteria* and *Verrucromicrobia* phyla correlated to an observed increase in anxiety-like behaviors within the animals [13]. These are contrary to one part of our results in which *Akkermansia muciniphila* (*Verrucromicrobia*) decreased upon exposure to RS-supplemented medium but in concordance with *Betaproteobacteria* (*Proteobacteria*) which presented higher relative abundances. *Enterobacteriaceae* in our study showed an increase at 5 h of fermentation in MRE21 group but still was lower when compared to the group MRE0. The discrepancy may be due to differences in study design, comparing results from human samples to an animal model results, fermentation conditions and interindividual variability of the volunteers.

Although ~ 200 taxa were identified by 16S rRNA sequencing, only 11 bacterial groups showed differential changes in relative abundance during the fermentations as a function of the diet intervention. These groups were *Dorea*, *Lachnospira, Bacteroides fragilis, Akkermansia muciniphila, Bifidobacterium adolescentis Betaproteobacteria, Enterobacteriaceae, Bacteroides eggerthii, Ruminococcus bromii, Prevotella, and Slackia,* from which *Dorea*, *Lachnospira, Betaproteobacteria, Enterobacteriaceae* and *Slackia* are different than those identified in the previously reported qPCR analysis from the equivalent sample set. The other groups are consistent between studies [8]. Reasons for the inconsistency are unknown but likely due to the more comprehensive nature of the 16S and metagenomic analysis in this study rather than using qPCR to targeted specific species as was done in the previous study. The taxa identified by 16S in the present analysis, however, represent gut commensals that have a range of clinical and physiological relevance. For some of them, *Dorea, Lachnospira, Bacteroides fragilis, Bifidobacterium adolescentis,* and *Enterobacteriacea*, inter-species competition for RS were significantly altered following 21d on the MRE diet. *Dorea* spp are members of the Clostridium cluster XIVa and are a dominant species in the human gut [14]. *Dorea* spp utilizes dietary carbohydrates such as simple sugars (eg, glucose, lactose, maltose), inulin and fructo-oligosaccharides to produce metabolic products including acetate, formate, lactate and ethanol [15]. Although some species are unable to directly metabolize starch, it is associated with starch absorption in mice, perhaps through cross-feeding on small hydrolysis products (e.g., maltose, glucose) from initial starch degradation by starch-degrading taxa [16], including possibly *Prevotella* whose abundance was increased due to the MRE-diet relative to the HAB diet. *Dorea* is considered part of a healthy gut microbiota, although it has also been shown to have increased abundance in Multiple Sclerosis and IBD patients [17]. *Lachnospira* has been identified as one of the core groups of the gut microbiota [18]. Together with other gut members, *Lachnospira* abundances could increase with a diet rich in fiber [19]. In a previous study, Bang et al. 2018 investigated how the gut microbiota utilizes pectin, a fiber found in fruits and vegetables, through in vitro fermentation and metagenomics analysis [20]. They focused on *Faecalibacterium* and *Lachnospiraceae*, which has been demonstrated to express carbohydrate-active and pectin-degrading enzymes. As one of the main results, they observed that with an increased incubation time with pectin, *Lachnospira*, *Sutterella*, *Dorea* and *Clostridium* increased. Additionally, it’s been shown that *Lachnospira* and *Prevotella* can utilize complex carbohydrates, Microbiota Accessible Carbohydrates (MAC), to produce CO_2_, H_2_ and short chain fatty acids (SCFAs) to improve energy metabolism and ameliorate conditions like inflammatory bowel disease and asthma [21]. *Bacteroides* species are the most abundant microorganisms in the human gut [22]. They are saccharolytic bacteria that could be symbionts or mutualists with some species contributing to the development of the immune system, but under certain conditions they could be opportunistic pathogens [23]. *Bacteroides fragilis* is an example of enterotoxigenic strains that secrete a toxin causing a virulence factor that can induce intestinal inflammation, something that has been implicated in colorectal cancer [24]. Rios-Covian et al. 2015 showed that *B. fragilis* was able to grow in the presence of glucose and exopolysaccharides (EPS), complex carbohydrate polymers that some bifidobacterial species produce [25]. *Bifidobacterium adolescentis* is an amylotic bacteria found in the human large intestine [26] and is one of the most abundant species of bifidobacterial in the human colon [27]. The probiotic is able to utilize glucose, maltose, panose and isomaltose [26] and starch or d-fructo-oligosaccharides (FOS) as growth substrates [28]. *Enterobacteriacea* is a family that belongs to the *Proteobacteria* phylum in which some pathogens could be identified (e.g. *Escherichia coli*).

Taken together, it is not clear whether the effect of the MRE diet on *Dorea, Lachnospira, Bacteroides fragilis, Bifidobacterium adolescentis,* and *Enterobacteriacea* responses to RS2 we observed in this study can be considered beneficial for the microbial community or the host. For the other taxa, *Akkermansia muciniphila, Betaproteobacteria*, *Bacteroides eggerthii*, *Ruminococcus bromii*, *Prevotella* and *Slackia*, their relative abundance differed in the MRE-day 21 samples at the 0 h time point, but changes over time during the fermentation did not differ as a function of diet. This suggests an effect of the MRE diet on those taxa, but not the response of those taxa to RS2. Though that result does not match the previous 16S compositional analysis from the human study [7], this is likely due to only a sub-population (n = 5 volunteers per group) being used in the in vitro experiment rather than all 30 study volunteers. The subtle distinctions within the findings are difficult to draw conclusions associated with these taxa. *A. muciniphila* has been previously associated with beneficial health outcomes [29, 30] and has been shown to metabolically respond to high RS diet in rats [31] and humans [32]. *Betaproteobacteria* belongs to Proteobacteria, a phylum that contains some important pathogens; *Bacteroides eggerthii* has been identified in human and fish feces and is associated with CAZymes expression, serving, in general, as a metabolic symbiotic for other gut commensals lacking sugar utilization systems [23, 33, 34]; *Ruminococcus bromii* is an amylolytic bacteria, starch-degrading keystone species in the human colon, which has a preference for α(1–4)-linked oligosaccharides larger than maltose, as it was shown to grow faster in rumen fluid medium on maltotriose or maltotetraose, and unable to utilize glucose [26]. *Prevotella* spp. is thought to be beneficial due to its prevalence in a high fiber diet and has also been shown at the family level to increase following high RS diet [32] and during in vitro fermentation studies [35, 36]. *Slackia* is a gut bacteria that plays a role in host lipid and xenobiotic metabolism and some species are capable of the conversion of isoflavone daidzein to equol and/or *O*-desmethylangolensin (O-DMA) [37]. Some of these taxa warrant further investigation as bacterial targets for RS supplementation.

WMS analysis identified several CAZymes, GH13_14, PL22, GH13_4, PL1, GT76, GH36 and GH13_18, that differentially changed in response to RS following MRE consumption relative to other samples. Enrichment of the extracellular glycan-active enzyme glycoside hydrolase (GH13_14) was associated to *Coprococcus comes*, a member of the Clostridium cluster XIVa [38]. GH13_14 is a pullulanase common in human gut lactobacilli. As a butyrate producer, *Coprococcus comes* is generally thought to be beneficial. It has also been negatively correlated in type 1 diabetes patients [39]. Maier et al. has shown CAZymes and transport systems related to *C. comes* have increased in abundance in response to an RS diet [32]. *Lachnospira eligens* was related to the CAZyme GT76. *L. eligens* utilizes pectin and polygalacturonic acid, with acetate, formate, ethanol, and CO_2_ as major end products [40]. It has been associated with the glycosyltransferase GT76, a α-1, 6-mannosyltransferase that uses dolichol-P-mannose as a sugar donor. GT’s are enzymes that forms glycosidic bonds and are involved with biosynthesis of di-, oligo-, and polysaccharides (www.cazy.com). Both *C. comes and L eligens* were not directly identified in the 16S rRNA analysis at the species level but the genus *Coprococcus*, *Lachnospiraceae* family and *Lachnospira* were identified as part of the 30 most abundant taxa in the taxa bar plot. These microbes were not significant specifically after the LMM analysis, which may suggest that not all species within each genus and family respond in the same way and also highlights the value of a higher level of resolution provided by WMS compositional and functional analysis. Otherwise, these taxa significantly contributed to CAZyme alterations and community carbohydrate metabolism in the presence of RS2. CAZymes GH13_4 and GH13_18 are glycoside hydrolases belonging to the Family 13, subfamily 4 and 18 respectively, and showed similar results in MRE21groups as the CAZyme GH13_14 that was previously discussed. GH36 is a glycoside hydrolase too but belongs to the Family 36. GH79 belongs to the same family but subfamily 79. It was the only CAZyme in our study that significantly started at the inoculation time with different relative abundances in MRE21 compared to the other groups but continued steady over the course of the fermentation. PL22 is part of the Polysaccharide lyases (PL) group of CAZymes that are involved in degradation of plant cell walls, with major activities as oligogalacturonate/oligogalacturonide lyase and isolated from *Bacteroides* species, including *B. fragilis* [41]. It was found to be enriched in *Escherichia* in a study that was seeking to understand the development of the gut microbiome and succession in infants [42]. It was involved in activities such as metabolizing small molecules (sugar bioproducts of mucin) and dietary polysaccharides degradation. PL1 is also part of the PL group with main role in pectate lyase [41].

The study was limited by use of only a subset of volunteers within the in vitro fermentation studies, pooling the samples that limited individualized study outcomes and a lack of correlative SCFA, metabolomics and proteomics analysis to corroborate bioinformatics findings. However, the data does demonstrate sudden changes in diet had a functional effect on community competition for RS and that certain potentially beneficial taxa respond to RS supplementation differentially as a function of diet and stress.

## Conclusion

In this study, we used in vitro fermentation to explore the effects of an acute stressor, a sudden change in diet from habitual to sole sustenance on MREs, on inter-species competition dynamics of gut microbiome in response to starch supplementation. There were no evident clusters in the PCoA as a function of diet and the deeper multivariate analysis indicated MRE consumption does not appear to substantially impact the effects of RS2 on the gut microbiome. Rather, only minimal alterations in community composition and functional potential as measured by CAZyme relative abundance were observed, relative to the total taxa and CAZymes identified. These results did demonstrate that community metabolic capacity and competition for substrates can be altered even when taxa abundance are not significantly different in the absence of that substrate. The findings demonstrate the value of combining human microbiome studies, in vitro fermentation, and powerful next generation sequencing techniques like 16S and WMS to effectively gain a more complete understanding of the effects of stress on competitive nutrient:microbiome:interactions and to identify potential strategies toward modulating gut commensal metabolic competition during stress states.

## Methods

### Participants

Fecal samples were collected from ten individuals participating in a randomized controlled trial designed to determine the effects of subsisting on a MRE-only diet on gut microbiota composition and intestinal permeability [7]. For more information about the characteristics of the participants and their diets see Supplemental Table 1 from Pantoja-Feliciano et al. 2019. Study details and primary findings have been previously reported (Karl 2019). Briefly, the full study population included 64 adults without obesity, 18–62 yr who were randomly assigned to follow their normal habitual diet for 21d (HAB) or consume a provided diet containing only the MRE rations for 21d. Study exclusion criteria included: use of antibiotics or colonoscopy within 3 mo of enrollment, vegetarian diet, history of gastrointestinal (GI) disease, infrequent bowel movements (< 4x/wk), and habitual use of medications affecting GI function (e.g. laxatives, anti-diarrheals). All participants were instructed to discontinue use of probiotic, prebiotic, or other dietary supplements ≥ 2 wk before study participation. Study involvement was voluntary, and written informed consent was obtained prior to enrollment. The study was reviewed and approved by the US Army Research Institute of Environmental Medicine Human Institutional Review Board (Natick, MA). Investigators adhered to the policies regarding the protection of human subjects as prescribed in Army Regulation 70–25, and the research was conducted in adherence with the provisions of 32 CFR Part 219. The parent study from which samples used in these experiments was registered on www.clinicaltrials.gov as NCT02423551. All volunteers provided written informed consent for their samples to be used for the in vitro experiments described herein.

### Fecal samples

Fecal samples were collected at baseline (day 0) and at the end of the 21d MRE intervention period (day 21). Samples were collected into provided 650 mL collection containers to which an anaerobic sachet (GasPak EZ Anaerobe Container System; Becton, Dickinson and Co., Franklin Lakes, NJ) was immediately added. The sealed container was then kept on ice or in a refrigerator until processing [43]. Fecal slurry (20%) was prepared within 12 h of donation by addition of 0.1 M phosphate buffer pH 7.2 supplemented with 15% w/v glycerol and 0.08% L-cysteine (Sigma-Aldrich; St. Louis, MO), to fresh feces in a 4:1 ratio, followed by homogenizing for two minutes in a Seward Ltd. Model 400 stomacher (Davie, FL). The slurry was anaerobically divided into aliquots and stored at -80 °C until needed.

### *Fermentation System Protocol (Fig. **6**)*

**Fig. 6:**
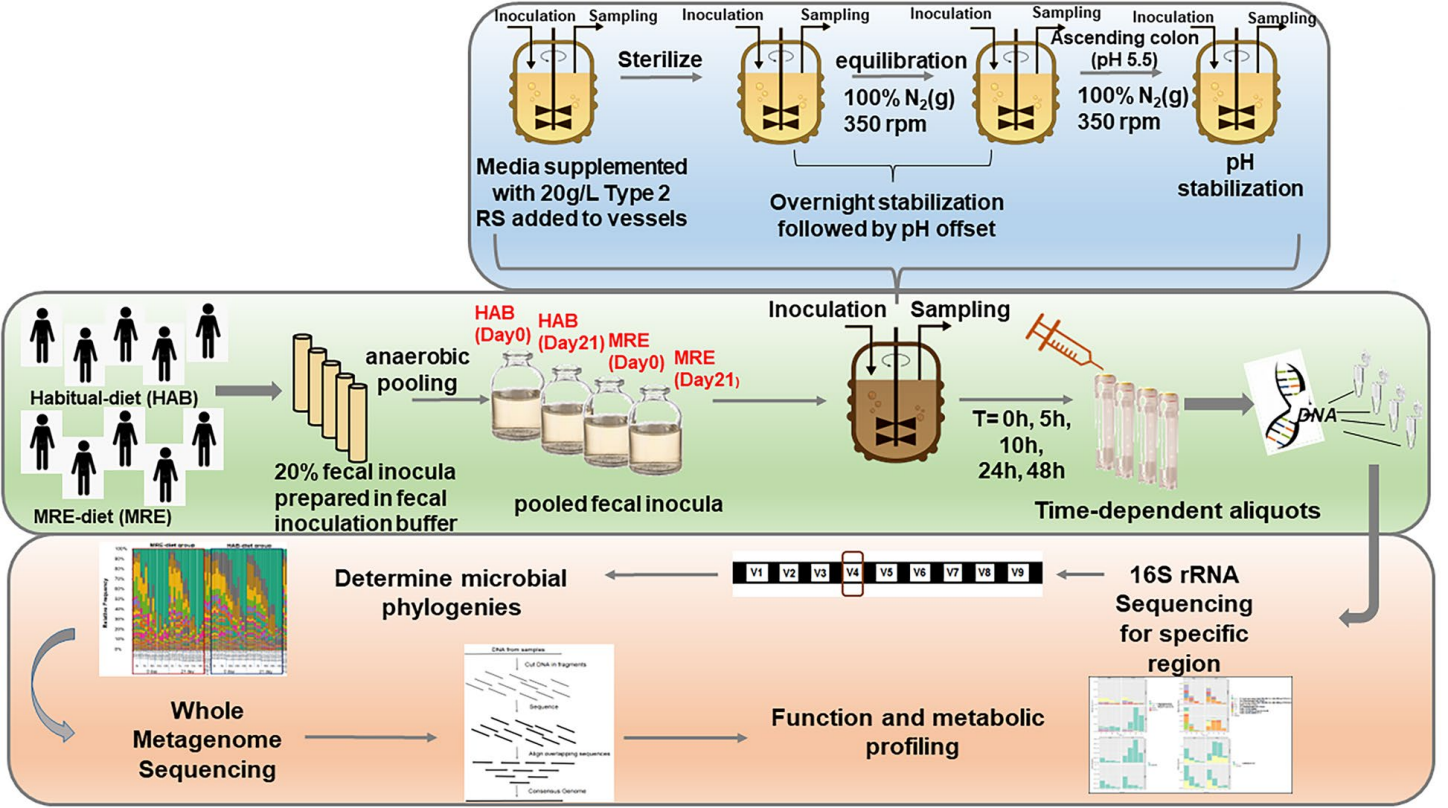
Schematic representation of the in vitro fermentation protocol. Human fecal samples from volunteers belonging to the two different diets were obtained and processed to meet the requirements for the in vitro fermentation system protocol. The top section represents the specific conditions of the system mimicking the human gut. The lower section represents the post-fermentation high-throughput sequencing analysis employed to samples collected at different time points during fermentation. *HAB: Habitual Diet; MRE: Meal, Ready-to-Eat

All chemicals were obtained from Sigma-Aldrich unless otherwise indicated. The fermentation parameters are outlined in Pantoja-Feliciano et al. [8]. Briefly, fermentation medium was prepared based on Macfarlane et al.[44] with the following modifications: addition of resazurin (1 ug/L) and supplemented with a fivefold increase in potato resistant starch (15 g/L, RS). After mixing well, the nutrient-rich medium was added to fermentation vessels (125 mL/vessel) autoclaved, equilibrated overnight under constant headspace flush with oxygen-free N_2_ (20psig, 5 mL/min) and adjusted to emulate the ascending colon (pH 5.5). Fecal samples collected from ten individuals participating in the parent study on day 0 and day 21 (HAB, n = 5; MRE, n = 5) were pooled in equal proportions and vessels inoculated with 10% (^v^/_v_) fecal slurry for final 2% inocula (^w^/_v_). Pooling promotes a highly diverse community and allows incorporation of low abundant, keystone species that may be limited using individualized samples to generate more generalizable insight, which is standard practice supported by Aguirre et al. [45]. Parallel control vessels were inoculated with cell-free phosphate buffer/glycerol. Aliquots were temporally removed from each vessel at 0, 5, 10, 24 and 48 h after exposure to RS2-supplemented medium and stored at -80 °C for DNA extraction and sequencing analysis. Fermentations were run in triplicate, at the same time, as experimental replicates.

### 16S rRNA gene amplicon sequencing

DNA from fecal samples was extracted using the QIAMP Power Fecal DNA Extraction Kit, QIAGEN, Inc. (Germantown, MD). DNA concentration (ng/uL) was quantified using Nanodrop One^c^ (ThermoFisher Scientific, Inc., Waltham, MA). Primers 515F (GTGYCAGCMGCCGCGGTAA) and 806R (GGACTACNVGGGTWTCTAAT) were used to amplify the V4 region of the 16S rRNA gene [46, 47]. No barcodes or adapters were included in the primers. Instead, the two-step, dual index PCR approach was used [48]. Nextera XT Index Kit v2 sets A and B (Cat. Nos. FC-131–2001 and FC-131–2002, Illumina, CA, USA) were used to index the 16S amplicons. The libraries were then normalized, pooled, and paired end sequenced (2 × 150 bp) on a NextSeq 500 (Illumina, CA, USA).

For downstream analyses, only the first forward sequencing reads were used. Primers were removed with the Python package cutadapt and only sequences 100 nucleotides or more were retained [49]. QIIME2 [50] with the DADA2 plugin (*qiime dada2 denoise-single*) was used to assign amplicon sequence variants (ASVs) with the added parameter of truncating sequences at position 124 [50, 51]. A phylogenetic tree was constructed with FastTree [52] by using the *qiime alignment maft*, *qiime alignment mask*, *qiime phylogeny fasttree*, and *qiime phylogeny midpoint-root* plugins, all with default parameters. Taxonomy was assigned using Greengenes (gg-13–8-99–515-806-nb-classifier). 2 257 ASVs were determined representing 35 254 964 sequence counts. The mean read abundance per sample was 542 384.06 and the mean read abundance per feature was 15 620.276.

### Whole-metagenome sequencing (WMS)

Sequencing libraries were prepared using the Nextera XT DNA Library Prep Kit (Cat. No. FC-131–1096, Illumina, CA, USA) and the Nextera XT Index Kit v2 set C (Cat. No. FC-131–2003, Illumina, CA, USA) according to the manufacturer’s protocol. Samples were normalized, pooled, and paired-end sequenced (2 × 150 bp) on a NextSeq500 (Illumina, CA, USA).

Metagenomic assembly and binning was completed with metaWRAP pipeline modules [53]. Default parameters were used unless noted. The module *metawrap read-qc* was used to quality filter reads for each sample. Paired-end reads for all samples were combined and co-assembled with the *metawrap assembly* module using MEGAHIT [54, 55]. Assembled contigs were binned with the *metawrap binning* module using MetaBAT2, MaxBin2, and CONCOCT programs [56–58]. Bins were consolidated and refined with the *metawrap bin_refinement* module with a minimum completion of 50% and maximum contamination of 10%. Bin abundances across samples were quantified with the *metawrap quant_bins* module which uses Salmon [59]. To improve assemblies, the refined bins were reassembled with the *metawrap reassemble_bins* module with a minimum completion of 50% and maximum contamination of 10%. Reassembled bins were functionally annotated with the *metawrap annotate* module which uses Prokka [60]. To search the metagenome assembly for CAZymes, the *run_dbcan.py* (https://github.com/linnabrown/run_dbcan) script was used. This script uses hidden Markov models (HMM) to search for CAZyme boundaries according to the dbCAN CAZyme domain HMM database [61, 62]. Finally, bin taxonomy was assigned according to The Genome Taxonomy Database (GTDB) [63]. First, the reassembled bins were converted into contig databases with Anvi’o (*anvi-script-reformat fasta*, *anvi-gen-contigs-database* programs) [64]. Single-copy core gene taxonomy search databases were setup with the *anvi-scg-databases* program and taxonomy was estimated using the *anvi-run-scg-taxonomy* and *anvi-estimate-genome-taxonomy* programs. For functional and taxonomic analyses independent of metagenome assembly, HUMAnN2 [11] and MetaPhlan2 [65] were used to annotate gene families/pathways and taxonomy, respectively.

We obtained a total number of raw reads of 245 357 634, including both paired reads; a total number of QC reads of 245 000 244; a mean raw reads per sample of 3 774 732; and a mean QC reads per sample of 3 769 234. The combination of both techniques (16S and WMS) can contribute to comprehending the differences within and between individuals/samples [66]. By employing 16S analysis, community composition changes were explored while with whole-metagenome sequencing, functional capacity of the community in terms of genes and pathways were examined. Both techniques can be used as a complement of each other as they provide powerful combined information.

### Data analysis

Custom R [67] scripts were used for analysis and visualization of 16S and whole genome data. For the beta diversity analysis, the weighted unifrac distance PCoA was employed only for the 16S data, and the Bray Curtis PCoA analysis was employed for the Whole Genome Sequencing and is presented in Supplementary information. For principal coordinates analysis in whole genome data (PCoA), the *vegan* package (https://github.com/vegandevs/vegan/) was used to compute Bray–Curtis distances between samples and this distance matrix was input into PCoA with the *labdsv* package (http://ecology.msu.montana.edu/labdsv/R). Experimental factor significance and proportion of variance explained was determined by PERMANOVA with the *adonis* function using the Bray–Curtis distance matrix [50]. For 16S data, QIIME2 software was used. Features that were present in less than half of the samples were removed. The data was baselined by subtracting feature abundance for each bioreactor vessel at time zero from feature abundances at subsequent time points to compensate for differences in pooled inocula due to volunteers. To determine importance and significance of the MRE-diet intervention (Diet*StudyDay*Fermentation Time interaction), a Linear Mixed Model (LMM) analysis was employed, which aimed to identify between-group/condition differences in trajectories of changes in relative abundance of important features over time. Raw sequencing reads and metagenome assembled data were deposited in the public database NCBI SRA, BioProject ID: PRJNA675102 (https://www.ncbi.nlm.nih.gov/search/all/?term=PRJNA675102).

Relative abundance data, after filtering, for the 127 organisms, 289 pathways, and 300 CAZymes were arcsine square root transformed, which is a commonly used approach for microbiome differential relative abundance testing, to normalize distributions [68–70] and analyzed using repeated measured ANOVA. Models included “Diet” (MRE and HAB) and “Study day” (0 and 21) as between-groups factors and “Fermentation time” (0, 5, 10, 24 and 48 h) as a within-subjects factor.

For features demonstrating a statistically significant diet*study day*fermentation time interaction (p < 0.05) relative to the MRE21 group, pairwise comparisons between groups were tested within each time point separately using ANOVA with Tukeys HSD. To assess whether significant differences were due to relative abundance at 0 h, features were subjected to analysis of covariance with fermentation time as the covariate and tested for equality of slopes between groups. To generate regression lines, arcsine transformed data were subjected to one-way analysis of covariance (ANCOVA, p < 0.05) with HAB_0, HAB_21, MRE_0, and MRE_21 as the groups, and using fermentation time as the covariate. To satisfy conditions for normality (via Shapiro–Wilk test), data for *Dorea* and *Enterobacteriaceae* were further log-transformed before performing the ANCOVA. Analysis was performed using SigmaStat 4.0 (Inpixion, Palo Alto, California). We used Signif. codes: 0 ‘***’ 0.001 ‘**’ 0.01 ‘*’ 0.05 ‘.’ 0.1 ‘’ 1; and BH: Benjamini–Hochberg for the adjusted p-values (method selected to control the FDR). A BH adjusted P-value < 0.05 was considered statistically significant.

## Supplementary Information

**Additional file 1.**

**Additional file 2.**

## Data Availability

Raw sequencing reads and metagenome assembled data were deposited in the public database NCBI SRA, BioProject ID: PRJNA675102 (https://www.ncbi.nlm.nih.gov/search/all/?term=PRJNA675102).

## Abbreviations

MRE: Meal Ready-to-Eat; HAB: Habitual Diet
RS2: Resistant Starch II
RS: Resistant Starch
PD: Faith’s Phylogenetic Diversity
MAGs: Metagenome Assembled Genomes
CAZymes: Carbohydrate-active Enzymes
WMS: Whole-metagenome sequencing
