# Supplementary material for: In vitro gut microbiome response to carbohydrate supplementation is acutely affected by a sudden change in diet
Source: BMC Microbiol. 2023 Jan 28;23:32. doi: 10.1186/s12866-023-02776-2 (PMC9883884; doi:10.1186/s12866-023-02776-2)
Supplement: Supplementary file 1 — Additional file 1. [file 12866_2023_2776_MOESM1_ESM.pdf]

Code/ 0040777 0000000 0000000 00000000000
14067154050 006670 5 ustar
00
Code/.Rhistory 0100777 0000000 0000000 00000000000
13545660462 010513 0 ustar
00
Code/Baseline_Humann2.R 0100777 0000000 0000000
00000021552 13506426013 012130 0 ustar 00
```
## load packages

library(ggplot2)

library(RColorBrewer)

library(vegan)

library(labdsv)


setwd("/Data1/NatickSeq/Natick_2018/Natick_WGS/")


# load metadata

MapFile =
read.table("/Data1/NatickSeq/Natick_2018/Natick_16S/QIIME2_analysis/Natick_MREferm_MappingFile
_2018.txt",

                  sep = "\t", comment.char = "", header = TRUE, stringsAsFactors = FALSE)

MREMap = MapFile[which(MapFile$Study == "MREferm"),]


Diet = c("Habitual", "MRE")

Date = c("Day0", "Day21")

TimesToAnalyze = c(5, 10, 24, 48)

Tmpt = c(0, 5, 10, 24, 48)

SampsToKeep = MREMap$X.SampleID[which(MREMap$Date %in% Date & MREMap$Time_Point.hrs.
%in% Tmpt)]

MREMapNoPool = MREMap[which(MREMap$X.SampleID %in% SampsToKeep),]


# Pathway analysis
```

```
PathAbundUnstrat =
read.table("./Humann2/Output/SplitPaths/MRE_humann2_pathabundances_cpm_unstratified.tsv",
                              sep = "\t", comment.char = "", header = TRUE, stringsAsFactors = FALSE, quote = "")
colnames(PathAbundUnstrat) = c("Pathway", MREMap$X.SampleID)
rownames(PathAbundUnstrat) = PathAbundUnstrat$Pathway
PathAbundUnstrat = PathAbundUnstrat[,-1]
PathAbundUnstratNoUns = PathAbundUnstrat[which(!(rownames(PathAbundUnstrat) %in%
c("UNMAPPED", "UNINTEGRATED"))),]
PathAbundUnstratNoUns = PathAbundUnstratNoUns[,which(colnames(PathAbundUnstratNoUns) %in%
MREMapNoPool$X.SampleID)]


Diets = character()
Dates = character()
Times = numeric()
Vessel = character()
DistFromBase = numeric()
for(i in 1:length(Diet)){
  for(j in 1:length(Date)){
    Vessels = unique(MREMapNoPool$Vessel[which(MREMapNoPool$Diet == Diet[i] &
MREMapNoPool$Date == Date[j])])
    for(k in 1:length(Vessels)){
      Samp0 = MREMapNoPool$X.SampleID[which(MREMapNoPool$Vessel == Vessels[k] &
MREMapNoPool$Time_Point.hrs. == 0)]
      for(l in 1:length(TimesToAnalyze)){
        mysamp = MREMapNoPool$X.SampleID[which(MREMapNoPool$Vessel == Vessels[k] &
                                          MREMapNoPool$Time_Point.hrs. == TimesToAnalyze[l])]
        print(paste(Diet[i], Date[j], Vessels[k], TimesToAnalyze[l], mysamp))
        Diets = c(Diets, Diet[i])
        Dates = c(Dates, Date[j])
        Vessel = c(Vessel, Vessels[k])
```

```
        Times = c(Times, TimesToAnalyze[l])
        DistFromBase = c(DistFromBase, Dist[Samp0, mysamp])
      }
    }
  }
}
TotDF = data.frame(Diets, Dates, Vessel, Times, DistFromBase, stringsAsFactors = FALSE)

pdf(paste("./Plots/Baseline/DistToBase_overtime_Humann2_pathway_", DistMetric, ".pdf", sep = ""))
p = ggplot(TotDF, aes(x = Times, y = DistFromBase, group = paste(Diets, Dates, Vessel),
                    color = paste(Diets, Dates))) +
  geom_line()
print(p)
dev.off()

# calculate baseline shannon diversity differences
Shannon = as.data.frame(diversity(t(PathAbundUnstratNoUns), index = "shannon"))
Diets = character()
Dates = character()
Times = numeric()
Vessel = character()
ShanDifFromBase = numeric()
for(i in 1:length(Diet)){
  for(j in 1:length(Date)){
    Vessels = unique(MREMapNoPool$Vessel[which(MREMapNoPool$Diet == Diet[i] &
MREMapNoPool$Date == Date[j])])
    for(k in 1:length(Vessels)){
      Samp0 = MREMapNoPool$X.SampleID[which(MREMapNoPool$Vessel == Vessels[k] &
MREMapNoPool$Time_Point.hrs. == 0)]
```

```
      for(l in 1:length(TimesToAnalyze)){
        mysamp = MREMapNoPool$X.SampleID[which(MREMapNoPool$Vessel == Vessels[k] &
                                          MREMapNoPool$Time_Point.hrs. == TimesToAnalyze[l])]
        print(paste(Diet[i], Date[j], Vessels[k], TimesToAnalyze[l], mysamp))
        Diets = c(Diets, Diet[i])
        Dates = c(Dates, Date[j])
        Vessel = c(Vessel, Vessels[k])
        Times = c(Times, TimesToAnalyze[l])
        ShanDifFromBase = c(ShanDifFromBase, Shannon[mysamp,] - Shannon[Samp0,])
      }
    }
  }
}

TotDF = data.frame(Diets, Dates, Vessel, Times, ShanDifFromBase, stringsAsFactors = FALSE)

pdf(paste("./Plots/Baseline/DistToBase_overtime_Humann2_pathway_", "Shannon", ".pdf", sep = ""))
p = ggplot(TotDF, aes(x = Times, y = ShanDifFromBase, group = paste(Diets, Dates, Vessel),
                  color = paste(Diets, Dates))) +
  geom_line()
print(p)
dev.off()

#############################################################################################
######################
# baseline the taxa table then filter for enriched taxa in MRE Day 21
mydf = t(PathAbundUnstratNoUns)
colnames(mydf) = gsub(":.*", "", colnames(mydf))
colnames(mydf) = gsub("-", ".", colnames(mydf))
```

```
Diets = character()

Dates = character()

Times = numeric()

Vessel = character()

DifFromBase = data.frame()

for(i in 1:length(Diet)){

  for(j in 1:length(Date)){

    Vessels = unique(MREMapNoPool$Vessel[which(MREMapNoPool$Diet == Diet[i] &
MREMapNoPool$Date == Date[j])])

    for(k in 1:length(Vessels)){

      Samp0 = MREMapNoPool$X.SampleID[which(MREMapNoPool$Vessel == Vessels[k] &
MREMapNoPool$Time_Point.hrs. == 0)]

      for(l in 1:length(TimesToAnalyze)){

        mysamp = MREMapNoPool$X.SampleID[which(MREMapNoPool$Vessel == Vessels[k] &

                                                MREMapNoPool$Time_Point.hrs. == TimesToAnalyze[l])]

        print(paste(Diet[i], Date[j], Vessels[k], TimesToAnalyze[l], mysamp))

        Diets = c(Diets, Diet[i])

        Dates = c(Dates, Date[j])

        Vessel = c(Vessel, Vessels[k])

        Times = c(Times, TimesToAnalyze[l])

        DifFromBase = rbind(DifFromBase, mydf[mysamp,] - mydf[Samp0,])

      }

    }

  }

}

colnames(DifFromBase) = colnames(mydf)

## take means

MeanDF = data.frame()

myDiet = character()
```

```
myDate = character()
myTime = numeric()
for(i in 1:length(Diet)){
  for(j in 1:length(Date)){
    for(k in 1:length(TimesToAnalyze)){
      MeanDF = rbind(MeanDF, colMeans(DifFromBase[which(Diets == Diet[i] &
                                                  Dates == Date[j] &
                                                  Times == TimesToAnalyze[k]),]))
      myDiet = c(myDiet, Diet[i])
      myDate = c(myDate, Date[j])
      myTime = c(myTime, TimesToAnalyze[k])
    }
  }
}
colnames(MeanDF) = colnames(mydf)
TotDF = data.frame(Diet = myDiet, Date = myDate, Time = myTime, MeanDF, stringsAsFactors = FALSE)

myFoundTaxUp = character()
myFoundTaxDown = character()
for(i in 1:length(TimesToAnalyze)){
  TimeDF = TotDF[which(myTime == TimesToAnalyze[i]),]
  KeepTaxaPos = colnames(TimeDF[,4:ncol(TimeDF)])[which(TimeDF[which(TimeDF$Diet == "MRE" &
TimeDF$Date == "Day21"),
                                                    4:ncol(TimeDF)] ==
                                                  apply(TimeDF[,4:ncol(TimeDF)], 2, max))]
  myFoundTaxUp = c(myFoundTaxUp, KeepTaxaPos)
  KeepTaxaNeg = colnames(TimeDF[,4:ncol(TimeDF)])[which(TimeDF[which(TimeDF$Diet == "MRE" &
TimeDF$Date == "Day21"),
                                                    4:ncol(TimeDF)] ==
```

```
                                    apply(TimeDF[,4:ncol(TimeDF)], 2, min))]
  myFoundTaxDown = c(myFoundTaxDown, KeepTaxaNeg)
}
UpDF = as.data.frame(table(myFoundTaxUp), stringsAsFactors = FALSE)
DownDF = as.data.frame(table(myFoundTaxDown), stringsAsFactors = FALSE)
TaxToKeep = unique(c(UpDF$myFoundTaxUp[which(UpDF$Freq >= 3)],
DownDF$myFoundTaxDown[which(DownDF$Freq >= 3)]))
TaxToKeep = gsub("^X", "", TaxToKeep)


## plot taxa for each vessel over time
for(h in 1:length(TaxToKeep)){
  myTaxa = as.data.frame(mydf[,which(colnames(mydf) == TaxToKeep[h])])
  Diets = character()
  Dates = character()
  Times = numeric()
  Vessel = character()
  TaxDifFromBase = numeric()
  for(i in 1:length(Diet)){
    for(j in 1:length(Date)){
      Vessels = unique(MREMapNoPool$Vessel[which(MREMapNoPool$Diet == Diet[i] &
MREMapNoPool$Date == Date[j])])
      for(k in 1:length(Vessels)){
        Samp0 = MREMapNoPool$X.SampleID[which(MREMapNoPool$Vessel == Vessels[k] &
MREMapNoPool$Time_Point.hrs. == 0)]
        for(l in 1:length(TimesToAnalyze)){
          mysamp = MREMapNoPool$X.SampleID[which(MREMapNoPool$Vessel == Vessels[k] &
                                    MREMapNoPool$Time_Point.hrs. == TimesToAnalyze[l])]
          print(paste(Diet[i], Date[j], Vessels[k], TimesToAnalyze[l], mysamp))
          Diets = c(Diets, Diet[i])
          Dates = c(Dates, Date[j])
```

```
        Vessel = c(Vessel, Vessels[k])
        Times = c(Times, TimesToAnalyze[l])
        TaxDifFromBase = c(TaxDifFromBase, myTaxa[mysamp,] - myTaxa[Samp0,])
      }
    }
  }
 }

 TotDF = data.frame(Diets, Dates, Vessel, Times, TaxDifFromBase, stringsAsFactors = FALSE)

 pdf(paste("./Plots/Baseline/Humann2_trends/DistToBase_overtime_", TaxToKeep[h], ".pdf", sep = ""))
 p = ggplot(TotDF, aes(x = Times, y = TaxDifFromBase, group = paste(Diets, Dates, Vessel),
                  color = paste(Diets, Dates))) +
  geom_line()
 print(p)
 dev.off()
}
```

Code/Baseline_MetaPhlan2.R 0100777 0000000 0000000
00000024533 13506422240 012553 0 ustar 00

```
## load packages
library(ggplot2)
library(RColorBrewer)
library(vegan)
library(labdsv)

setwd("/Data1/NatickSeq/Natick_2018/Natick_WGS/")

# load metadata
```

```
MapFile =
read.table("/Data1/NatickSeq/Natick_2018/Natick_16S/QIIME2_analysis/Natick_MREferm_MappingFile
_2018.txt",
                  sep = "\t", comment.char = "", header = TRUE, stringsAsFactors = FALSE)
MREMap = MapFile[which(MapFile$Study == "MREferm"),]


Diet = c("Habitual", "MRE")
Date = c("Day0", "Day21")
TimesToAnalyze = c(5, 10, 24, 48)
Tmpt = c(0, 5, 10, 24, 48)
SampsToKeep = MREMap$X.SampleID[which(MREMap$Date %in% Date & MREMap$Time_Point.hrs.
%in% Tmpt)]
MREMapNoPool = MREMap[which(MREMap$X.SampleID %in% SampsToKeep),]


# load MetaPhlan2 abundances
MetPhlan = read.csv("./Metaphlan2/MetaPhlan2_AllSamps_mergedAbund_species.csv",
                  header = TRUE, stringsAsFactors = FALSE)
rownames(MetPhlan) = MetPhlan$ID
MetPhlan = MetPhlan[,-1]
MetPhlanT = t(MetPhlan)
rownames(MetPhlanT) = gsub(".WGS", "", rownames(MetPhlanT))
MetPhlanTsub = MetPhlanT[which(rownames(MetPhlanT) %in% MREMapNoPool$X.SampleID),]
MetPhlanTsub = MetPhlanTsub[,which(apply(MetPhlanTsub, MARGIN = 2, function(x) any(x > 1)))]


Diets = character()
Dates = character()
Times = numeric()
Vessel = character()
DistFromBase = numeric()
for(i in 1:length(Diet)){
```

```
  for(j in 1:length(Date)){
    Vessels = unique(MREMapNoPool$Vessel[which(MREMapNoPool$Diet == Diet[i] &
MREMapNoPool$Date == Date[j])])
    for(k in 1:length(Vessels)){
      Samp0 = MREMapNoPool$X.SampleID[which(MREMapNoPool$Vessel == Vessels[k] &
MREMapNoPool$Time_Point.hrs. == 0)]
      for(l in 1:length(TimesToAnalyze)){
        mysamp = MREMapNoPool$X.SampleID[which(MREMapNoPool$Vessel == Vessels[k] &
                                          MREMapNoPool$Time_Point.hrs. == TimesToAnalyze[l])]
        print(paste(Diet[i], Date[j], Vessels[k], TimesToAnalyze[l], mysamp))
        Diets = c(Diets, Diet[i])
        Dates = c(Dates, Date[j])
        Vessel = c(Vessel, Vessels[k])
        Times = c(Times, TimesToAnalyze[l])
        DistFromBase = c(DistFromBase, Dist[Samp0, mysamp])
      }
    }
  }
}
TotDF = data.frame(Diets, Dates, Vessel, Times, DistFromBase, stringsAsFactors = FALSE)

pdf(paste("./Plots/Baseline/DistToBase_overtime_MetaPhlan2_class_", DistMetric, ".pdf", sep = ""))
p = ggplot(TotDF, aes(x = Times, y = DistFromBase, group = paste(Diets, Dates, Vessel),
                     color = paste(Diets, Dates))) +
  geom_line()
print(p)
dev.off()

# calculate baseline shannon diversity differences
```

```
Shannon = as.data.frame(diversity(MetPhlanTsub, index = "shannon"))

Diets = character()

Dates = character()

Times = numeric()

Vessel = character()

ShanDifFromBase = numeric()

for(i in 1:length(Diet)){

  for(j in 1:length(Date)){

    Vessels = unique(MREMapNoPool$Vessel[which(MREMapNoPool$Diet == Diet[i] &
MREMapNoPool$Date == Date[j])])

    for(k in 1:length(Vessels)){

      Samp0 = MREMapNoPool$X.SampleID[which(MREMapNoPool$Vessel == Vessels[k] &
MREMapNoPool$Time_Point.hrs. == 0)]

      for(l in 1:length(TimesToAnalyze)){

        mysamp = MREMapNoPool$X.SampleID[which(MREMapNoPool$Vessel == Vessels[k] &

                                               MREMapNoPool$Time_Point.hrs. == TimesToAnalyze[l])]

        print(paste(Diet[i], Date[j], Vessels[k], TimesToAnalyze[l], mysamp))

        Diets = c(Diets, Diet[i])

        Dates = c(Dates, Date[j])

        Vessel = c(Vessel, Vessels[k])

        Times = c(Times, TimesToAnalyze[l])

        ShanDifFromBase = c(ShanDifFromBase, Shannon[mysamp,] - Shannon[Samp0,])

      }

    }

  }

}


TotDF = data.frame(Diets, Dates, Vessel, Times, ShanDifFromBase, stringsAsFactors = FALSE)
```

```
pdf(paste("./Plots/Baseline/DistToBase_overtime_MetaPhlan2_genus_", "Shannon", ".pdf", sep = ""))
p = ggplot(TotDF, aes(x = Times, y = ShanDifFromBase, group = paste(Diets, Dates, Vessel),
                      color = paste(Diets, Dates))) +
  geom_line()
print(p)
dev.off()


## calculate baseline differences for each taxa
for(h in 1:ncol(MetPhlanTsub)){
  myTaxa = as.data.frame(MetPhlanTsub[,h])
  Diets = character()
  Dates = character()
  Times = numeric()
  Vessel = character()
  TaxDifFromBase = numeric()
  for(i in 1:length(Diet)){
    for(j in 1:length(Date)){
      Vessels = unique(MREMapNoPool$Vessel[which(MREMapNoPool$Diet == Diet[i] &
MREMapNoPool$Date == Date[j])])
      for(k in 1:length(Vessels)){
        Samp0 = MREMapNoPool$X.SampleID[which(MREMapNoPool$Vessel == Vessels[k] &
MREMapNoPool$Time_Point.hrs. == 0)]
        for(l in 1:length(TimesToAnalyze)){
          mysamp = MREMapNoPool$X.SampleID[which(MREMapNoPool$Vessel == Vessels[k] &
                                          MREMapNoPool$Time_Point.hrs. == TimesToAnalyze[l])]
          print(paste(Diet[i], Date[j], Vessels[k], TimesToAnalyze[l], mysamp))
          Diets = c(Diets, Diet[i])
          Dates = c(Dates, Date[j])
          Vessel = c(Vessel, Vessels[k])
```

```
        Times = c(Times, TimesToAnalyze[l])
        TaxDifFromBase = c(TaxDifFromBase, myTaxa[mysamp,] - myTaxa[Samp0,])
      }
    }
  }
 }

 TotDF = data.frame(Diets, Dates, Vessel, Times, TaxDifFromBase, stringsAsFactors = FALSE)

 pdf(paste("./Plots/Baseline/MetaPhlan2_genus/DistToBase_overtime_MetaPhlan2_genus_",
      colnames(MetPhlanTsub)[h], ".pdf", sep = ""))
 p = ggplot(TotDF, aes(x = Times, y = TaxDifFromBase, group = paste(Diets, Dates, Vessel),
           color = paste(Diets, Dates))) +
  geom_line()
 print(p)
 dev.off()
}

##########################################################################################
#######################
# baseline the taxa table then filter for enriched taxa in MRE Day 21
mydf = MetPhlanTsub
Diets = character()
Dates = character()
Times = numeric()
Vessel = character()
DifFromBase = data.frame()
for(i in 1:length(Diet)){
 for(j in 1:length(Date)){
```

```
   Vessels = unique(MREMapNoPool$Vessel[which(MREMapNoPool$Diet == Diet[i] &
MREMapNoPool$Date == Date[j])])
   for(k in 1:length(Vessels)){
     Samp0 = MREMapNoPool$X.SampleID[which(MREMapNoPool$Vessel == Vessels[k] &
MREMapNoPool$Time_Point.hrs. == 0)]
     for(l in 1:length(TimesToAnalyze)){
       mysamp = MREMapNoPool$X.SampleID[which(MREMapNoPool$Vessel == Vessels[k] &
                                        MREMapNoPool$Time_Point.hrs. == TimesToAnalyze[l])]
       print(paste(Diet[i], Date[j], Vessels[k], TimesToAnalyze[l], mysamp))
       Diets = c(Diets, Diet[i])
       Dates = c(Dates, Date[j])
       Vessel = c(Vessel, Vessels[k])
       Times = c(Times, TimesToAnalyze[l])
       DifFromBase = rbind(DifFromBase, mydf[mysamp,] - mydf[Samp0,])
     }
   }
  }
}
colnames(DifFromBase) = colnames(mydf)

## take means
MeanDF = data.frame()
myDiet = character()
myDate = character()
myTime = numeric()
for(i in 1:length(Diet)){
  for(j in 1:length(Date)){
    for(k in 1:length(TimesToAnalyze)){
      MeanDF = rbind(MeanDF, colMeans(DifFromBase[which(Diets == Diet[i] &
```

```
                                Dates == Date[j] &
                                Times == TimesToAnalyze[k]),]))
      myDiet = c(myDiet, Diet[i])
      myDate = c(myDate, Date[j])
      myTime = c(myTime, TimesToAnalyze[k])
    }
  }
}
colnames(MeanDF) = colnames(mydf)
TotDF = data.frame(Diet = myDiet, Date = myDate, Time = myTime, MeanDF, stringsAsFactors = FALSE)


myFoundTaxUp = character()
myFoundTaxDown = character()
for(i in 1:length(TimesToAnalyze)){
  TimeDF = TotDF[which(myTime == TimesToAnalyze[i]),]
  # for(j in 4:ncol(TimeDF)){
  #   if(abs(TimeDF[which(TimeDF$Diet == "MRE" & TimeDF$Date == "Day21"), j] -
  #      TimeDF[which(TimeDF$Diet == "MRE" & TimeDF$Date == "Day0"), j]) > 2){
  #     myFoundTax = c(myFoundTax, colnames(TimeDF)[j])
  #   }
  # }
  KeepTaxaPos = colnames(TimeDF[,4:ncol(TimeDF)])[which(TimeDF[which(TimeDF$Diet == "MRE" &
TimeDF$Date == "Day21"),
                                4:ncol(TimeDF)] ==
                              apply(TimeDF[,4:ncol(TimeDF)], 2, max))]
  myFoundTaxUp = c(myFoundTaxUp, KeepTaxaPos)
  KeepTaxaNeg = colnames(TimeDF[,4:ncol(TimeDF)])[which(TimeDF[which(TimeDF$Diet == "MRE" &
TimeDF$Date == "Day21"),
```

```
                                              4:ncol(TimeDF)] ==
                                          apply(TimeDF[,4:ncol(TimeDF)], 2, min))]
  myFoundTaxDown = c(myFoundTaxDown, KeepTaxaNeg)
}

UpDF = as.data.frame(table(myFoundTaxUp), stringsAsFactors = FALSE)
DownDF = as.data.frame(table(myFoundTaxDown), stringsAsFactors = FALSE)
TaxToKeep = unique(c(UpDF$myFoundTaxUp[which(UpDF$Freq >= 3)],
DownDF$myFoundTaxDown[which(DownDF$Freq >= 3)]))

## plot taxa for each vessel over time
for(h in 1:length(TaxToKeep)){
  myTaxa = as.data.frame(MetPhlanTsub[,which(colnames(MetPhlanTsub) == TaxToKeep[h])])
  Diets = character()
  Dates = character()
  Times = numeric()
  Vessel = character()
  TaxDifFromBase = numeric()
  for(i in 1:length(Diet)){
    for(j in 1:length(Date)){
      Vessels = unique(MREMapNoPool$Vessel[which(MREMapNoPool$Diet == Diet[i] &
MREMapNoPool$Date == Date[j])])
      for(k in 1:length(Vessels)){
        Samp0 = MREMapNoPool$X.SampleID[which(MREMapNoPool$Vessel == Vessels[k] &
MREMapNoPool$Time_Point.hrs. == 0)]
        for(l in 1:length(TimesToAnalyze)){
          mysamp = MREMapNoPool$X.SampleID[which(MREMapNoPool$Vessel == Vessels[k] &
                                      MREMapNoPool$Time_Point.hrs. == TimesToAnalyze[l])]
          print(paste(Diet[i], Date[j], Vessels[k], TimesToAnalyze[l], mysamp))
          Diets = c(Diets, Diet[i])
```

```
        Dates = c(Dates, Date[j])
        Vessel = c(Vessel, Vessels[k])
        Times = c(Times, TimesToAnalyze[l])
        TaxDifFromBase = c(TaxDifFromBase, myTaxa[mysamp,] - myTaxa[Samp0,])
      }
    }
  }
 }

 TotDF = data.frame(Diets, Dates, Vessel, Times, TaxDifFromBase, stringsAsFactors = FALSE)

 pdf(paste("./Plots/Baseline/MetaPhlan2_trends/DistToBase_overtime_", TaxToKeep[h], ".pdf", sep = 
""))
 p = ggplot(TotDF, aes(x = Times, y = TaxDifFromBase, group = paste(Diets, Dates, Vessel),
                 color = paste(Diets, Dates))) +
   geom_line()
 print(p)
 dev.off()
}
```

```
Code/ConsolidateTaxTabs.R                                    0100777 0000000 0000000 
00000003306 13577216556 012567  0                                                   ustar 
00
# consolidate gtdbk and megablast taxonomies into one table

setwd("/Data1/NatickSeq/Natick_2018/Natick_WGS/")

BlastTax = read.table("./metaWRAP/BIN_CLASSIFICATION/bin_taxonomy.tab", sep = "\t",
```

```
                    col.names = c("Bin", "MegaBlast_Tax"), quote = "", stringsAsFactors = FALSE)
BlastTax$Bin = gsub(".fa", "", BlastTax$Bin)

# consolidate all individual bin gtdbk tax tabs into one
Bins = BlastTax$Bin
mytax = character()
for(i in 1:length(Bins)){
  mytab = read.table(paste("./metaWRAP/AnvioBinTax/taxtabs/", Bins[i], ".gtdb.tax.txt", sep = ""),
                   sep = "\t", header = TRUE, quote = "", stringsAsFactors = FALSE)
  mytax = c(mytax, paste(mytab[1,4:10], collapse = ";"))
}

TotTab = data.frame(BlastTax, GTDBK_Tax = mytax, stringsAsFactors = FALSE)

TotTab = TotTab[order(as.numeric(unlist(lapply(strsplit(TotTab$Bin, "\\."), "[[", 2)))),]

write.table(TotTab, file = "./metaWRAP/AnvioBinTax/Bin_Taxonomy_megablast_gtdbk.txt", sep = "\t",
          row.names = FALSE, quote = FALSE)

### combine taxonomy into bin stat table
BinStats = read.table("./metaWRAP/BIN_REASSEMBLY/reassembled_bins.stats", sep = "\t",
                    quote = "", header = TRUE, stringsAsFactors = FALSE)
BinTax = read.table("./metaWRAP/AnvioBinTax/Bin_Taxonomy_megablast_gtdbk.txt", sep = "\t",
                  quote = "", header = TRUE, stringsAsFactors = FALSE)
TotBin = data.frame(BinStats[,-5], GTDBK_tax = BinTax$GTDBK_Tax[match(BinStats$bin, BinTax$Bin)],
                  stringsAsFactors = FALSE)
TotBinOrd = TotBin[order(TotBin$completeness, decreasing = TRUE),]
```

```
write.table(TotBinOrd, file = "./metaWRAP/AnvioBinTax/MAG_Stats_Tax.txt", sep = "\t",
          row.names = FALSE, quote = FALSE)
```

Code/metawrap_cazymes.R 0100777 0000000 0000000
00000036211 13563031511 012365 0 ustar 00

```
# parse CAZyme annotations


# create master table of contig abundances


library(heatmaply)
library(ggplot2)
library(htmlwidgets)
library(RColorBrewer)
library(vegan)
library(labdsv)
library(reshape2)
library(seqinr)


setwd("/Data1/NatickSeq/Natick_2018/Natick_WGS/")


# load metadata
MapFile =
read.table("/Data1/NatickSeq/Natick_2018/Natick_16S/QIIME2_analysis/Natick_MREferm_MappingFile
_2018.txt",
                  sep = "\t", comment.char = "", header = TRUE, stringsAsFactors = FALSE)
MREMap = MapFile[which(MapFile$Study == "MREferm"),]


Diet = c("Habitual", "MRE")
Date = c("Day0", "Day21")
TimesToAnalyze = c(5, 10, 24, 48)
```

```
Tmpt = c(0, 5, 10, 24, 48)

SampsToKeep = MREMap$X.SampleID[which(MREMap$Date %in% Date & MREMap$Time_Point.hrs.
%in% Tmpt)]

MREMapNoPool = MREMap[which(MREMap$X.SampleID %in% SampsToKeep),]


CazymeAnnot = read.table("./metaWRAP/CAZymeAnnot/HMMERout/hmmer.out", sep = "\t", header =
TRUE,

                         stringsAsFactors = FALSE)

CazymeAnnot$Gene.ID = gsub("_\\d*$", "", CazymeAnnot$Gene.ID)


UniqContigs = unique(CazymeAnnot$Gene.ID)


TotDF = data.frame()

Sample = character()

for(i in 1:nrow(MREMap)){

  SampMatch = paste(gsub("\\.", "-", MREMap$X.SampleID[i]), "_clean.quant.counts", sep = "")

  mytab = read.table(paste("./metaWRAP/QUANT_BINS/quant_files/", SampMatch, sep = ""), sep = "\t",

                     header = TRUE, stringsAsFactors = FALSE)

  mysubtab = data.frame(CazymeAnnot, Count = mytab$count[match(CazymeAnnot$Gene.ID,
mytab$transcript)],

                        stringsAsFactors = FALSE)

  TotDF = rbind(TotDF, mysubtab)

  Sample = c(Sample, rep(MREMap$X.SampleID[i], nrow(mysubtab)))

}

TotDF2 = data.frame(Sample, TotDF, stringsAsFactors = FALSE)

ContgsWCaz = TotDF2

write.csv(TotDF2, "./ParsedData/metawrap_MapCAZymesToContigs.csv", row.names = FALSE)


TotCast = dcast(TotDF2, Sample~HMM.Profile, value.var = "Count", fun.aggregate = sum)

write.csv(TotCast, "./ParsedData/metawrap_CAZymeAbund_BasedOnContigs.csv", row.names = FALSE)
```

```
CazAbun = read.csv("./ParsedData/metawrap_CAZymeAbund_BasedOnContigs.csv", header = TRUE,
                  row.names = 1)

# PCoA
Dist = vegdist(CazAbun, method = "bray")
mypco = pco(Dist, k = 10)
colnames(mypco$points) = paste(rep("PC", ncol(mypco$points)),
                            1:ncol(mypco$points), sep = "")
pc1 = round(mypco$eig[1]/sum(mypco$eig) * 100, digits = 1)
pc2 = round(mypco$eig[2]/sum(mypco$eig) * 100, digits = 1)
pc3 = round(mypco$eig[3]/sum(mypco$eig) * 100, digits = 1)
FullPCo = data.frame(MREMap, mypco$points, stringsAsFactors = FALSE)

pdf("./Plots/PCoA/metaWRAP_CAZymeAbunds_bray_PCoA.pdf")
p = ggplot(data = FullPCo, aes(x = PC1, y = PC2, colour = paste(Diet, Date), shape =
factor(Time_Point.hrs.))) +
  geom_point(size = 3) + theme_bw() +
  labs(x = paste("PC1 (", pc1, "%)", sep = ""),
       y = paste("PC2 (", pc2, "%)", sep = ""),
       title = paste(" Bray-Curtis", sep = "")) +
  scale_colour_brewer(palette="Set1")
print(p)
dev.off()

heatmaply(t(log2(CazAbun + 1)), dendrogram = "both",
         col_side_colors = data.frame(Diet = MREMap$Diet, Date = MREMap$Date, Tmpt =
MREMap$Time_Point.hrs.),
         colors = colorRampPalette(rev(brewer.pal(n = 7, name = "RdYlBu")))(100)) %>%
```

```
  layout(margin = list(l = 100, b = 120)) %>% print %>%

saveWidget(file=paste("/Data1/NatickSeq/Natick_2018/Natick_WGS/Plots/Heatmaps/metaWRAP_CAZyme_heatmap_log2plus1.html",
            sep = ""), selfcontained = FALSE)

mydf = CazAbun[which(rownames(CazAbun) %in% MREMapNoPool$X.SampleID),]
BrayAdon = adonis(mydf ~ Diet*Date*Time_Point.hrs., method = "bray",
         data = MREMapNoPool, strata = MREMapNoPool$Vessel, perm = 999)
write.csv(as.data.frame(BrayAdon$aov.tab), paste("./ParsedData/metawrap_CAZymeAbund_Bray_adonis.csv", sep = ""),
     row.names = TRUE)

###########################################################################################################
# baseline the taxa table then filter for enriched taxa in MRE Day 21
mydf = CazAbun
Diets = character()
Dates = character()
Times = numeric()
Vessel = character()
DifFromBase = data.frame()
for(i in 1:length(Diet)){
  for(j in 1:length(Date)){
    Vessels = unique(MREMapNoPool$Vessel[which(MREMapNoPool$Diet == Diet[i] & MREMapNoPool$Date == Date[j])])
    for(k in 1:length(Vessels)){
      Samp0 = MREMapNoPool$X.SampleID[which(MREMapNoPool$Vessel == Vessels[k] & MREMapNoPool$Time_Point.hrs. == 0)]
      for(l in 1:length(TimesToAnalyze)){
        mysamp = MREMapNoPool$X.SampleID[which(MREMapNoPool$Vessel == Vessels[k] &
```

```
                                        MREMapNoPool$Time_Point.hrs. == TimesToAnalyze[l])]
        print(paste(Diet[i], Date[j], Vessels[k], TimesToAnalyze[l], mysamp))
        Diets = c(Diets, Diet[i])
        Dates = c(Dates, Date[j])
        Vessel = c(Vessel, Vessels[k])
        Times = c(Times, TimesToAnalyze[l])
        DifFromBase = rbind(DifFromBase, mydf[mysamp,] - mydf[Samp0,])
      }
    }
  }
}
colnames(DifFromBase) = colnames(mydf)

## take means
MeanDF = data.frame()
myDiet = character()
myDate = character()
myTime = numeric()
for(i in 1:length(Diet)){
  for(j in 1:length(Date)){
    for(k in 1:length(TimesToAnalyze)){
      MeanDF = rbind(MeanDF, colMeans(DifFromBase[which(Diets == Diet[i] &
                                        Dates == Date[j] &
                                        Times == TimesToAnalyze[k]),]))
      myDiet = c(myDiet, Diet[i])
      myDate = c(myDate, Date[j])
      myTime = c(myTime, TimesToAnalyze[k])
    }
  }
```

```
}
colnames(MeanDF) = colnames(mydf)
TotDF = data.frame(Diet = myDiet, Date = myDate, Time = myTime, MeanDF, stringsAsFactors = FALSE)


myFoundTaxUp = character()
myFoundTaxDown = character()
for(i in 1:length(TimesToAnalyze)){
  TimeDF = TotDF[which(myTime == TimesToAnalyze[i]),]
  # for(j in 4:ncol(TimeDF)){
  #   if(abs(TimeDF[which(TimeDF$Diet == "MRE" & TimeDF$Date == "Day21"), j] -
  #     TimeDF[which(TimeDF$Diet == "MRE" & TimeDF$Date == "Day0"), j]) > 2){
  #     myFoundTax = c(myFoundTax, colnames(TimeDF)[j])
  #   }
  # }
  KeepTaxaPos = colnames(TimeDF[,4:ncol(TimeDF)])[which(TimeDF[which(TimeDF$Diet == "MRE" & 
TimeDF$Date == "Day21"),
                                                      4:ncol(TimeDF)] ==
                                                  apply(TimeDF[,4:ncol(TimeDF)], 2, max))]
  myFoundTaxUp = c(myFoundTaxUp, KeepTaxaPos)
  KeepTaxaNeg = colnames(TimeDF[,4:ncol(TimeDF)])[which(TimeDF[which(TimeDF$Diet == "MRE" & 
TimeDF$Date == "Day21"),
                                                      4:ncol(TimeDF)] ==
                                                  apply(TimeDF[,4:ncol(TimeDF)], 2, min))]
  myFoundTaxDown = c(myFoundTaxDown, KeepTaxaNeg)
}


UpDF = as.data.frame(table(myFoundTaxUp), stringsAsFactors = FALSE)
DownDF = as.data.frame(table(myFoundTaxDown), stringsAsFactors = FALSE)
TaxToKeep = unique(c(UpDF$myFoundTaxUp[which(UpDF$Freq >= 3)], 
DownDF$myFoundTaxDown[which(DownDF$Freq >= 3)]))
```

```
## plot taxa for each vessel over time
for(h in 1:length(TaxToKeep)){
  myTaxa = as.data.frame(CazAbun[,which(colnames(CazAbun) == TaxToKeep[h])])
  rownames(myTaxa) = rownames(CazAbun)
  Diets = character()
  Dates = character()
  Times = numeric()
  Vessel = character()
  TaxDifFromBase = numeric()
  for(i in 1:length(Diet)){
    for(j in 1:length(Date)){
      Vessels = unique(MREMapNoPool$Vessel[which(MREMapNoPool$Diet == Diet[i] &
MREMapNoPool$Date == Date[j])])
      for(k in 1:length(Vessels)){
        Samp0 = MREMapNoPool$X.SampleID[which(MREMapNoPool$Vessel == Vessels[k] &
MREMapNoPool$Time_Point.hrs. == 0)]
        for(l in 1:length(TimesToAnalyze)){
          mysamp = MREMapNoPool$X.SampleID[which(MREMapNoPool$Vessel == Vessels[k] &
                                                  MREMapNoPool$Time_Point.hrs. == TimesToAnalyze[l])]
          print(paste(Diet[i], Date[j], Vessels[k], TimesToAnalyze[l], mysamp))
          Diets = c(Diets, Diet[i])
          Dates = c(Dates, Date[j])
          Vessel = c(Vessel, Vessels[k])
          Times = c(Times, TimesToAnalyze[l])
          TaxDifFromBase = c(TaxDifFromBase, myTaxa[mysamp,] - myTaxa[Samp0,])
        }
      }
    }
```

```
  }

  TotDF = data.frame(Diets, Dates, Vessel, Times, TaxDifFromBase, stringsAsFactors = FALSE)

  pdf(paste("./Plots/Baseline/metawrap_trends/cazymes/DistToBase_overtime_", TaxToKeep[h], ".pdf", sep = ""))
  p = ggplot(TotDF, aes(x = Times, y = TaxDifFromBase, group = paste(Diets, Dates, Vessel),
                    color = paste(Diets, Dates))) +
    geom_line()
  print(p)
  dev.off()
}

##########################################################################################################
# map CAZyme annotations back to genomic bins

# load each bin fasta then map contigs to CAZyme table
Bins = list.files("./metaWRAP/BIN_REFINEMENT/metaWRAP_bins/")
myBins = character()
MapContigsToCaz = data.frame()
for(i in 1:length(Bins)){
  myfa = read.fasta(paste("./metaWRAP/BIN_REFINEMENT/metaWRAP_bins/", Bins[i], sep = ""), seqtype = "DNA")
  mycontigs = names(myfa)
  myCAZcontigs = CazymeAnnot[which(CazymeAnnot$Gene.ID %in% mycontigs),]
  myBins = c(myBins, rep(gsub(".fa", "", Bins[i]), nrow(myCAZcontigs)))
  MapContigsToCaz = rbind(MapContigsToCaz, myCAZcontigs)
}
BinCaz = data.frame(Bin = myBins, MapContigsToCaz, stringsAsFactors = FALSE)
```

```
write.csv(BinCaz, "./ParsedData/metawrap_MapCAZymeContigsToBins.csv", row.names = FALSE)

BinCast = dcast(BinCaz, Bin~HMM.Profile)
write.csv(BinCast, "./ParsedData/metawrap_NumCAZymesInBins.csv", row.names = FALSE)

##########################################################################################
#################
# group CAZymes by overall functional category
# AA, CBM, CE, cohesin, GH, GT, PL, SLH
CazAbun = read.csv("./ParsedData/metawrap_CAZymeAbund_BasedOnContigs.csv", header = TRUE,
         row.names = 1)

AA = rowSums(CazAbun[,grep("AA", colnames(CazAbun))])
CBM = rowSums(CazAbun[,grep("CBM", colnames(CazAbun))])
CE = rowSums(CazAbun[,grep("CE", colnames(CazAbun))])
GH = rowSums(CazAbun[,grep("GH", colnames(CazAbun))])
GT = rowSums(CazAbun[,grep("GT", colnames(CazAbun))])
PL = rowSums(CazAbun[,grep("PL", colnames(CazAbun))])

CatDF = data.frame(Samp = rownames(CazAbun), AA, CBM, CE, GH, GT, PL, stringsAsFactors = FALSE)
CatMelt = melt(CatDF, id.vars = "Samp")

CatMeltMeta = data.frame(CatMelt, MREMap[match(CatMelt$Samp, MREMap$X.SampleID),],
stringsAsFactors = FALSE)
CatMeltMeta$Date = factor(CatMeltMeta$Date, levels = c("Pool", "Day0", "Day21"))
CatMeltMeta$Time_Point.hrs. = factor(CatMeltMeta$Time_Point.hrs., levels = c("0", "5", "10", "24",
"48"))

p = ggplot(CatMeltMeta, aes(x = Samp, y = log10(value + 1), fill = variable)) +
```

```
  geom_bar(stat = "identity") + facet_grid(Diet~Date, scales = "free_x")
print(p)


###########################################################################################
#################
# jitter point plots for CAZyme abundances
TaxToKeep = c(TaxToKeep, colnames(CazAbun)[grep("GH13", colnames(CazAbun))])
for(h in 1:length(TaxToKeep)){
  myCazAbun = CazAbun[match(MREMapNoPool$X.SampleID, rownames(CazAbun)),
                      which(colnames(CazAbun) == TaxToKeep[h])]
  TotDF = data.frame(MREMapNoPool, CazAbund = myCazAbun, stringsAsFactors = FALSE)
  TotDF$Time_Point.hrs. = factor(TotDF$Time_Point.hrs., levels = c(0, 5, 10, 24, 48))


  pdf(paste("./Plots/Baseline/metawrap_trends/cazymes/CazAbund_overtime_", TaxToKeep[h], ".pdf",
sep = ""))
  p = ggplot(TotDF, aes(x = Time_Point.hrs., y = CazAbund, group = paste(Diet, Date, Vessel))) +
    geom_jitter(width = 0.1) +
    stat_summary(aes(group = paste(Diet, Date)), fun.y=mean, geom="line") +
    facet_wrap(~paste(Diet, Date), ncol = 4)
  print(p)
  dev.off()
  pdf(paste("./Plots/Baseline/metawrap_trends/cazymes/CazAbund_overtime_", TaxToKeep[h],
"_Time.pdf", sep = ""))
  p = ggplot(TotDF, aes(x = paste(Diet, Date), y = CazAbund, group = paste(Diet, Date, Vessel))) +
    geom_jitter(width = 0.1) +
    facet_wrap(~Time_Point.hrs., ncol = 5) +
    theme(axis.text.x = element_text(angle=45, hjust = 1))
  print(p)
  dev.off()
}
```

```
## further look at GH13 cazyme family which is starch degrading enzymes

GH13s = colnames(BinCast)[grep("GH13[_.]", colnames(BinCast))]

rownames(BinCast) = BinCast$Bin

BinCast = BinCast[,-1]

BinGH13 = BinCast[,grep("GH13[_.]", colnames(BinCast))]

BinGH13sub = BinGH13[which(rowSums(BinGH13) > 0),]



# subset CazymeAnnot table for GH13_*

# match contigs to bins, NAs are NotBinned

# add contig abundances


# add bin matches to contig w/ cazyme abundances

ContgsWCazBins = data.frame(ContgsWCaz, Bin = BinCaz$Bin[match(ContgsWCaz$Gene.ID,
BinCaz$Gene.ID)],

                           stringsAsFactors = FALSE)

ContgsWCazBins$Bin[is.na(ContgsWCazBins$Bin)] = "NotBinned"

ContgsWCazBins = ContgsWCazBins[which(ContgsWCazBins$Sample %in%
MREMapNoPool$X.SampleID),]


for(i in 1:length(GH13s)){

  mydf = ContgsWCazBins[which(ContgsWCazBins$HMM.Profile == GH13s[i]),]

  mycast = dcast(mydf, Sample~Bin, value.var = "Count", fun.aggregate = sum)

  rownames(mycast) = mycast$Sample

  if(length(unique(mydf$Bin)) >= 2){

    mycast = mycast[,-1]

    TotAbun = rowSums(mycast)

    if(ncol(mycast) > 10){
```

```
    mycast = mycast[,order(colSums(mycast), decreasing = TRUE)[1:10]]
   }
   mycast = data.frame(Sample = rownames(mycast), Total = TotAbun, mycast, stringsAsFactors = FALSE)
  }else{
   mycast = data.frame(mycast, Total = mycast[,2], stringsAsFactors = FALSE)
  }
  mymelt = melt(mycast, id.vars = "Sample")
  TotDF = data.frame(mymelt, MREMapNoPool[match(mymelt$Sample, MREMapNoPool$X.SampleID),],
                 stringsAsFactors = FALSE)
  TotDF$variable = factor(TotDF$variable, levels = colnames(mycast)[2:ncol(mycast)])

  pdf(paste("./Plots/Baseline/metawrap_trends/cazymes/GH13/", GH13s[i],
"_Top10BinAbundOverTime.pdf", sep = ""))
  p = ggplot(TotDF, aes(x = factor(Time_Point.hrs.), y = log10(value + 1), fill = variable)) +
   geom_bar(stat = "summary", fun.y = "mean", position = "dodge") +
   scale_fill_brewer(palette="Set3", name = "Bin") + facet_grid(Diet~Date) +
   labs(x = "Tmpt", y = "log10(CPM + 1)")
  print(p)
  dev.off()
}
```

Code/Plot_HUMAnN2.R 0100777 0000000 0000000
00000040130 13504744240 011120 0 ustar
00

```
##### HUMAnN2 results

# HUMAnN2 uses nucleotide mapping and translated search to provide organism specific gene and pathway abundances

# gene IDs from UniRef90

# pathways from MetaCyc

#### outputs:
```

```
#### genefamilies.tsv: abundances of each gene family in the community in RPK (reads per kilobase)

# broken down by organism contribution

# total RPK = UNMAPPED + unknown + all UniRef IDs, prior to organism specific breakdown

# can be normalized to CPM (copies per million)

#### pathabundance.tsv: abundances of each pathway, in RPK, broken down by organism

#### pathcoverage.tsv: coverage of each pathwy, probability that it is provided as a unit to the metagenome either within

# one or more particular organisms or across one or more organisms in aggregate

# should be considered probabilistic pres/abs call for each pathway and not summed

#### to normalize RPKs to CPMs

# use humann2_renorm_table --input file.tsv --ouput outfile-cpm.tsv --units cpm --update-snames

# unstratified outputs should summ to one million

#### can also regroup gene families by EC categories: humann2_regroup_table

#### join sample tables together

# humann2_join_tables -i DirectoryWithAllTabs -o AllSamp_genefamilies.tsv --file_name genefamilies

# then renorm table

# then split to stratified and unstratified humann2_split_stratified_table

#### to test for differences in pathways or genes btw categorical variable use Kruskal-Wallis H-test


## load packages

library(heatmaply)

library(ggplot2)

library(htmlwidgets)

library(RColorBrewer)

library(vegan)

library(labdsv)

library(reshape2)


setwd("/Data1/NatickSeq/Natick_2018/Natick_WGS/")
```

```
# load metadata

MapFile =
read.table("/Data1/NatickSeq/Natick_2018/Natick_16S/QIIME2_analysis/Natick_MREferm_MappingFile
_2018.txt",

        sep = "\t", comment.char = "", header = TRUE, stringsAsFactors = FALSE)

MREMap = MapFile[which(MapFile$Study == "MREferm"),]


Date = c("Day0", "Day21")

Tmpt = c(0, 5, 10, 24, 48)

SampsToKeep = MREMap$X.SampleID[which(MREMap$Date %in% Date & MREMap$Time_Point.hrs.
%in% Tmpt)]

ASVtab.min.nochlormit.rare.nozero =
ASVtab.min.nochlormit.rare.nozero[which(rownames(ASVtab.min.nochlormit.rare.nozero) %in%
SampsToKeep),]

MREMapNoPool = MREMap[which(MREMap$X.SampleID %in% SampsToKeep),]


# Pathway analysis

PathAbundUnstrat =
read.table("./Humann2/Output/SplitPaths/MRE_humann2_pathabundances_cpm_unstratified.tsv",

          sep = "\t", comment.char = "", header = TRUE, stringsAsFactors = FALSE, quote = "")

colnames(PathAbundUnstrat) = c("Pathway", MREMap$X.SampleID)

rownames(PathAbundUnstrat) = PathAbundUnstrat$Pathway

PathAbundUnstrat = PathAbundUnstrat[,-1]

PathAbundUnstratNoUns = PathAbundUnstrat[which(!(rownames(PathAbundUnstrat) %in%
c("UNMAPPED", "UNINTEGRATED"))),]

PathAbundUnstratNoUns = PathAbundUnstratNoUns[,which(colnames(PathAbundUnstratNoUns) %in%
MREMapNoPool$X.SampleID)]


PathAbundStrat =
read.table("./Humann2/Output/SplitPaths/MRE_humann2_pathabundances_cpm_stratified.tsv",

          sep = "\t", comment.char = "", header = TRUE, stringsAsFactors = FALSE, quote = "")
```

```
colnames(PathAbundStrat) = c("Pathway", MREMap$X.SampleID)

rownames(PathAbundStrat) = PathAbundStrat$Pathway

PathAbundStrat = PathAbundStrat[,-1]


heatmaply(log10(PathAbundUnstratNoUns + 1), dendrogram = "both",

        col_side_colors = data.frame(Diet = MREMap$Diet, Date = MREMap$Date, Tmpt = 
MREMap$Time_Point.hrs.),

        colors = colorRampPalette(rev(brewer.pal(n = 7, name = "RdYlBu")))(100)) %>%

  layout(margin = list(l = 100, b = 120)) %>% print %>%


saveWidget(file="/Data1/NatickSeq/Natick_2018/Natick_WGS/Plots/Heatmaps/Humann2_Pathways_h
eatmap_cpm.html",

           selfcontained = FALSE)


Dist = vegdist(t(PathAbundUnstratNoUns), method = "bray")

mypco = pco(Dist, k = 10)

colnames(mypco$points) = paste(rep("PC", ncol(mypco$points)),

                        1:ncol(mypco$points), sep = "")

pc1 = round(mypco$eig[1]/sum(mypco$eig) * 100, digits = 1)

pc2 = round(mypco$eig[2]/sum(mypco$eig) * 100, digits = 1)

pc3 = round(mypco$eig[3]/sum(mypco$eig) * 100, digits = 1)

FullPCo = data.frame(MREMapNoPool, mypco$points, stringsAsFactors = FALSE)


pdf("./Plots/PCoA/Humann2_Pathways_BrayCurtis_PCoA.pdf")

p = ggplot(data = FullPCo, aes(x = PC1, y = PC2, colour = paste(Diet, Date), shape = 
factor(Time_Point.hrs.))) +

  geom_point(size = 3) + theme_bw() +

  labs(x = paste("PC1 (", pc1, "%)", sep = ""),

      y = paste("PC2 (", pc2, "%)", sep = ""),

      title = "Pathways Bray-Curtis") +
```

```
  scale_colour_brewer(palette="Set1")
print(p)
dev.off()


mybray = adonis(t(PathAbundUnstratNoUns) ~ Diet*Date*Time_Point.hrs., method = "bray",
              data = MREMapNoPool, strata = MREMapNoPool$Vessel, perm = 999)
write.csv(as.data.frame(mybray$aov.tab), "./ParsedData/Humann2_Bray_Pathways_adonis.csv",
row.names = TRUE)
JaccDist = vegdist(t(PathAbundUnstratNoUns), method = "jaccard", binary = TRUE)
myjacc = adonis(JaccDist ~ Diet*Date*Time_Point.hrs.,
              data = MREMapNoPool, strata = MREMapNoPool$Vessel, perm = 999)
write.csv(as.data.frame(myjacc$aov.tab), "./ParsedData/Humann2_Jaccard_Pathways_adonis.csv",
row.names = TRUE)



# stat tests
# overall MRE vs Habitual diet
PathAbundUnstratT = t(PathAbundUnstrat)
PathAbundUnstratTMeta = data.frame(PathAbundUnstratT, MREMap, stringsAsFactors = FALSE)
Pval = numeric()
log2FoldChange = numeric()
MRE_mean = numeric()
Habitual_mean = numeric()
for(i in 1:ncol(PathAbundUnstratT)){
  WilcoxTest = wilcox.test(PathAbundUnstratTMeta[,i]~PathAbundUnstratTMeta$Diet)
  Pval = c(Pval, WilcoxTest$p.value)
  Mmean = mean(PathAbundUnstratTMeta[which(PathAbundUnstratTMeta$Diet == "MRE"),i])
  Hmean = mean(PathAbundUnstratTMeta[which(PathAbundUnstratTMeta$Diet == "Habitual"),i])
  log2FoldChange = c(log2FoldChange, log2(Mmean/Hmean))
```

```
  MRE_mean = c(MRE_mean, Mmean)
  Habitual_mean = c(Habitual_mean, Hmean)
}

TestDF = data.frame(Pathway = rownames(PathAbundUnstrat), log2FoldChange, MRE_mean,
                    Habitual_mean, Pval, stringsAsFactors = FALSE)
TestDF05 = TestDF[which(TestDF$Pval < 0.05),]
TestDF05Ord = TestDF05[order(abs(TestDF05$log2FoldChange), decreasing = TRUE),]
write.csv(TestDF05Ord, "./ParsedData/Humann2_Pathways_WilcoxTest_MREvsHab_AllSamps_05.csv",
          row.names = FALSE)

# stat tests for each tmpt
Tmpts = c(0, 5, 10, 24, 48)
for(h in 1:length(Tmpts)){
  PathSub = PathAbundUnstratTMeta[which(PathAbundUnstratTMeta$Time_Point.hrs. == Tmpts[h]),]
  Pval = numeric()
  log2FoldChange = numeric()
  MRE_mean = numeric()
  Habitual_mean = numeric()
  for(i in 1:ncol(PathAbundUnstratT)){
    WilcoxTest = wilcox.test(PathSub[,i]~PathSub$Diet)
    Pval = c(Pval, WilcoxTest$p.value)
    Mmean = mean(PathSub[which(PathSub$Diet == "MRE"),i])
    Hmean = mean(PathSub[which(PathSub$Diet == "Habitual"),i])
    log2FoldChange = c(log2FoldChange, log2(Mmean/Hmean))
    MRE_mean = c(MRE_mean, Mmean)
    Habitual_mean = c(Habitual_mean, Hmean)
  }
  TestDF = data.frame(Pathway = rownames(PathAbundUnstrat), log2FoldChange, MRE_mean,
```

```
                 Habitual_mean, Pval, stringsAsFactors = FALSE)
  TestDF05 = TestDF[which(TestDF$Pval < 0.05),]
  TestDF05Ord = TestDF05[order(abs(TestDF05$log2FoldChange), decreasing = TRUE),]
  write.csv(TestDF05Ord, paste("./ParsedData/Humann2_Pathways_WilcoxTest_MREvsHab_Tmpt_",
Tmpts[h],
                       "_Samps_05.csv", sep = ""), row.names = FALSE)
}



# Gene family analysis
GeneFamUnstrat =
read.table("./Humann2/Output/SplitGenes/MRE_humann2_genefamilies_cpm_unstratified.tsv",
                       sep = "\t", comment.char = "", header = TRUE, stringsAsFactors = FALSE, quote = "")
colnames(GeneFamUnstrat) = c("GeneFamily", MREMap$X.SampleID)
rownames(GeneFamUnstrat) = GeneFamUnstrat$GeneFamily
GeneFamUnstrat = GeneFamUnstrat[,-1]
GeneFamUnstratNoUns = GeneFamUnstrat[which(!(rownames(GeneFamUnstrat) %in% c("UNMAPPED",
"UniRef90_unknown"))),]
GeneFamUnstratNoUns = GeneFamUnstratNoUns[,which(colnames(GeneFamUnstratNoUns) %in%
MREMapNoPool$X.SampleID)]

Dist = vegdist(t(GeneFamUnstratNoUns), method = "bray")
mypco = pco(Dist, k = 10)
colnames(mypco$points) = paste(rep("PC", ncol(mypco$points)),
                       1:ncol(mypco$points), sep = "")
pc1 = round(mypco$eig[1]/sum(mypco$eig) * 100, digits = 1)
pc2 = round(mypco$eig[2]/sum(mypco$eig) * 100, digits = 1)
pc3 = round(mypco$eig[3]/sum(mypco$eig) * 100, digits = 1)
FullPCo = data.frame(MREMapNoPool, mypco$points, stringsAsFactors = FALSE)
```

```
pdf("./Plots/PCoA/Humann2_GeneFams_BrayCurtis_PCoA.pdf")

p = ggplot(data = FullPCo, aes(x = PC1, y = PC2, colour = paste(Diet, Date), shape =
factor(Time_Point.hrs.))) +

  geom_point(size = 3) + theme_bw() +

  labs(x = paste("PC1 (", pc1, "%)", sep = ""),

       y = paste("PC2 (", pc2, "%)", sep = ""),

       title = "Gene families Bray-Curtis") +

  scale_colour_brewer(palette="Set1")

print(p)

dev.off()


adonis(t(GeneFamUnstratNoUns) ~ Diet*Date*Time_Point.hrs., method = "bray",

       data = MREMapNoPool, strata = MREMapNoPool$Vessel, perm = 999)



mybray = adonis(t(GeneFamUnstratNoUns) ~ Diet*Date*Time_Point.hrs., method = "bray",

              data = MREMapNoPool, strata = MREMapNoPool$Vessel, perm = 999)

write.csv(as.data.frame(mybray$aov.tab), "./ParsedData/Humann2_Bray_GeneFams_adonis.csv",
row.names = TRUE)



#################################################################

# create meta/quant data table for HUMAnN2 stat analysis scripts

PathAll = read.table("./Humann2/Output/MRE_humann2_pathabundances_cpm.tsv", sep = "\t",

                  comment.char = "", quote = "", stringsAsFactors = FALSE, row.names = 1, header = TRUE)

colnames(PathAll) = MREMap$X.SampleID

MetaAll = MREMap

rownames(MetaAll) = MREMap$X.SampleID

MetaAll = MetaAll[,-1]
```

```
MetaAllT = data.frame(t(MetaAll), stringsAsFactors = FALSE)

OutPcl = rbind(MetaAllT, PathAll)

write.table(OutPcl, "./Humann2/Output/MRE_humann2_pathabundances_cpm_PlusMeta.pcl",

        sep = "\t", col.names = FALSE, row.names = TRUE, quote = FALSE)


GeneAll = read.table("./Humann2/Output/MRE_humann2_genefamilies_cpm.tsv", sep = "\t",

            comment.char = "", quote = "", stringsAsFactors = FALSE, row.names = 1, header = TRUE)

colnames(GeneAll) = MREMap$X.SampleID

OutPcl = rbind(MetaAllT, GeneAll)

write.table(OutPcl, "./Humann2/Output/MRE_humann2_genefamilies_cpm_PlusMeta.pcl",

        sep = "\t", col.names = FALSE, row.names = TRUE, quote = FALSE)

# ran Kruskal-Wallis tests with humann2 script

#humann2_associate --input MRE_humann2_pathabundances_cpm_PlusMeta.pcl --last-metadatum
Description --focal-metadatum Diet --focal-type categorical --output
MRE_humann2_pathabundances_cpm_KruskalWall_Diet.tsv

#humann2_associate --input MRE_humann2_genefamilies_cpm_PlusMeta.pcl --last-metadatum
Description --focal-metadatum Diet --focal-type categorical --output
MRE_humann2_genefamilies_cpm_KruskalWall_Diet.tsv

######################################################################

# plot pathways of interest

PathwayOfInt = "PWY-6737"

PathSub = PathAll[grep(PathwayOfInt, rownames(PathAll)),]

# take top 12 species

PathSub2 = PathSub[order(rowSums(PathSub), decreasing = TRUE)[1:12],]

rownames(PathSub2) = gsub("PWY-6737: ", "", rownames(PathSub2))

rownames(PathSub2) = gsub("starch degradation V\\|", "", rownames(PathSub2))

rownames(PathSub2) = gsub("g__.*s__", "", rownames(PathSub2))


PathSub3 = t(PathSub2)

PathMelt = melt(PathSub3)
```

```
PathMelt = data.frame(PathMelt, MREMap[match(PathMelt$Var1, MREMap$X.SampleID),], stringsAsFactors = FALSE)

PathMelt$Var2 = factor(PathMelt$Var2, levels = rownames(PathSub2))

Diet = c("MRE", "Habitual")

Date = c("Day0", "Day21")

Time = c(0, 24)

for(i in 1:length(Diet)){

  mydf = PathMelt[which(PathMelt$Diet == Diet[i] & PathMelt$Date %in% Date &

                        PathMelt$Time_Point.hrs. %in% Time),]

  pdf(paste("./Plots/HUMAnN2_PathwayBarplot_", PathwayOfInt, "_Top10SpeciesCPM_", Diet[i], "_0and24h.pdf", sep = ""))

  p = ggplot(mydf, aes(x = factor(Time_Point.hrs.), y = log10(value + 1), fill = Var2)) +

    geom_bar(stat = "identity", position = "dodge") +

    scale_fill_brewer(palette="Set3", name = "Species") + facet_wrap(~Date, nrow = 3, scales = "free_x") +

    labs(x = "Tmpt", y = "log10(CPM + 1)", title = Diet[i])

  print(p)

  dev.off()

}

#### compare HAB 0 and 21 days, compare MRE 0 and 21 days, then compare/contrast results

PathAbundUnstratT = t(PathAbundUnstrat)

PathAbundUnstratTMeta = data.frame(PathAbundUnstratT, MREMap, stringsAsFactors = FALSE)

TmptCompare = list(0, 5, 10, 24, 48, c(5, 10, 24))

for(h in 1:length(TmptCompare)){
```

```
  TmptTab = PathAbundUnstratTMeta[which(PathAbundUnstratTMeta$Time_Point.hrs. %in%
TmptCompare[[h]]),]

  HABTab = TmptTab[which(TmptTab$Diet == "Habitual" & TmptTab$Date %in% c("Day0", "Day21")),]

  MRETab = TmptTab[which(TmptTab$Diet == "MRE" & TmptTab$Date %in% c("Day0", "Day21")),]

  # compare HAB btw days 0 and 21

  Pval = numeric()

  log2FoldChange = numeric()

  Day0_mean = numeric()

  Day21_mean = numeric()

  for(i in 1:ncol(PathAbundUnstratT)){

    WilcoxTest = wilcox.test(HABTab[,i]~HABTab$Date)

    Pval = c(Pval, WilcoxTest$p.value)

    Mean0 = mean(HABTab[which(HABTab$Date == "Day0"),i])

    Mean21 = mean(HABTab[which(HABTab$Date == "Day21"),i])

    log2FoldChange = c(log2FoldChange, log2(Mean21/Mean0))

    Day0_mean = c(Day0_mean, Mean0)

    Day21_mean = c(Day21_mean, Mean21)

  }

  HabDF = data.frame(Pathway = colnames(PathAbundUnstratT), log2FoldChange, Day0_mean,

                  Day21_mean, Pval, stringsAsFactors = FALSE)

  HabDF05 = HabDF[which(HabDF$Pval < 0.05),]

  HabDF05Ord = HabDF05[order(abs(HabDF05$log2FoldChange), decreasing = TRUE),]

  if(nrow(HabDF05Ord) > 0){

    write.csv(HabDF05Ord,
paste("./ParsedData/Humann2_Pathway_WilcoxTest_HAB_Day0vs21_Tmpt_",

                          paste(TmptCompare[[h]], collapse = "_"),

                          "_Samps_05.csv", sep = ""), row.names = FALSE)

  }
```

```
  # compare MRE btw days 0 and 21
  Pval = numeric()
  log2FoldChange = numeric()
  Day0_mean = numeric()
  Day21_mean = numeric()
  for(i in 1:ncol(PathAbundUnstratT)){
    WilcoxTest = wilcox.test(MRETab[,i]~MRETab$Date)
    Pval = c(Pval, WilcoxTest$p.value)
    Mean0 = mean(MRETab[which(MRETab$Date == "Day0"),i])
    Mean21 = mean(MRETab[which(MRETab$Date == "Day21"),i])
    log2FoldChange = c(log2FoldChange, log2(Mean21/Mean0))
    Day0_mean = c(Day0_mean, Mean0)
    Day21_mean = c(Day21_mean, Mean21)
  }
  MreDF = data.frame(Pathway = colnames(PathAbundUnstratT), log2FoldChange, Day0_mean,
                 Day21_mean, Pval, stringsAsFactors = FALSE)
  MreDF05 = MreDF[which(MreDF$Pval < 0.05),]
  MreDF05Ord = MreDF05[order(abs(MreDF05$log2FoldChange), decreasing = TRUE),]
  if(nrow(MreDF05Ord) > 0){
    write.csv(MreDF05Ord,
paste("./ParsedData/Humann2_Pathway_WilcoxTest_MRE_Day0vs21_Tmpt_",
                           paste(TmptCompare[[h]], collapse = "_"),
                           "_Samps_05.csv", sep = ""), row.names = FALSE)
  }
  print(paste(nrow(HabDF05Ord), nrow(MreDF05Ord)))
}

Code/Plot_MetaPhlan2.R                                   0100777 0000000 0000000
00000026635 13505417725 011767  0                                              ustar
```

00

```
# plot MetaPhlan2 taxonomic abundance results

## load packages
library(heatmaply)
library(ggplot2)
library(htmlwidgets)
library(RColorBrewer)
library(vegan)
library(labdsv)

setwd("/Data1/NatickSeq/Natick_2018/Natick_WGS/")

# load metadata
MapFile =
read.table("/Data1/NatickSeq/Natick_2018/Natick_16S/QIIME2_analysis/Natick_MREferm_MappingFile
_2018.txt",
           sep = "\t", comment.char = "", header = TRUE, stringsAsFactors = FALSE)
MREMap = MapFile[which(MapFile$Study == "MREferm"),]

Date = c("Day0", "Day21")
Tmpt = c(0, 5, 10, 24, 48)
SampsToKeep = MREMap$X.SampleID[which(MREMap$Date %in% Date & MREMap$Time_Point.hrs.
%in% Tmpt)]
MREMapNoPool = MREMap[which(MREMap$X.SampleID %in% SampsToKeep),]

Levels = c("kingdom", "phylum", "order", "class", "family", "genus", "species")
for(i in 1:length(Levels)){
  # load MetaPhlan2 abundances
```

```
  MetPhlan = read.csv(paste("./Metaphlan2/MetaPhlan2_AllSamps_mergedAbund_", Levels[i], ".csv",
sep = ""),
                      header = TRUE, stringsAsFactors = FALSE)
  rownames(MetPhlan) = MetPhlan$ID
  MetPhlan = MetPhlan[,-1]
  MetPhlanT = t(MetPhlan)
  rownames(MetPhlanT) = gsub(".WGS", "", rownames(MetPhlanT))
  MetPhlanTsub = MetPhlanT[which(rownames(MetPhlanT) %in% MREMapNoPool$X.SampleID),]
  MetPhlanTsub = MetPhlanTsub[,which(apply(MetPhlanTsub, MARGIN = 2, function(x) any(x > 1)))]

  # heatmaply(t(MetPhlanTsub/100), dendrogram = "both",
  #           col_side_colors = data.frame(Diet = MREMap$Diet, Date = MREMap$Date, Tmpt =
MREMap$Time_Point.hrs.),
  #           colors = colorRampPalette(rev(brewer.pal(n = 7, name = "RdYlBu")))(100)) %>%
  #   layout(margin = list(l = 100, b = 120)) %>% print %>%
  #
saveWidget(file=paste("/Data1/NatickSeq/Natick_2018/Natick_WGS/Plots/Heatmaps/Metaphlan_heat
map_",
  #                       Levels[i], "_RelAbund.html", sep = ""), selfcontained = FALSE)

  Dist = vegdist(MetPhlanTsub, method = "bray")
  mypco = pco(Dist, k = 10)
  colnames(mypco$points) = paste(rep("PC", ncol(mypco$points)),
                                 1:ncol(mypco$points), sep = "")
  pc1 = round(mypco$eig[1]/sum(mypco$eig) * 100, digits = 1)
  pc2 = round(mypco$eig[2]/sum(mypco$eig) * 100, digits = 1)
  pc3 = round(mypco$eig[3]/sum(mypco$eig) * 100, digits = 1)
  FullPCo = data.frame(MREMapNoPool, mypco$points, stringsAsFactors = FALSE)

  # pdf(paste("./Plots/PCoA/MetaphlanTax_", Levels[i], "_BrayCurtis_PCoA.pdf", sep = ""))
```

```
  p = ggplot(data = FullPCo, aes(x = PC1, y = PC2, colour = paste(Diet, Date), shape =
factor(Time_Point.hrs.))) +
    geom_point(size = 3) + theme_bw() +
    labs(x = paste("PC1 (", pc1, "%)", sep = ""),
         y = paste("PC2 (", pc2, "%)", sep = ""),
         title = paste(Levels[i], " Bray-Curtis", sep = "")) +
    scale_colour_brewer(palette="Set1")
  print(p)
  # dev.off()

  print(Levels[i])
  BrayAdon = adonis(MetPhlanTsub ~ Diet*Date*Time_Point.hrs., method = "bray",
                data = MREMapNoPool, strata = MREMapNoPool$Vessel, perm = 999)
  write.csv(as.data.frame(BrayAdon$aov.tab), paste("./ParsedData/Metaphlan2_Bray_", Levels[i],
"_adonis.csv", sep = ""),
          row.names = TRUE)



### stat test for differential species abundances
# stat tests
# overall MRE vs Habitual diet
MetPhlan = read.csv("./Metaphlan2/MetaPhlan2_AllSamps_mergedAbund_species.csv",
                header = TRUE, stringsAsFactors = FALSE)
rownames(MetPhlan) = MetPhlan$ID
MetPhlan = MetPhlan[,-1]
MetPhlanT = t(MetPhlan)
rownames(MetPhlanT) = gsub(".WGS", "", rownames(MetPhlanT))
MetPhlanTmeta = data.frame(MetPhlanT[which(rownames(MetPhlanT) %in% MREMap$X.SampleID),],
                     MREMap, stringsAsFactors = FALSE)
```

```
Pval = numeric()
log2FoldChange = numeric()
MRE_mean = numeric()
Habitual_mean = numeric()
for(i in 1:ncol(MetPhlanT)){
  WilcoxTest = wilcox.test(MetPhlanTmeta[,i]~MetPhlanTmeta$Diet)
  Pval = c(Pval, WilcoxTest$p.value)
  Mmean = mean(MetPhlanTmeta[which(MetPhlanTmeta$Diet == "MRE"),i])
  Hmean = mean(MetPhlanTmeta[which(MetPhlanTmeta$Diet == "Habitual"),i])
  log2FoldChange = c(log2FoldChange, log2(Mmean/Hmean))
  MRE_mean = c(MRE_mean, Mmean)
  Habitual_mean = c(Habitual_mean, Hmean)
}

TestDF = data.frame(Pathway = rownames(MetPhlan), log2FoldChange, MRE_mean,
                    Habitual_mean, Pval, stringsAsFactors = FALSE)
TestDF05 = TestDF[which(TestDF$Pval < 0.05),]
TestDF05Ord = TestDF05[order(abs(TestDF05$log2FoldChange), decreasing = TRUE),]
write.csv(TestDF05Ord, "./ParsedData/Metaphlan2_Species_WilcoxTest_MREvsHab_AllSamps_05.csv",
          row.names = FALSE)

# stat tests for each tmpt
Tmpts = c(0, 5, 10, 24, 48)
for(h in 1:length(Tmpts)){
  PathSub = MetPhlanTmeta[which(MetPhlanTmeta$Time_Point.hrs. == Tmpts[h]),]
  Pval = numeric()
  log2FoldChange = numeric()
  MRE_mean = numeric()
```

```
  Habitual_mean = numeric()
  for(i in 1:ncol(MetPhlanT)){
    WilcoxTest = wilcox.test(PathSub[,i]~PathSub$Diet)
    Pval = c(Pval, WilcoxTest$p.value)
    Mmean = mean(PathSub[which(PathSub$Diet == "MRE"),i])
    Hmean = mean(PathSub[which(PathSub$Diet == "Habitual"),i])
    log2FoldChange = c(log2FoldChange, log2(Mmean/Hmean))
    MRE_mean = c(MRE_mean, Mmean)
    Habitual_mean = c(Habitual_mean, Hmean)
  }
  TestDF = data.frame(Pathway = rownames(MetPhlan), log2FoldChange, MRE_mean,
                   Habitual_mean, Pval, stringsAsFactors = FALSE)
  TestDF05 = TestDF[which(TestDF$Pval < 0.05),]
  TestDF05Ord = TestDF05[order(abs(TestDF05$log2FoldChange), decreasing = TRUE),]
  write.csv(TestDF05Ord, paste("./ParsedData/Metaphlan2_Species_WilcoxTest_MREvsHab_Tmpt_",
Tmpts[h],
                          "_Samps_05.csv", sep = ""), row.names = FALSE)
}

#### compare HAB 0 and 21 days, compare MRE 0 and 21 days, then compare/contrast results
TmptCompare = list(0, 5, 10, 24, 48, c(5, 10, 24))
for(h in 1:length(TmptCompare)){
  TmptTab = MetPhlanTmeta[which(MetPhlanTmeta$Time_Point.hrs. %in% TmptCompare[[h]]),]
  HABTab = TmptTab[which(TmptTab$Diet == "Habitual" & TmptTab$Date %in% c("Day0", "Day21")),]
  MRETab = TmptTab[which(TmptTab$Diet == "MRE" & TmptTab$Date %in% c("Day0", "Day21")),]
  # compare HAB btw days 0 and 21
  Pval = numeric()
  log2FoldChange = numeric()
  Day0_mean = numeric()
```

```
Day21_mean = numeric()
for(i in 1:ncol(MetPhlanT)){
  WilcoxTest = wilcox.test(HABTab[,i]~HABTab$Date)
  Pval = c(Pval, WilcoxTest$p.value)
  Mean0 = mean(HABTab[which(HABTab$Date == "Day0"),i])
  Mean21 = mean(HABTab[which(HABTab$Date == "Day21"),i])
  log2FoldChange = c(log2FoldChange, log2(Mean21/Mean0))
  Day0_mean = c(Day0_mean, Mean0)
  Day21_mean = c(Day21_mean, Mean21)
}
HabDF = data.frame(Species = colnames(MetPhlanT), log2FoldChange, Day0_mean,
                   Day21_mean, Pval, stringsAsFactors = FALSE)
HabDF05 = HabDF[which(HabDF$Pval < 0.05),]
HabDF05Ord = HabDF05[order(abs(HabDF05$log2FoldChange), decreasing = TRUE),]
if(nrow(HabDF05Ord) > 0){
  write.csv(HabDF05Ord,
paste("./ParsedData/Metaphlan2_Species_WilcoxTest_HAB_Day0vs21_Tmpt_",
                            paste(TmptCompare[[h]], collapse = "_"),
                            "_Samps_05.csv", sep = ""), row.names = FALSE)
}

# compare MRE btw days 0 and 21
Pval = numeric()
log2FoldChange = numeric()
Day0_mean = numeric()
Day21_mean = numeric()
for(i in 1:ncol(MetPhlanT)){
  WilcoxTest = wilcox.test(MRETab[,i]~MRETab$Date)
  Pval = c(Pval, WilcoxTest$p.value)
```

```
    Mean0 = mean(MRETab[which(MRETab$Date == "Day0"),i])
    Mean21 = mean(MRETab[which(MRETab$Date == "Day21"),i])
    log2FoldChange = c(log2FoldChange, log2(Mean21/Mean0))
    Day0_mean = c(Day0_mean, Mean0)
    Day21_mean = c(Day21_mean, Mean21)
  }
  MreDF = data.frame(Species = colnames(MetPhlanT), log2FoldChange, Day0_mean,
                     Day21_mean, Pval, stringsAsFactors = FALSE)
  MreDF05 = MreDF[which(MreDF$Pval < 0.05),]
  MreDF05Ord = MreDF05[order(abs(MreDF05$log2FoldChange), decreasing = TRUE),]
  if(nrow(MreDF05Ord) > 0){
    write.csv(MreDF05Ord,
paste("./ParsedData/Metaphlan2_Species_WilcoxTest_MRE_Day0vs21_Tmpt_",
                          paste(TmptCompare[[h]], collapse = "_"),
                          "_Samps_05.csv", sep = ""), row.names = FALSE)
  }
  print(paste(nrow(HabDF05Ord), nrow(MreDF05Ord)))
}


### same as above but for genus
MetPhlan = read.csv("./Metaphlan2/MetaPhlan2_AllSamps_mergedAbund_genus.csv",
                    header = TRUE, stringsAsFactors = FALSE)
rownames(MetPhlan) = MetPhlan$ID
MetPhlan = MetPhlan[,-1]
MetPhlanT = t(MetPhlan)
rownames(MetPhlanT) = gsub(".WGS", "", rownames(MetPhlanT))
MetPhlanTmeta = data.frame(MetPhlanT[which(rownames(MetPhlanT) %in% MREMap$X.SampleID),],
                           MREMap, stringsAsFactors = FALSE)
TmptCompare = list(0, 5, 10, 24, 48, c(5, 10, 24))
```

```
for(h in 1:length(TmptCompare)){
  TmptTab = MetPhlanTmeta[which(MetPhlanTmeta$Time_Point.hrs. %in% TmptCompare[[h]]),]
  HABTab = TmptTab[which(TmptTab$Diet == "Habitual" & TmptTab$Date %in% c("Day0", "Day21")),]
  MRETab = TmptTab[which(TmptTab$Diet == "MRE" & TmptTab$Date %in% c("Day0", "Day21")),]
  # compare HAB btw days 0 and 21
  Pval = numeric()
  log2FoldChange = numeric()
  Day0_mean = numeric()
  Day21_mean = numeric()
  for(i in 1:ncol(MetPhlanT)){
    WilcoxTest = wilcox.test(HABTab[,i]~HABTab$Date)
    Pval = c(Pval, WilcoxTest$p.value)
    Mean0 = mean(HABTab[which(HABTab$Date == "Day0"),i])
    Mean21 = mean(HABTab[which(HABTab$Date == "Day21"),i])
    log2FoldChange = c(log2FoldChange, log2(Mean21/Mean0))
    Day0_mean = c(Day0_mean, Mean0)
    Day21_mean = c(Day21_mean, Mean21)
  }
  HabDF = data.frame(Genus = colnames(MetPhlanT), log2FoldChange, Day0_mean,
                     Day21_mean, Pval, stringsAsFactors = FALSE)
  HabDF05 = HabDF[which(HabDF$Pval < 0.05),]
  HabDF05Ord = HabDF05[order(abs(HabDF05$log2FoldChange), decreasing = TRUE),]
  if(nrow(HabDF05Ord) > 0){
    write.csv(HabDF05Ord, 
paste("./ParsedData/Metaphlan2_Genus_WilcoxTest_HAB_Day0vs21_Tmpt_",
                          paste(TmptCompare[[h]], collapse = "_"),
                          "_Samps_05.csv", sep = ""), row.names = FALSE)
  }
```

```
  # compare MRE btw days 0 and 21
  Pval = numeric()
  log2FoldChange = numeric()
  Day0_mean = numeric()
  Day21_mean = numeric()
  for(i in 1:ncol(MetPhlanT)){
    WilcoxTest = wilcox.test(MRETab[,i]~MRETab$Date)
    Pval = c(Pval, WilcoxTest$p.value)
    Mean0 = mean(MRETab[which(MRETab$Date == "Day0"),i])
    Mean21 = mean(MRETab[which(MRETab$Date == "Day21"),i])
    log2FoldChange = c(log2FoldChange, log2(Mean21/Mean0))
    Day0_mean = c(Day0_mean, Mean0)
    Day21_mean = c(Day21_mean, Mean21)
  }
  MreDF = data.frame(Genus = colnames(MetPhlanT), log2FoldChange, Day0_mean,
                     Day21_mean, Pval, stringsAsFactors = FALSE)
  MreDF05 = MreDF[which(MreDF$Pval < 0.05),]
  MreDF05Ord = MreDF05[order(abs(MreDF05$log2FoldChange), decreasing = TRUE),]
  if(nrow(MreDF05Ord) > 0){
    write.csv(MreDF05Ord, 
paste("./ParsedData/Metaphlan2_Genus_WilcoxTest_MRE_Day0vs21_Tmpt_",
                           paste(TmptCompare[[h]], collapse = "_"),
                           "_Samps_05.csv", sep = ""), row.names = FALSE)
  }
  print(paste(nrow(HabDF05Ord), nrow(MreDF05Ord)))
}
```

Code/Plot_metawrap.R
0100777 0000000 0000000 00000015161 13506732014 011634 0

```
ustar 00
## analyze and plot bin abundances

## assembly and binning with metaWRAP


## load packages

library(heatmaply)

library(ggplot2)

library(htmlwidgets)

library(RColorBrewer)

library(vegan)

library(labdsv)

library(reshape2)


setwd("/Data1/NatickSeq/Natick_2018/Natick_WGS/")


# load metadata

MapFile =
read.table("/Data1/NatickSeq/Natick_2018/Natick_16S/QIIME2_analysis/Natick_MREferm_MappingFile
_2018.txt",

           sep = "\t", comment.char = "", header = TRUE, stringsAsFactors = FALSE)

MREMap = MapFile[which(MapFile$Study == "MREferm"),]


Diet = c("Habitual", "MRE")

Date = c("Day0", "Day21")

TimesToAnalyze = c(5, 10, 24, 48)

Tmpt = c(0, 5, 10, 24, 48)

SampsToKeep = MREMap$X.SampleID[which(MREMap$Date %in% Date & MREMap$Time_Point.hrs.
%in% Tmpt)]

MREMapNoPool = MREMap[which(MREMap$X.SampleID %in% SampsToKeep),]
```

```
# load bin abundance table

# from metaWRAP tutorial: Note that the abundances are expressed as "genome copies per million reads",

# and are calculated with Salmon in a simmilar way like TPM (transcripts per million) is calculated in

# RNAseq analysis. As such, they should be already standardized to the individual sample size.

BinAbund = read.table("./metaWRAP/QUANT_BINS/bin_abundance_table.tab", sep = "\t", header = TRUE,

                      stringsAsFactors = FALSE, row.names = 1)

colnames(BinAbund) = gsub("_clean", "", colnames(BinAbund))

BinAbund = t(BinAbund[,which(colnames(BinAbund) %in% MREMap$X.SampleID)])


# load bin taxonomy table

BinTax = read.table("./metaWRAP/BIN_CLASSIFICATION/bin_taxonomy.tab", sep = "\t", header = FALSE,

                    col.names = c("Bin", "Tax"), stringsAsFactors = FALSE)

BinTax$Bin = gsub(".\\w*.fa", "", BinTax$Bin)


# take most specific tax level for each bin

Tax = character()

for(i in 1:nrow(BinTax)){

  mysplit = strsplit(BinTax$Tax[i], ";")

  myTaxlev = length(mysplit[[1]])

  Tax = c(Tax, mysplit[[1]][myTaxlev])

}


BinTaxNames = paste(colnames(BinAbund), Tax[match(colnames(BinAbund), BinTax$Bin)], sep = "_")

BinAbundTax = BinAbund

colnames(BinAbundTax) = BinTaxNames

BinAbundTax = BinAbundTax[match(MREMap$X.SampleID, rownames(BinAbundTax)),]
```

```
# PCoA

Dist = vegdist(BinAbundTax, method = "bray")

mypco = pco(Dist, k = 10)

colnames(mypco$points) = paste(rep("PC", ncol(mypco$points)),

                          1:ncol(mypco$points), sep = "")

pc1 = round(mypco$eig[1]/sum(mypco$eig) * 100, digits = 1)

pc2 = round(mypco$eig[2]/sum(mypco$eig) * 100, digits = 1)

pc3 = round(mypco$eig[3]/sum(mypco$eig) * 100, digits = 1)

FullPCo = data.frame(MREMap, mypco$points, stringsAsFactors = FALSE)


pdf("./Plots/PCoA/metaWRAP_BinAbunds_bray_PCoA.pdf")

p = ggplot(data = FullPCo, aes(x = PC1, y = PC2, colour = paste(Diet, Date), shape =
factor(Time_Point.hrs.))) +

  geom_point(size = 3) + theme_bw() +

  labs(x = paste("PC1 (", pc1, "%)", sep = ""),

      y = paste("PC2 (", pc2, "%)", sep = ""),

      title = paste(" Bray-Curtis", sep = "")) +

  scale_colour_brewer(palette="Set1")

print(p)

dev.off()



heatmaply(t(log2(BinAbundTax + 1)), dendrogram = "both",

        col_side_colors = data.frame(Diet = MREMap$Diet, Date = MREMap$Date, Tmpt =
MREMap$Time_Point.hrs.),

        colors = colorRampPalette(rev(brewer.pal(n = 7, name = "RdYlBu")))(100)) %>%

  layout(margin = list(l = 100, b = 120)) %>% print %>%


saveWidget(file=paste("/Data1/NatickSeq/Natick_2018/Natick_WGS/Plots/Heatmaps/metaWRAP_binA
bund_heatmap_log2plus1.html",
```

```
                  sep = ""), selfcontained = FALSE)

mydf = BinAbundTax[which(rownames(BinAbundTax) %in% MREMapNoPool$X.SampleID),]
BrayAdon = adonis(mydf ~ Diet*Date*Time_Point.hrs., method = "bray",
                data = MREMapNoPool, strata = MREMapNoPool$Vessel, perm = 999)
write.csv(as.data.frame(BrayAdon$aov.tab),
paste("./ParsedData/metawrap_BinAbund_Bray_adonis.csv", sep = ""),
          row.names = TRUE)



## calculate baseline differences for each taxa
colnames(BinAbundTax) = gsub(" ", ".", colnames(BinAbundTax))
colnames(BinAbundTax) = gsub("/", "_", colnames(BinAbundTax))
for(h in 1:ncol(BinAbundTax)){
  myTaxa = as.data.frame(BinAbundTax[,h])
  Diets = character()
  Dates = character()
  Times = numeric()
  Vessel = character()
  TaxDifFromBase = numeric()
  for(i in 1:length(Diet)){
    for(j in 1:length(Date)){
      Vessels = unique(MREMapNoPool$Vessel[which(MREMapNoPool$Diet == Diet[i] &
MREMapNoPool$Date == Date[j])])
      for(k in 1:length(Vessels)){
        Samp0 = MREMapNoPool$X.SampleID[which(MREMapNoPool$Vessel == Vessels[k] &
MREMapNoPool$Time_Point.hrs. == 0)]
        for(l in 1:length(TimesToAnalyze)){
          mysamp = MREMapNoPool$X.SampleID[which(MREMapNoPool$Vessel == Vessels[k] &
                                        MREMapNoPool$Time_Point.hrs. == TimesToAnalyze[l])]
```

```
        print(paste(Diet[i], Date[j], Vessels[k], TimesToAnalyze[l], mysamp))
        Diets = c(Diets, Diet[i])
        Dates = c(Dates, Date[j])
        Vessel = c(Vessel, Vessels[k])
        Times = c(Times, TimesToAnalyze[l])
        TaxDifFromBase = c(TaxDifFromBase, myTaxa[mysamp,] - myTaxa[Samp0,])
      }
    }
  }
 }

 TotDF = data.frame(Diets, Dates, Vessel, Times, TaxDifFromBase, stringsAsFactors = FALSE)

 pdf(paste("./Plots/Baseline/metawrap_trends/DistToBase_overtime_metawrap_bin_",
       colnames(BinAbundTax)[h], ".pdf", sep = ""))
 p = ggplot(TotDF, aes(x = Times, y = TaxDifFromBase, group = paste(Diets, Dates, Vessel),
              color = paste(Diets, Dates))) +
   geom_line()
 print(p)
 dev.off()
}
```

Code/Plot_permanova.R 0100777 0000000 0000000
00000001464 13504746761 012021 0 ustar
00

```
## load packages

library(ggplot2)
library(RColorBrewer)
```

```
library(reshape2)


setwd("/Data1/NatickSeq/Natick_2018/Natick_WGS/")


# load permanova results
PermRes = read.csv("./ParsedData/WGS_and_16S_PERMANOVAs.csv", header = TRUE, stringsAsFactors
= FALSE)
PermRes = PermRes[which(PermRes$Term != "Total"),]
DataMetric = paste(PermRes$DataType, PermRes$DistMetric, sep = " ")
mydf = data.frame(DataMetric, PermRes, stringsAsFactors = FALSE)
mydf$DataMetric = factor(mydf$DataMetric, levels = unique(mydf$DataMetric))
mydf$Term = factor(mydf$Term, levels = unique(mydf$Term))


pdf("./Plots/PERMANOVA_R2_barplot.pdf")
p = ggplot(mydf, aes(x = DataMetric, y = R2, fill = Term)) +
  geom_bar(stat = "identity") +
  theme(axis.text.x = element_text(angle=45, hjust=1)) +
  scale_fill_brewer(palette = "Set1")
print(p)
dev.off()
```
Code/Plot_SpecificCAZyme.R 0100777 0000000 0000000
00000006554 13567274347 012641 0 ustar 00
```
library(ggplot2)
library(RColorBrewer)
library(reshape2)



setwd("/Data1/NatickSeq/Natick_2018/Natick_WGS/")


# load metadata
```

```
MapFile =
read.table("/Data1/NatickSeq/Natick_2018/Natick_16S/QIIME2_analysis/Natick_MREferm_MappingFile
_2018.txt",
                  sep = "\t", comment.char = "", header = TRUE, stringsAsFactors = FALSE)

MREMap = MapFile[which(MapFile$Study == "MREferm"),]


Diet = c("Habitual", "MRE")

Date = c("Day0", "Day21")

TimesToAnalyze = c(5, 10, 24)

Tmpt = c(0, 5, 10, 24)

SampsToKeep = MREMap$X.SampleID[which(MREMap$Date %in% Date & MREMap$Time_Point.hrs.
%in% Tmpt)]

MREMapNoPool = MREMap[which(MREMap$X.SampleID %in% SampsToKeep),]


# load cazymes mapped to contigs and bins table

CazToBin = read.csv("./ParsedData/metawrap_MapCAZymeContigsToBins.csv", header = TRUE,
stringsAsFactors = FALSE)


# load in contig abundances

CazToContig = read.csv("./ParsedData/metawrap_MapCAZymesToContigs.csv", header = TRUE,
stringsAsFactors = FALSE)


# load in contig taxonomy

ContigTax = read.table("./metaWRAP/AllContigsTaxClass/contig_taxonomy.tab", sep = "\t",
                   col.names = c("Contig", "Tax"), quote = "", stringsAsFactors = FALSE)

ContigTax$Tax[which(ContigTax$Tax == "")] = "unknown"

ContigTax$Tax[which(is.na(ContigTax$Tax))] = "unknown"

ContigTax$Tax = gsub(".*;", "", ContigTax$Tax)


# load in bin taxonomy
```

```
BinTax = read.table("./metaWRAP/AnvioBinTax/Bin_Taxonomy_megablast_gtdbk.txt", sep = "\t",
          header = TRUE, quote = "", stringsAsFactors = FALSE)


CazToContigBin = data.frame(CazToContig, Bin = CazToBin$Bin[match(CazToContig$Gene.ID,
CazToBin$Gene.ID)],
              Tax = ContigTax$Tax[match(CazToContig$Gene.ID, ContigTax$Contig)],
              stringsAsFactors = FALSE)
# change NAs to NotBinned
CazToContigBin$Bin[which(is.na(CazToContigBin$Bin))] = "NotBinned"
# change NAs to unknown
CazToContigBin$Tax[which(is.na(CazToContigBin$Tax))] = "unknown"
# clean up HMM names
CazToContigBin$HMM.Profile = gsub(".hmm", "", CazToContigBin$HMM.Profile)
# subset for samples
CazToContigBinSub = CazToContigBin[which(CazToContigBin$Sample %in%
MREMapNoPool$X.SampleID),]
# change to relative abundance
CazToContigBinSub$Count = CazToContigBinSub$Count/10^6



## subset for specific CAZyme
CAZofInt = c("GH13_14", "GT76", "CBM83", "CBM27")
for(i in 1:length(CAZofInt)){
  CazSub = CazToContigBinSub[which(CazToContigBinSub$HMM.Profile == CAZofInt[i]),]
  CazSubMeta = data.frame(CazSub, MREMapNoPool[match(CazSub$Sample,
MREMapNoPool$X.SampleID),],
             stringsAsFactors = FALSE)
  pdf(paste("./Plots/TaxBreakdowns/metawrap_Bins_Cazyme_", CAZofInt[i], "_ByDietDateTime.pdf", sep
= ""))
  p = ggplot(CazSubMeta, aes(x = factor(Time_Point.hrs.), y = Count, fill = Bin)) +
```

```
    geom_bar(stat = "summary", fun.y = "mean") +
    facet_grid(Diet~Date) +
    scale_fill_brewer(palette="Set3", name = "Bin") +
    labs(x = "Time (h)", y = "Relative abundance", title = CAZofInt[i])
  print(p)
  dev.off()

  pdf(paste("./Plots/TaxBreakdowns/metawrap_Tax_Cazyme_", CAZofInt[i], "_ByDietDateTime.pdf", sep
= ""))
  p = ggplot(CazSubMeta, aes(x = factor(Time_Point.hrs.), y = Count, fill = Tax)) +
    geom_bar(stat = "summary", fun.y = "mean") +
    facet_grid(Diet~Date) +
    scale_fill_brewer(palette="Set3", name = "Tax") +
    labs(x = "Time (h)", y = "Relative abundance", title = CAZofInt[i])
  print(p)
  dev.off()
}
```

Code/Plot_SpecificHUMAnN2Path.R 0100777 0000000 0000000
00000004776 13563321543 013425 0 ustar
00

```
## load packages
library(heatmaply)
library(ggplot2)
library(htmlwidgets)
library(RColorBrewer)
library(vegan)
```

```
library(labdsv)

library(reshape2)


setwd("/Data1/NatickSeq/Natick_2018/Natick_WGS/")


# load metadata

MapFile =
read.table("/Data1/NatickSeq/Natick_2018/Natick_16S/QIIME2_analysis/Natick_MREferm_MappingFile
_2018.txt",

                sep = "\t", comment.char = "", header = TRUE, stringsAsFactors = FALSE)

MREMap = MapFile[which(MapFile$Study == "MREferm"),]


Diet = c("MRE", "Habitual")

Date = c("Day0", "Day21")

Tmpt = c(0, 5, 10, 24)

SampsToKeep = MREMap$X.SampleID[which(MREMap$Date %in% Date & MREMap$Time_Point.hrs.
%in% Tmpt)]

MREMapNoPool = MREMap[which(MREMap$X.SampleID %in% SampsToKeep),]


# Pathway analysis

PathAbundAll = read.table("./Humann2/Output/MRE_humann2_pathabundances_relabund.tsv",

                        sep = "\t", comment.char = "", header = TRUE, stringsAsFactors = FALSE, quote = "")

colnames(PathAbundAll) = c("Pathway", MREMap$X.SampleID)

rownames(PathAbundAll) = PathAbundAll$Pathway

PathAbundAll = PathAbundAll[,-1]

PathAbundAll = PathAbundAll[,which(colnames(PathAbundAll) %in% MREMapNoPool$X.SampleID)]


# plot pathways of interest

#"PWY-6737", "PWY-5101", "AEROBACTINSYN-PWY", "P164-PWY", "PWY0-1338"

PathwayOfInt = c("PWY-6737", "PWY-5101", "AEROBACTINSYN-PWY", "P164-PWY", "PWY0-1338")
```

```
for(i in 1:length(PathwayOfInt)){
  PathSub = PathAbundAll[grep(PathwayOfInt[i], rownames(PathAbundAll)),]
  # take top 12 species if that many
  if(nrow(PathSub) > 12){
    PathSub2 = PathSub[order(rowSums(PathSub), decreasing = TRUE)[1:12],]
  }else{
    PathSub2 = PathSub[order(rowSums(PathSub), decreasing = TRUE),]
  }
  rownames(PathSub2) = gsub(paste(PathwayOfInt[i], ":", sep = ""), "", rownames(PathSub2))
  rownames(PathSub2) = gsub(".*\\|", "", rownames(PathSub2))
  rownames(PathSub2) = gsub("g__.*s__", "", rownames(PathSub2))

  PathSub3 = t(PathSub2)
  PathMelt = melt(PathSub3)

  PathMelt = data.frame(PathMelt, MREMap[match(PathMelt$Var1, MREMap$X.SampleID),],
stringsAsFactors = FALSE)
  PathMelt$Var2 = factor(PathMelt$Var2, levels = rownames(PathSub2))

  pdf(paste("./Plots/TaxBreakdowns/HUMAnN2_PathBarBySp_", PathwayOfInt[i], ".pdf", sep = ""))
  p = ggplot(PathMelt, aes(x = factor(Time_Point.hrs.), y = value, fill = Var2)) +
    geom_bar(stat = "summary", fun.y = "mean", position = "dodge") +
    scale_fill_brewer(palette="Set3", name = "Species") + facet_grid(Diet~Date) +
    labs(x = "Tmpt", y = "Relative abundance", title = PathwayOfInt[i])
  print(p)
  dev.off()
}
```

Code/RandomForestWGS.R 0100777 0000000 0000000 00000023473 13521111476 012007 0 ustar 00
Random Forest analysis

```
library(randomForest)

library(plyr)

library(rfUtilities)

library(caret)

library(ggplot2)


setwd("/Data1/NatickSeq/Natick_2018/Natick_WGS/")


# load metadata

MapFile =
read.table("/Data1/NatickSeq/Natick_2018/Natick_16S/QIIME2_analysis/Natick_MREferm_MappingFile
_2018.txt",

                sep = "\t", comment.char = "", header = TRUE, stringsAsFactors = FALSE)

MREMap = MapFile[which(MapFile$Study == "MREferm"),]


# remove pool and 48h samples

Diet = c("Habitual", "MRE")

Date = c("Day0", "Day21")

Tmpt = c(0, 5, 10, 24)

SampsToKeep = MREMap$X.SampleID[which(MREMap$Date %in% Date & MREMap$Time_Point.hrs.
%in% Tmpt)]

MREMapNoPool = MREMap[which(MREMap$X.SampleID %in% SampsToKeep),]


# define times to analyze for change scores

TimesToAnalyze = c(5, 10, 24)
```

```
# define function to remove rare features
# adapted from https://github.com/LangilleLab/microbiome_helper/wiki/Random-Forest-Tutorial#removing-rare-features
# removes feature if above a threshold in less than a threshold of samples
remove_rare = function(table, nsamp, ncount){
  col2keep = c()
  cutoff = ceiling(nsamp * nrow(table))
  for(i in 1:ncol(table)){
    col_nonzero = length(which(table[,i] > ncount))
    if(col_nonzero > cutoff){
      col2keep <- c(col2keep, i)
    }
  }
  return(table[, col2keep, drop = FALSE])
}

# define function to calculate change scores from t=0
Baseline_a_df = function(Meta = Metatab, Abundances = mydf, TimesToAnalyze = TimesToAnalyze,
                         Diet = Diet, Date = Date){
  Sample = character()
  Diets = character()
  Dates = character()
  Times = numeric()
  Vessel = character()
  DifFromBase = data.frame()
  for(i in 1:length(Diet)){
    for(j in 1:length(Date)){
      Vessels = unique(Meta$Vessel[which(Meta$Diet == Diet[i] & Meta$Date == Date[j])])
      for(k in 1:length(Vessels)){
```

```
      Samp0 = Meta$X.SampleID[which(Meta$Vessel == Vessels[k] & Meta$Time_Point.hrs. == 0)]
      for(l in 1:length(TimesToAnalyze)){
        mysamp = Meta$X.SampleID[which(Meta$Vessel == Vessels[k] &
                                  Meta$Time_Point.hrs. == TimesToAnalyze[l])]
        print(paste(Diet[i], Date[j], Vessels[k], TimesToAnalyze[l], mysamp))
        Sample = c(Sample, mysamp)
        Diets = c(Diets, Diet[i])
        Dates = c(Dates, Date[j])
        Vessel = c(Vessel, Vessels[k])
        Times = c(Times, TimesToAnalyze[l])
        DifFromBase = rbind(DifFromBase, Abundances[mysamp,] - Abundances[Samp0,])
      }
    }
  }
}
colnames(DifFromBase) = colnames(Abundances)
DifFromBase = data.frame(Diet = Diets, Date = Dates, Vessel = Vessel, Time = Times,
                         DietDate = paste(Diets, Dates, sep = "_"), DifFromBase,
                         stringsAsFactors = FALSE)
rownames(DifFromBase) = Sample
return(DifFromBase)
}


# load MetaPhlan2 abundances
MetPhlan = read.csv("./Metaphlan2/MetaPhlan2_AllSamps_mergedAbund_species.csv",
                    header = TRUE, stringsAsFactors = FALSE)
rownames(MetPhlan) = MetPhlan$ID
MetPhlan = MetPhlan[,-1]
```

```
MetPhlanT = t(MetPhlan)

rownames(MetPhlanT) = gsub(".WGS", "", rownames(MetPhlanT))

MetPhlanTsub = MetPhlanT[which(rownames(MetPhlanT) %in% MREMapNoPool$X.SampleID),]

# remove rare species

Metsub = MetPhlanTsub

Metsub = remove_rare(table = Metsub, nsamp = 0.5, ncount = 0)


# calculate change scores from t=0

MetPhlanChange = Baseline_a_df(Meta = MREMapNoPool, Abundances = Metsub,

                        TimesToAnalyze = TimesToAnalyze, Diet = Diet, Date = Date)




##########################################################################################
###

# HUMAnN2 pathways

# Pathway analysis

PathAbundUnstrat =
read.table("./Humann2/Output/SplitPaths/MRE_humann2_pathabundances_cpm_unstratified.tsv",

                        sep = "\t", comment.char = "", header = TRUE, stringsAsFactors = FALSE, quote = "")

colnames(PathAbundUnstrat) = c("Pathway", MREMap$X.SampleID)

rownames(PathAbundUnstrat) = gsub(": ", "_", PathAbundUnstrat$Pathway)

PathAbundUnstrat = PathAbundUnstrat[,-1]

PathAbundUnstratNoUns = PathAbundUnstrat[which(!(rownames(PathAbundUnstrat) %in%
c("UNMAPPED", "UNINTEGRATED"))),]

PathAbundUnstratNoUns = PathAbundUnstratNoUns[,which(colnames(PathAbundUnstratNoUns) %in%
MREMapNoPool$X.SampleID)]


# remove rare species

Pathsub = t(PathAbundUnstratNoUns)
```

```
Pathsub = remove_rare(table = Pathsub, nsamp = 0.5, ncount = 0)


# calculate change scores from t=0

PathChange = Baseline_a_df(Meta = MREMapNoPool, Abundances = Pathsub,

                     TimesToAnalyze = TimesToAnalyze, Diet = Diet, Date = Date)



####################################################################################
###

# HUMAnN2 pathways

# Gene fam analysis

GeneFamUnstrat =
read.table("./Humann2/Output/SplitGenes/MRE_humann2_genefamilies_cpm_unstratified.tsv",

                   sep = "\t", comment.char = "", header = TRUE, stringsAsFactors = FALSE, quote = "")

colnames(GeneFamUnstrat) = c("GeneFamily", MREMap$X.SampleID)

rownames(GeneFamUnstrat) = GeneFamUnstrat$GeneFamily

GeneFamUnstrat = GeneFamUnstrat[,-1]

GeneFamUnstratNoUns = GeneFamUnstrat[which(!(rownames(GeneFamUnstrat) %in% c("UNMAPPED",
"UniRef90_unknown"))),]

GeneFamUnstratNoUns = GeneFamUnstratNoUns[,which(colnames(GeneFamUnstratNoUns) %in%
MREMapNoPool$X.SampleID)]


# remove rare species

Genesub = t(GeneFamUnstratNoUns)

Genesub = remove_rare(table = Genesub, nsamp = 0.5, ncount = 0)


# calculate change scores from t=0

GeneChange = Baseline_a_df(Meta = MREMapNoPool, Abundances = Genesub,

                   TimesToAnalyze = TimesToAnalyze, Diet = Diet, Date = Date)
```

```
##########################################################################################
###

# metaWRAP bin abundances

BinAbund = read.table("./metaWRAP/QUANT_BINS/bin_abundance_table.tab", sep = "\t", header =
TRUE,

                      stringsAsFactors = FALSE, row.names = 1)

colnames(BinAbund) = gsub("_clean", "", colnames(BinAbund))

BinAbund = t(BinAbund[,which(colnames(BinAbund) %in% MREMap$X.SampleID)])


# load bin taxonomy table

BinTax = read.table("./metaWRAP/BIN_CLASSIFICATION/bin_taxonomy.tab", sep = "\t", header = FALSE,

                    col.names = c("Bin", "Tax"), stringsAsFactors = FALSE)

BinTax$Bin = gsub(".\\w*.fa", "", BinTax$Bin)


# take most specific tax level for each bin

Tax = character()

for(i in 1:nrow(BinTax)){

  mysplit = strsplit(BinTax$Tax[i], ";")

  myTaxlev = length(mysplit[[1]])

  Tax = c(Tax, mysplit[[1]][myTaxlev])

}


BinTaxNames = paste(colnames(BinAbund), Tax[match(colnames(BinAbund), BinTax$Bin)], sep = "_")

BinAbundTax = BinAbund

colnames(BinAbundTax) = BinTaxNames

BinAbundTax = BinAbundTax[match(MREMapNoPool$X.SampleID, rownames(BinAbundTax)),]


# remove rare species
```

```
Binsub = BinAbundTax

Binsub = remove_rare(table = Binsub, nsamp = 0.5, ncount = 0)


# calculate change scores from t=0

BinChange = Baseline_a_df(Meta = MREMapNoPool, Abundances = Binsub,

                     TimesToAnalyze = TimesToAnalyze, Diet = Diet, Date = Date)




#########################################################################################
###

# cazyme abundances

CazAbun = read.csv("./ParsedData/metawrap_CAZymeAbund_BasedOnContigs.csv", header = TRUE,

                row.names = 1)

CazAbun = CazAbun[which(rownames(CazAbun) %in% MREMapNoPool$X.SampleID),]


# remove rare species

Cazsub = CazAbun

Cazsub = remove_rare(table = Cazsub, nsamp = 0.5, ncount = 0)


# calculate change scores from t=0

CazChange = Baseline_a_df(Meta = MREMapNoPool, Abundances = Cazsub,

                     TimesToAnalyze = TimesToAnalyze, Diet = Diet, Date = Date)
```
