# Supplementary material for: In vitro gut microbiome response to carbohydrate supplementation is acutely affected by a sudden change in diet
Source: BMC Microbiol. 2023 Jan 28;23:32. doi: 10.1186/s12866-023-02776-2 (PMC9883884; doi:10.1186/s12866-023-02776-2)
Supplement: Supplementary file 2 — Additional file 2. [file 12866_2023_2776_MOESM2_ESM.pptx]

## Slide 1
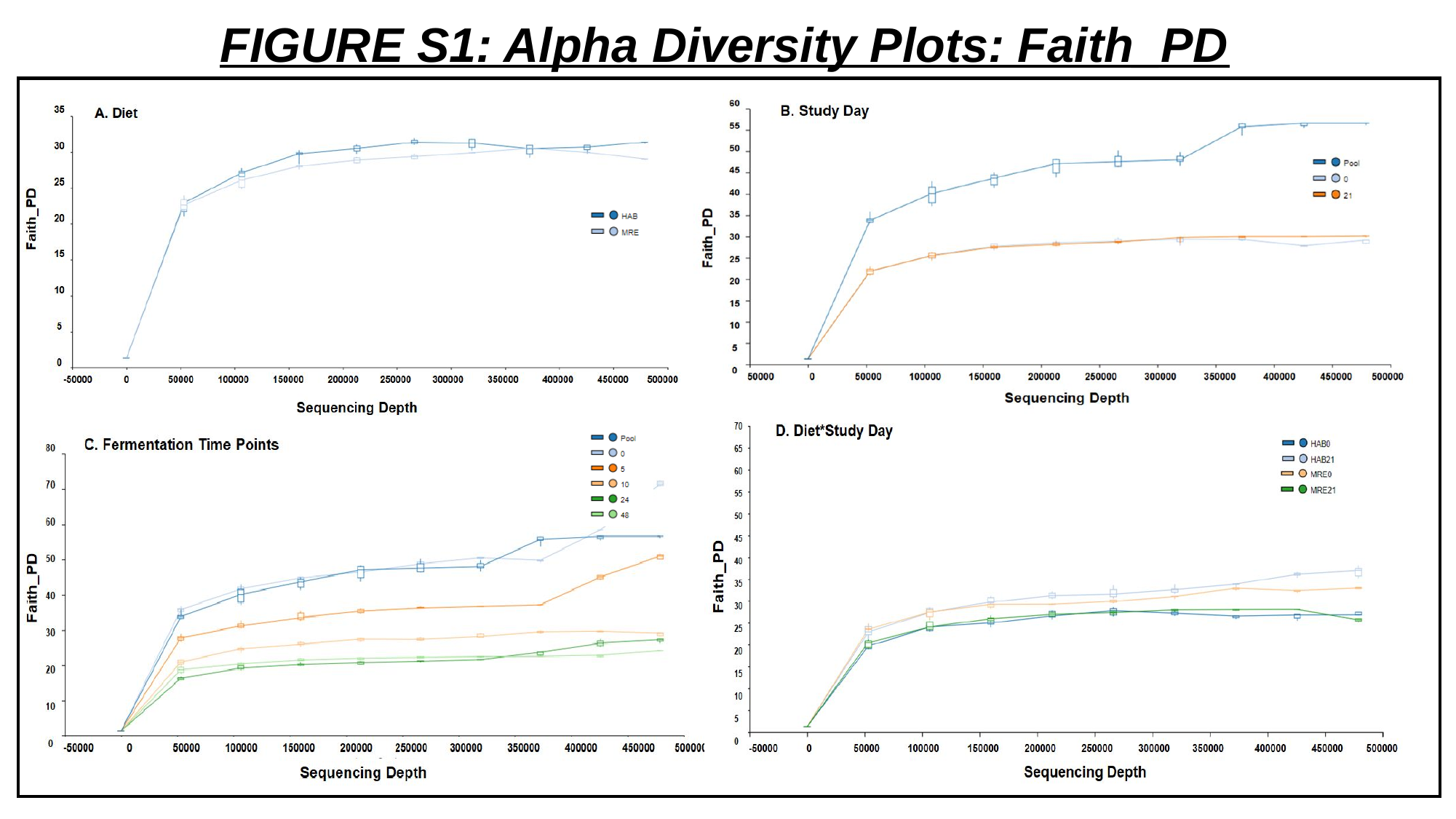

FIGURE S1: Alpha Diversity Plots: Faith_PD

## Slide 2
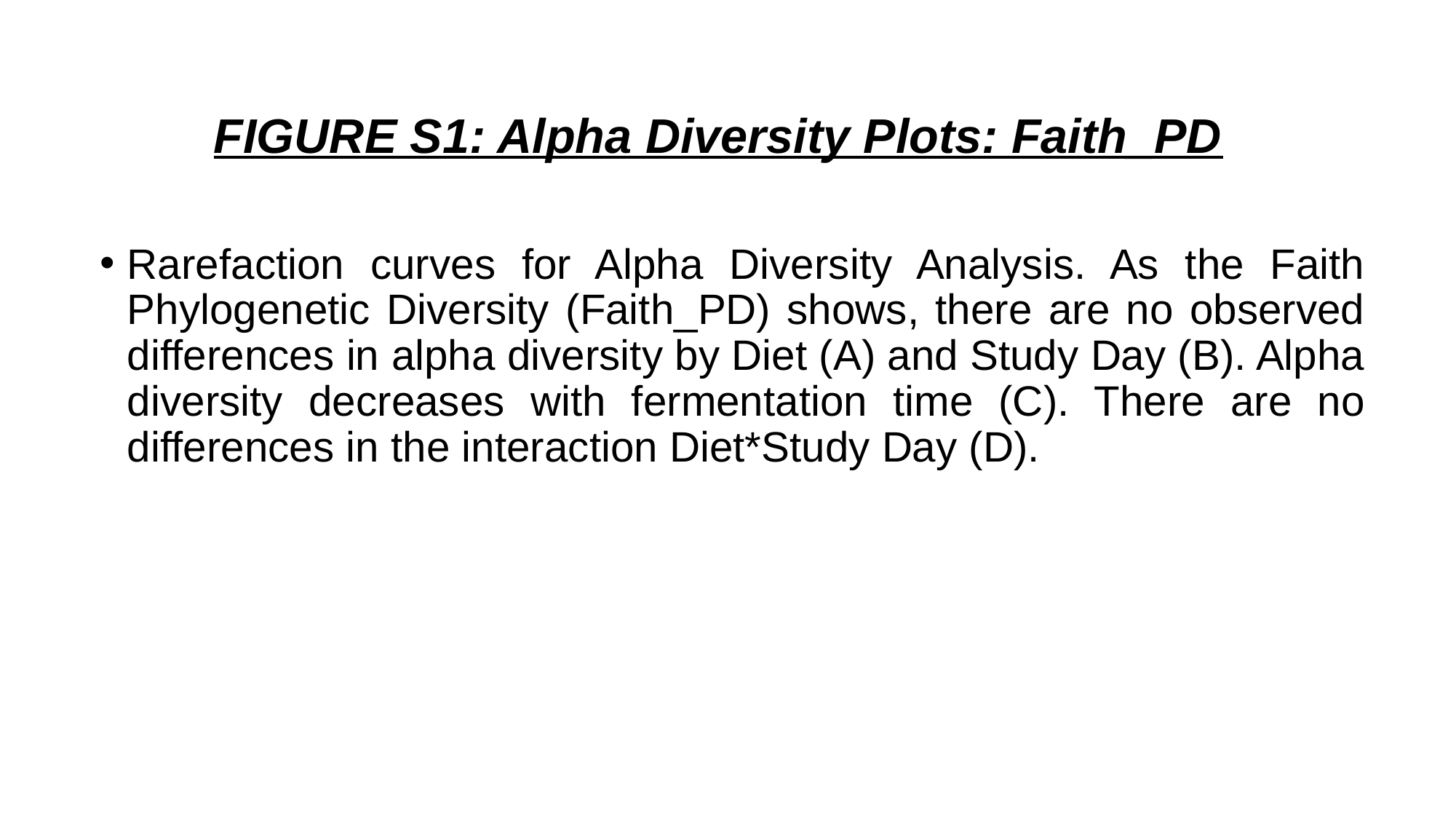

FIGURE S1: Alpha Diversity Plots: Faith_PD
Rarefaction curves for Alpha Diversity Analysis. As the Faith Phylogenetic Diversity (Faith_PD) shows, there are no observed differences in alpha diversity by Diet (A) and Study Day (B). Alpha diversity decreases with fermentation time (C). There are no differences in the interaction Diet*Study Day (D).

## Slide 3
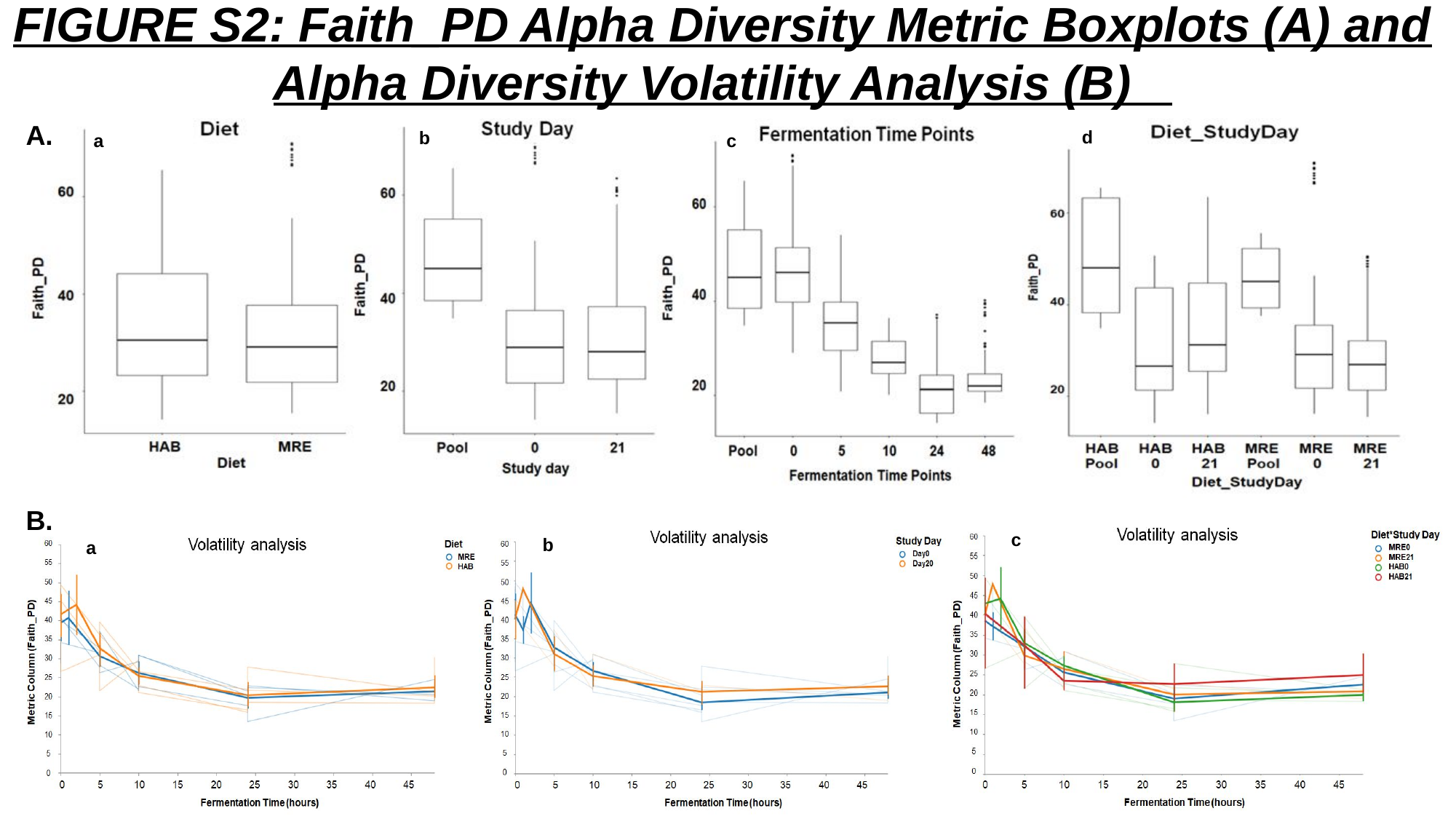

FIGURE S2: Faith_PD Alpha Diversity Metric Boxplots (A) and Alpha Diversity Volatility Analysis (B)
A.
d
b
c
a
B.
c
b
a

## Slide 4
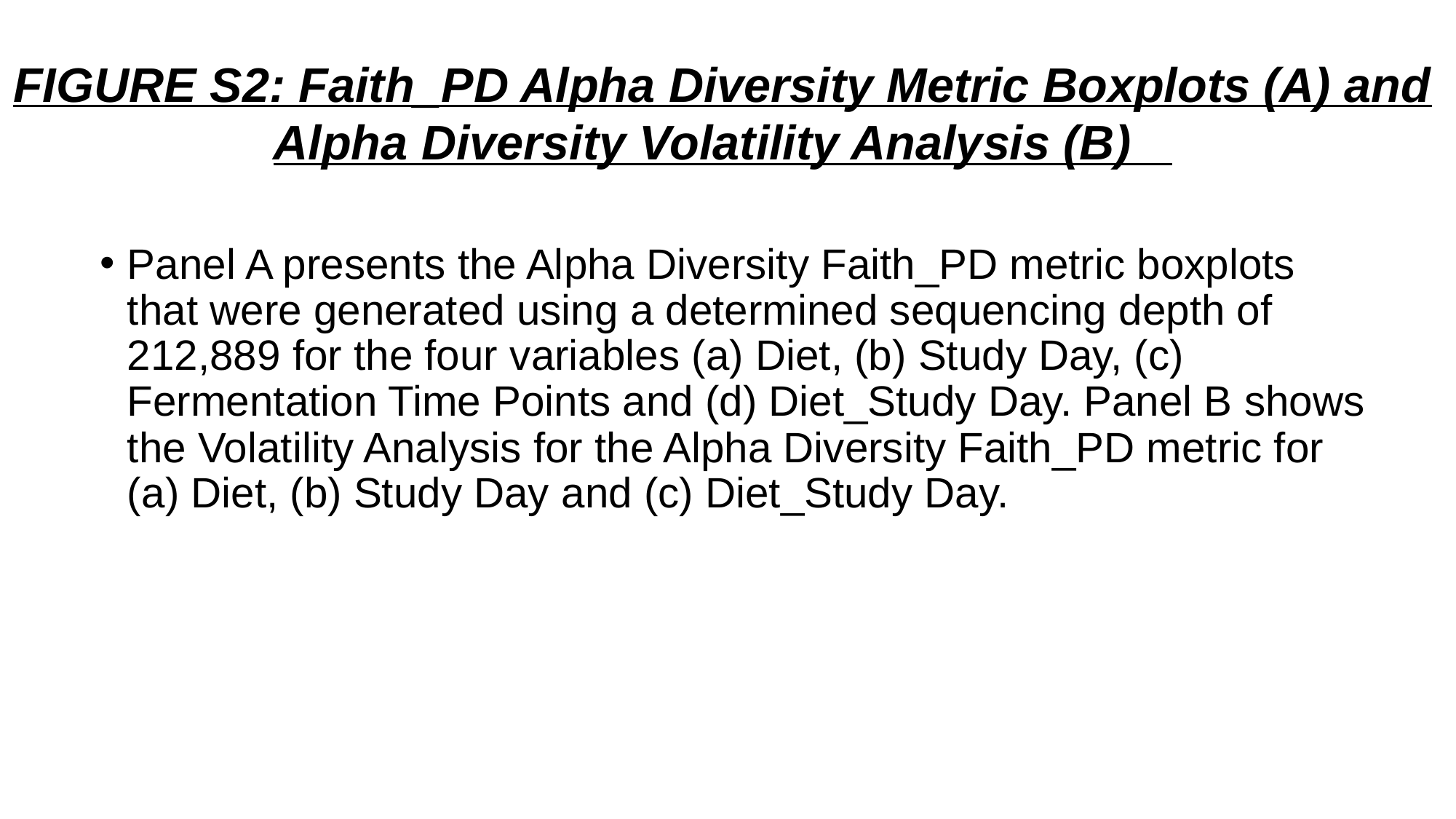

FIGURE S2: Faith_PD Alpha Diversity Metric Boxplots (A) and Alpha Diversity Volatility Analysis (B)
Panel A presents the Alpha Diversity Faith_PD metric boxplots that were generated using a determined sequencing depth of 212,889 for the four variables (a) Diet, (b) Study Day, (c) Fermentation Time Points and (d) Diet_Study Day. Panel B shows the Volatility Analysis for the Alpha Diversity Faith_PD metric for (a) Diet, (b) Study Day and (c) Diet_Study Day.

## Slide 5
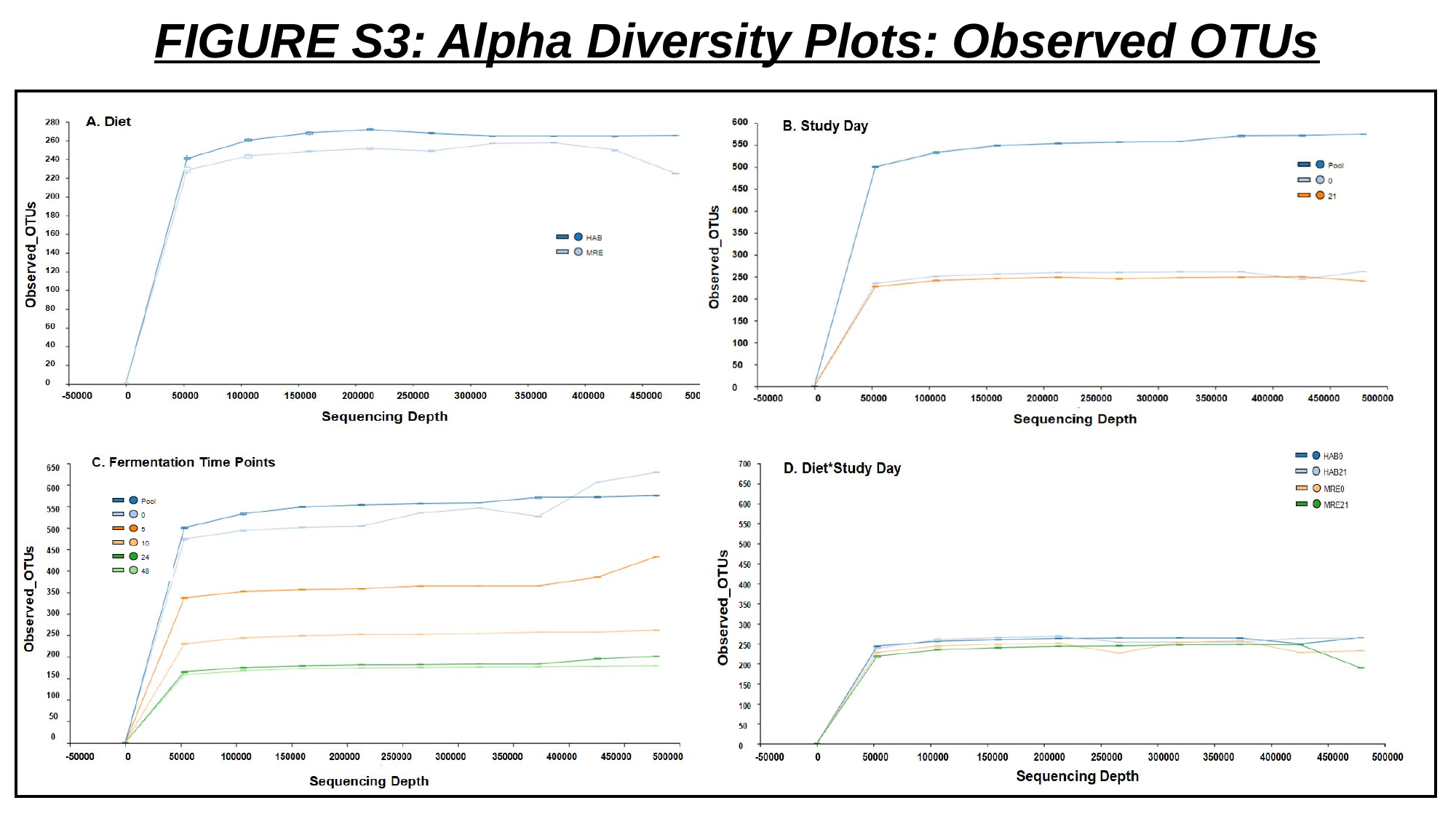

FIGURE S3: Alpha Diversity Plots: Observed OTUs

## Slide 6
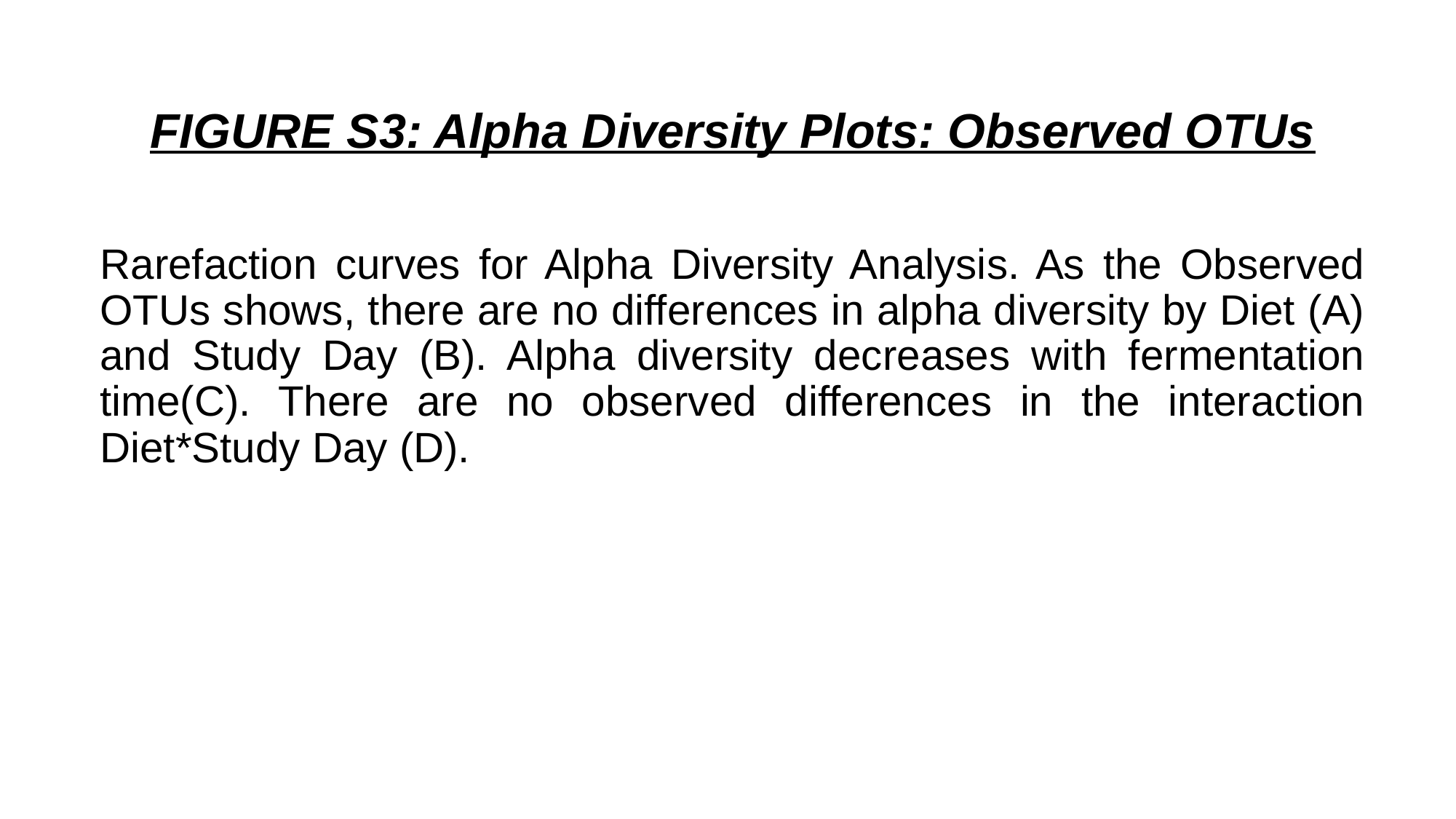

FIGURE S3: Alpha Diversity Plots: Observed OTUs
Rarefaction curves for Alpha Diversity Analysis. As the Observed OTUs shows, there are no differences in alpha diversity by Diet (A) and Study Day (B). Alpha diversity decreases with fermentation time(C). There are no observed differences in the interaction Diet*Study Day (D).

## Slide 7
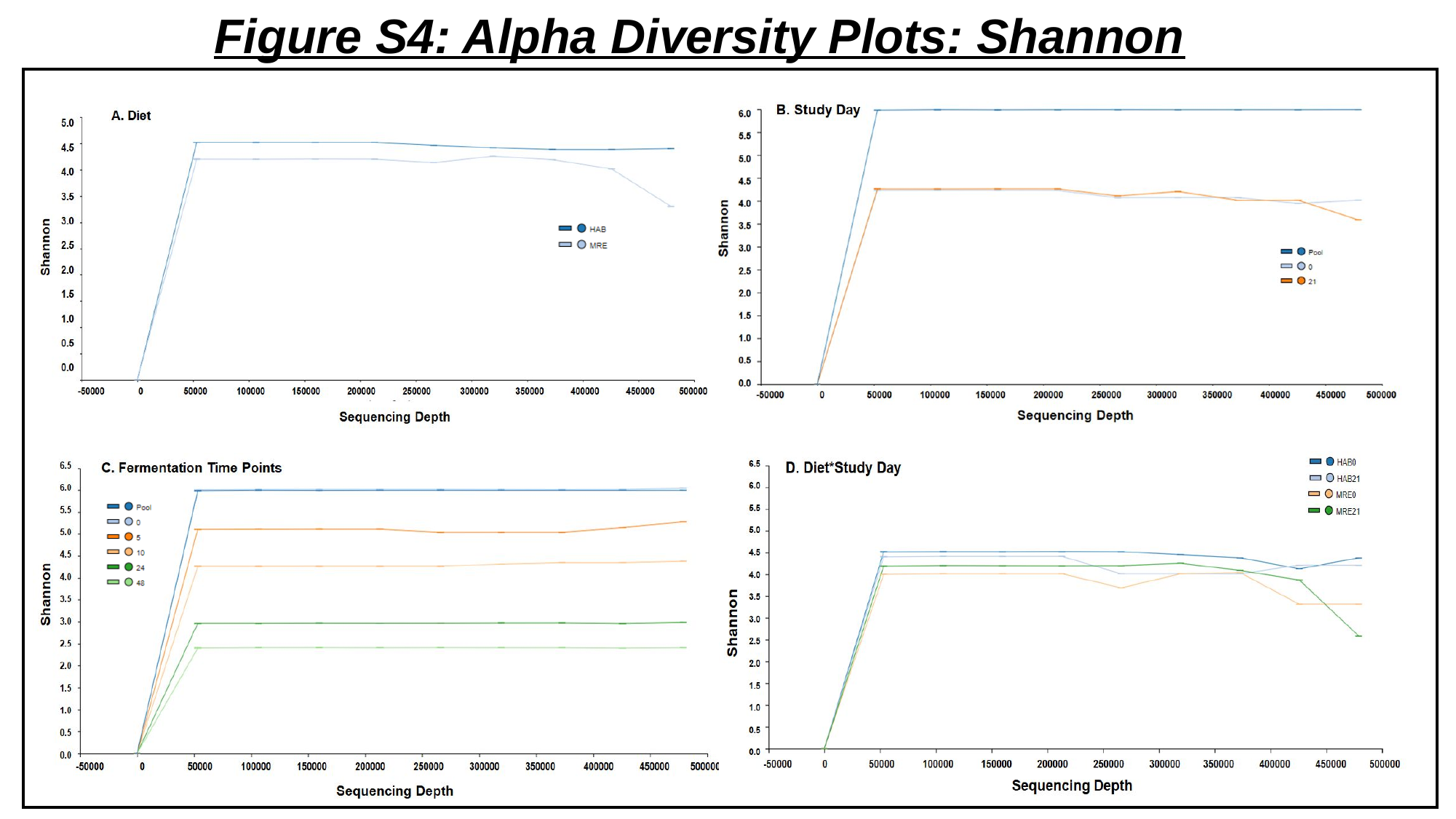

Figure S4: Alpha Diversity Plots: Shannon

## Slide 8
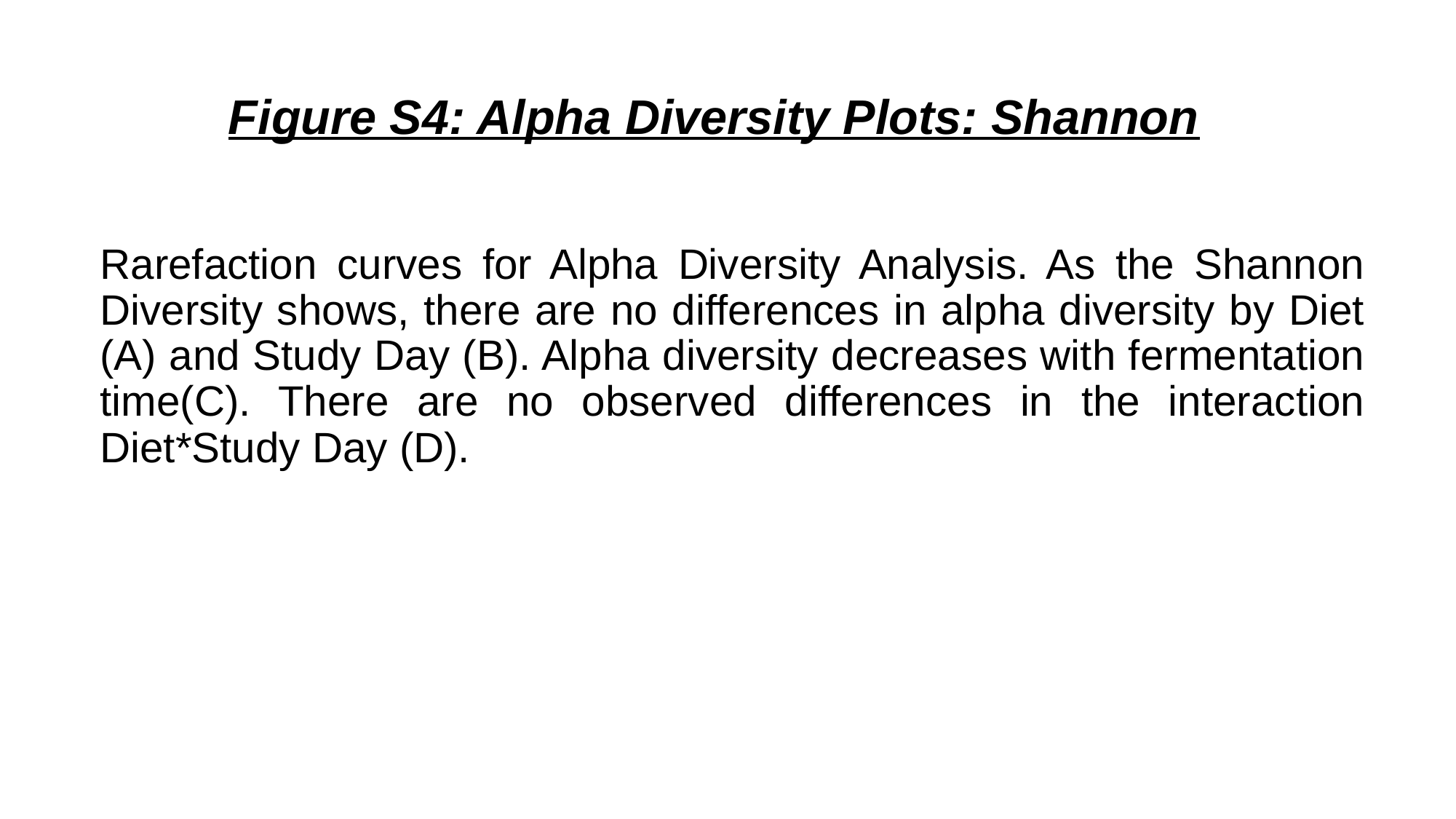

Figure S4: Alpha Diversity Plots: Shannon
Rarefaction curves for Alpha Diversity Analysis. As the Shannon Diversity shows, there are no differences in alpha diversity by Diet (A) and Study Day (B). Alpha diversity decreases with fermentation time(C). There are no observed differences in the interaction Diet*Study Day (D).

## Slide 9
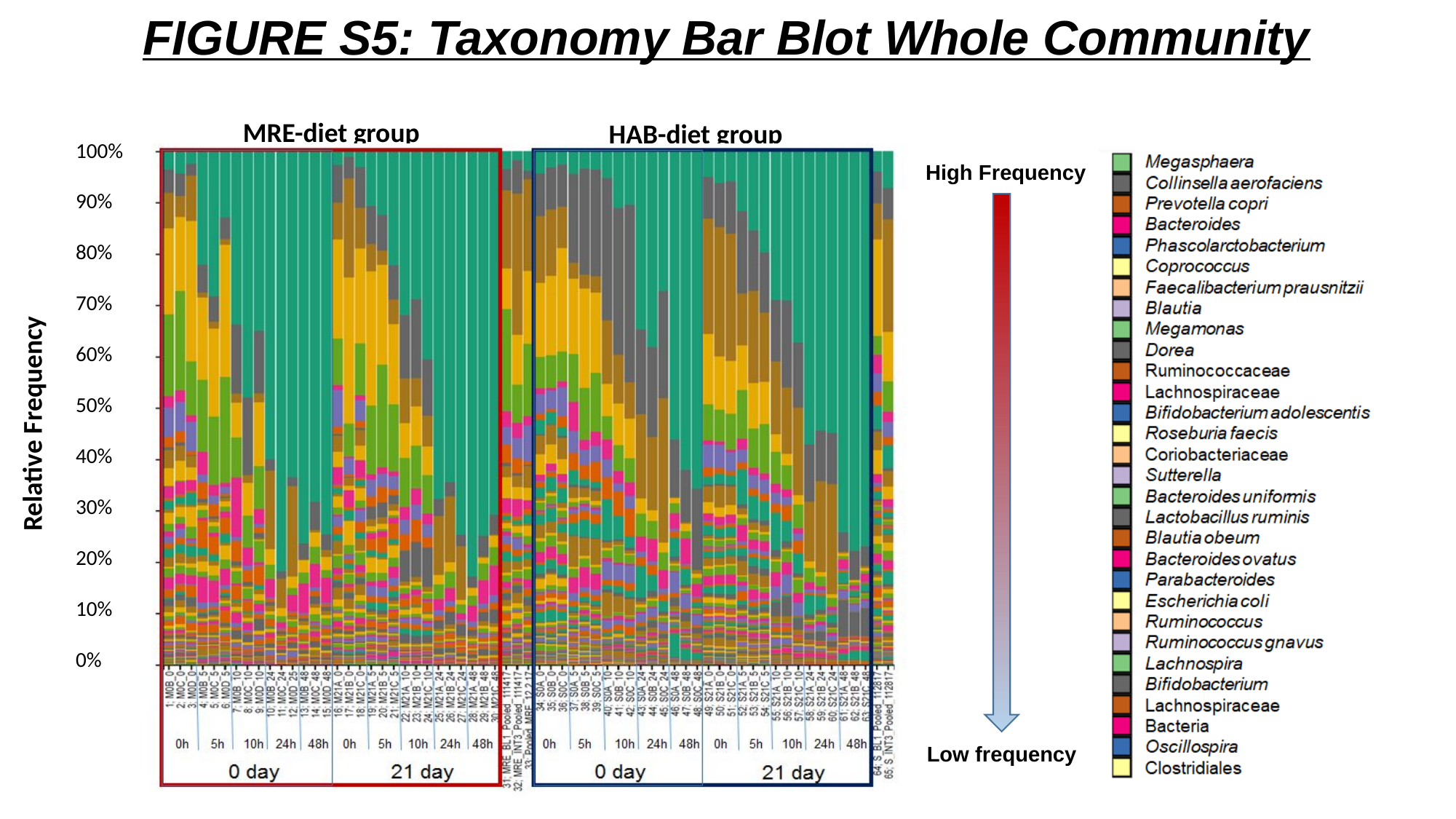

FIGURE S5: Taxonomy Bar Blot Whole Community
MRE-diet group
HAB-diet group
100%
90%
80%
70%
60%
50%
40%
30%
20%
10%
0%
Relative Frequency
High Frequency
Low frequency

## Slide 10
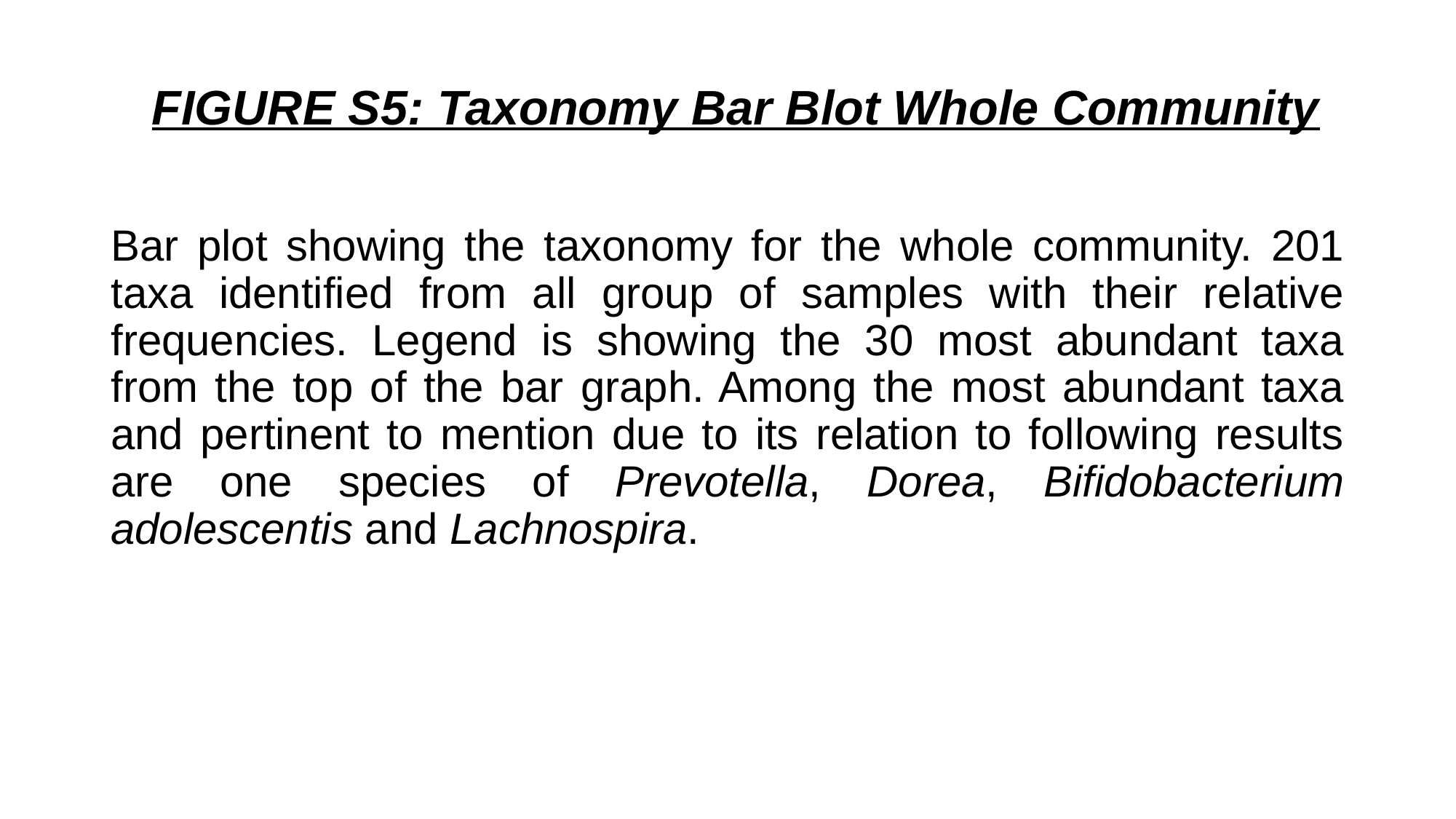

FIGURE S5: Taxonomy Bar Blot Whole Community
Bar plot showing the taxonomy for the whole community. 201 taxa identified from all group of samples with their relative frequencies. Legend is showing the 30 most abundant taxa from the top of the bar graph. Among the most abundant taxa and pertinent to mention due to its relation to following results are one species of Prevotella, Dorea, Bifidobacterium adolescentis and Lachnospira.

## Slide 11
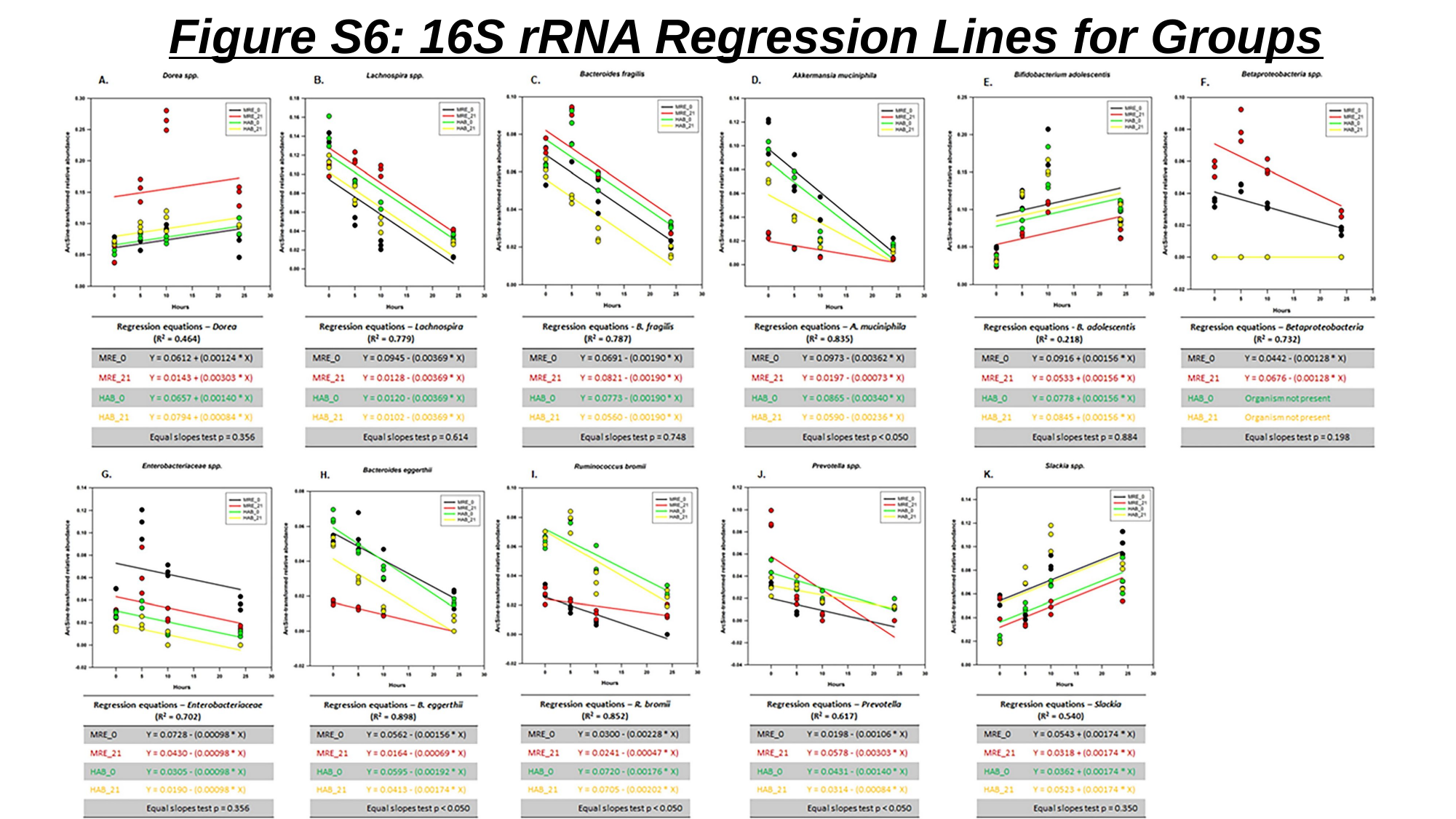

Figure S6: 16S rRNA Regression Lines for Groups

## Slide 12
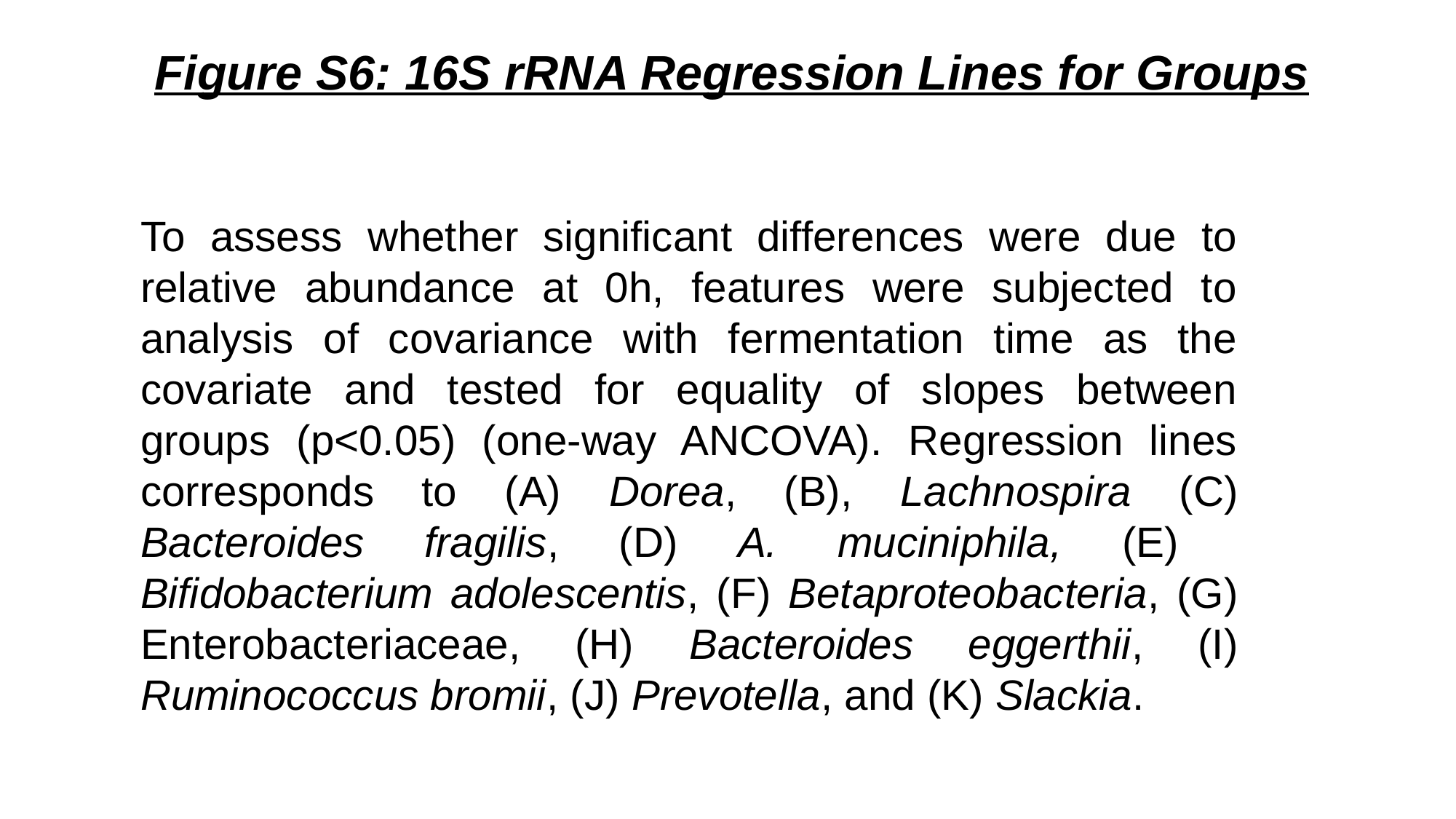

Figure S6: 16S rRNA Regression Lines for Groups
To assess whether significant differences were due to relative abundance at 0h, features were subjected to analysis of covariance with fermentation time as the covariate and tested for equality of slopes between groups (p<0.05) (one-way ANCOVA). Regression lines corresponds to (A) Dorea, (B), Lachnospira (C) Bacteroides fragilis, (D) A. muciniphila, (E) Bifidobacterium adolescentis, (F) Betaproteobacteria, (G) Enterobacteriaceae, (H) Bacteroides eggerthii, (I) Ruminococcus bromii, (J) Prevotella, and (K) Slackia.

## Slide 13
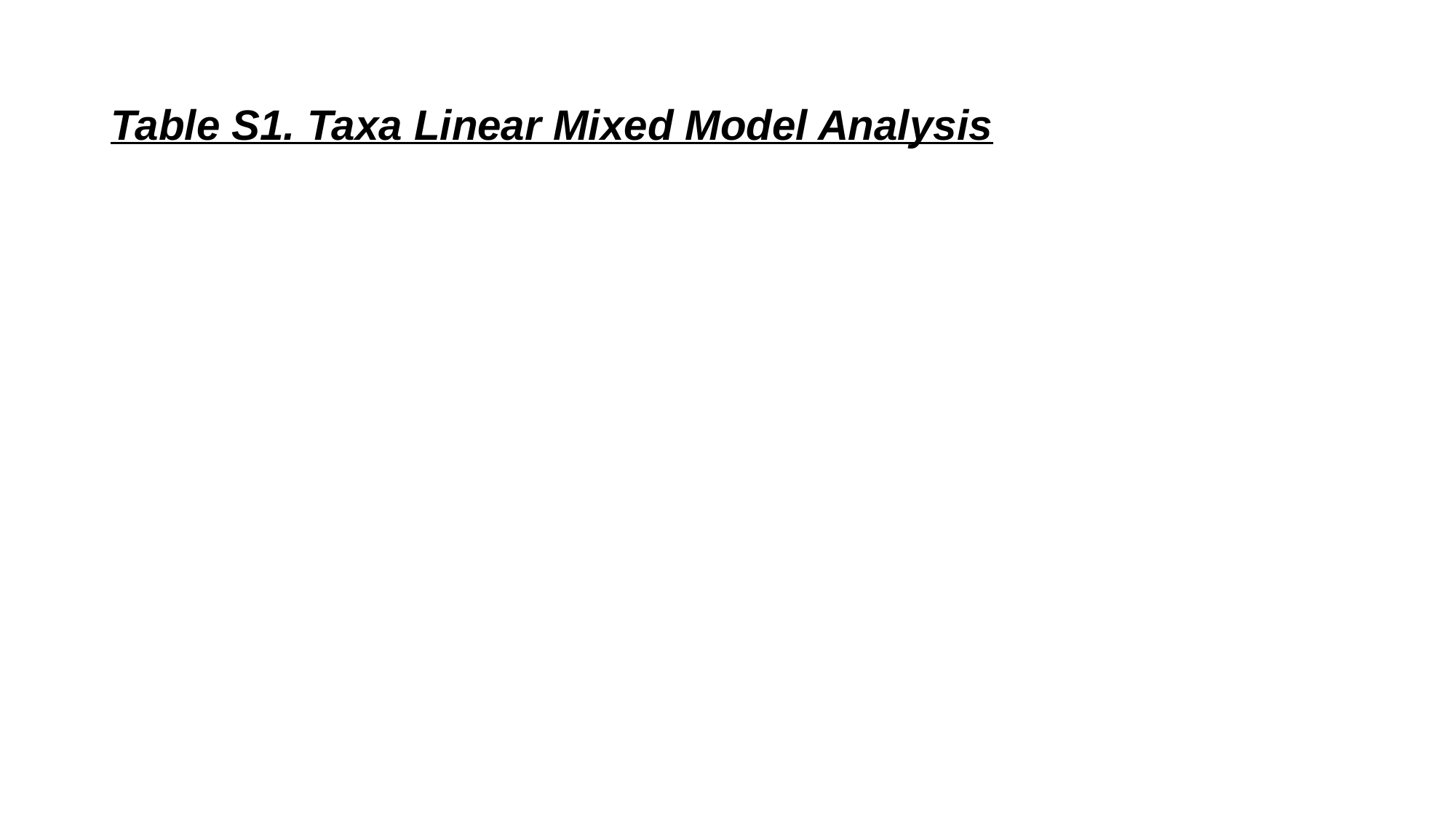

# Table S1. Taxa Linear Mixed Model Analysis

## Slide 14
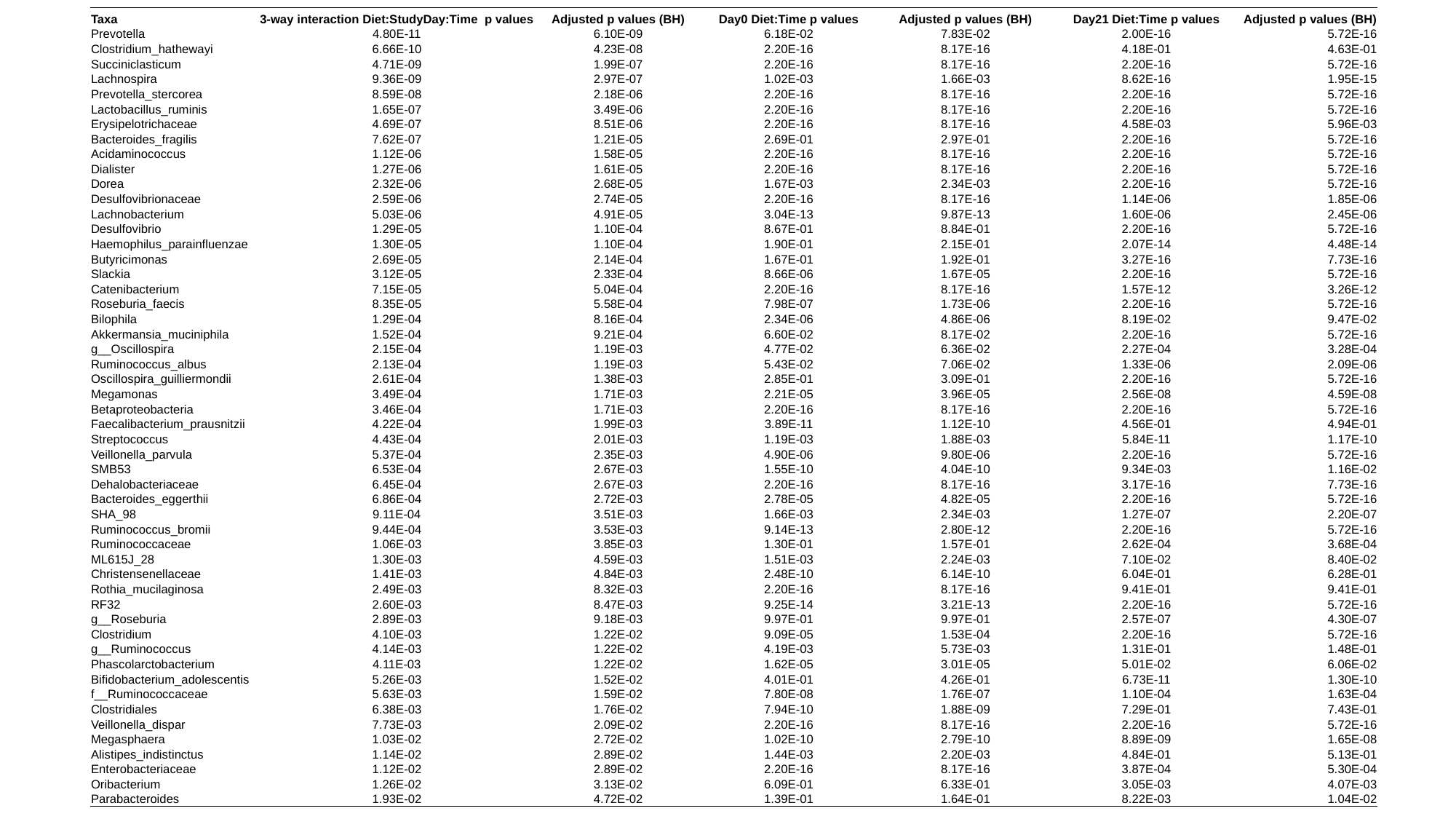

| Taxa | 3-way interaction Diet:StudyDay:Time p values | Adjusted p values (BH) | Day0 Diet:Time p values | Adjusted p values (BH) | Day21 Diet:Time p values | Adjusted p values (BH) |
| --- | --- | --- | --- | --- | --- | --- |
| Prevotella | 4.80E-11 | 6.10E-09 | 6.18E-02 | 7.83E-02 | 2.00E-16 | 5.72E-16 |
| Clostridium\_hathewayi | 6.66E-10 | 4.23E-08 | 2.20E-16 | 8.17E-16 | 4.18E-01 | 4.63E-01 |
| Succiniclasticum | 4.71E-09 | 1.99E-07 | 2.20E-16 | 8.17E-16 | 2.20E-16 | 5.72E-16 |
| Lachnospira | 9.36E-09 | 2.97E-07 | 1.02E-03 | 1.66E-03 | 8.62E-16 | 1.95E-15 |
| Prevotella\_stercorea | 8.59E-08 | 2.18E-06 | 2.20E-16 | 8.17E-16 | 2.20E-16 | 5.72E-16 |
| Lactobacillus\_ruminis | 1.65E-07 | 3.49E-06 | 2.20E-16 | 8.17E-16 | 2.20E-16 | 5.72E-16 |
| Erysipelotrichaceae | 4.69E-07 | 8.51E-06 | 2.20E-16 | 8.17E-16 | 4.58E-03 | 5.96E-03 |
| Bacteroides\_fragilis | 7.62E-07 | 1.21E-05 | 2.69E-01 | 2.97E-01 | 2.20E-16 | 5.72E-16 |
| Acidaminococcus | 1.12E-06 | 1.58E-05 | 2.20E-16 | 8.17E-16 | 2.20E-16 | 5.72E-16 |
| Dialister | 1.27E-06 | 1.61E-05 | 2.20E-16 | 8.17E-16 | 2.20E-16 | 5.72E-16 |
| Dorea | 2.32E-06 | 2.68E-05 | 1.67E-03 | 2.34E-03 | 2.20E-16 | 5.72E-16 |
| Desulfovibrionaceae | 2.59E-06 | 2.74E-05 | 2.20E-16 | 8.17E-16 | 1.14E-06 | 1.85E-06 |
| Lachnobacterium | 5.03E-06 | 4.91E-05 | 3.04E-13 | 9.87E-13 | 1.60E-06 | 2.45E-06 |
| Desulfovibrio | 1.29E-05 | 1.10E-04 | 8.67E-01 | 8.84E-01 | 2.20E-16 | 5.72E-16 |
| Haemophilus\_parainfluenzae | 1.30E-05 | 1.10E-04 | 1.90E-01 | 2.15E-01 | 2.07E-14 | 4.48E-14 |
| Butyricimonas | 2.69E-05 | 2.14E-04 | 1.67E-01 | 1.92E-01 | 3.27E-16 | 7.73E-16 |
| Slackia | 3.12E-05 | 2.33E-04 | 8.66E-06 | 1.67E-05 | 2.20E-16 | 5.72E-16 |
| Catenibacterium | 7.15E-05 | 5.04E-04 | 2.20E-16 | 8.17E-16 | 1.57E-12 | 3.26E-12 |
| Roseburia\_faecis | 8.35E-05 | 5.58E-04 | 7.98E-07 | 1.73E-06 | 2.20E-16 | 5.72E-16 |
| Bilophila | 1.29E-04 | 8.16E-04 | 2.34E-06 | 4.86E-06 | 8.19E-02 | 9.47E-02 |
| Akkermansia\_muciniphila | 1.52E-04 | 9.21E-04 | 6.60E-02 | 8.17E-02 | 2.20E-16 | 5.72E-16 |
| g\_\_Oscillospira | 2.15E-04 | 1.19E-03 | 4.77E-02 | 6.36E-02 | 2.27E-04 | 3.28E-04 |
| Ruminococcus\_albus | 2.13E-04 | 1.19E-03 | 5.43E-02 | 7.06E-02 | 1.33E-06 | 2.09E-06 |
| Oscillospira\_guilliermondii | 2.61E-04 | 1.38E-03 | 2.85E-01 | 3.09E-01 | 2.20E-16 | 5.72E-16 |
| Megamonas | 3.49E-04 | 1.71E-03 | 2.21E-05 | 3.96E-05 | 2.56E-08 | 4.59E-08 |
| Betaproteobacteria | 3.46E-04 | 1.71E-03 | 2.20E-16 | 8.17E-16 | 2.20E-16 | 5.72E-16 |
| Faecalibacterium\_prausnitzii | 4.22E-04 | 1.99E-03 | 3.89E-11 | 1.12E-10 | 4.56E-01 | 4.94E-01 |
| Streptococcus | 4.43E-04 | 2.01E-03 | 1.19E-03 | 1.88E-03 | 5.84E-11 | 1.17E-10 |
| Veillonella\_parvula | 5.37E-04 | 2.35E-03 | 4.90E-06 | 9.80E-06 | 2.20E-16 | 5.72E-16 |
| SMB53 | 6.53E-04 | 2.67E-03 | 1.55E-10 | 4.04E-10 | 9.34E-03 | 1.16E-02 |
| Dehalobacteriaceae | 6.45E-04 | 2.67E-03 | 2.20E-16 | 8.17E-16 | 3.17E-16 | 7.73E-16 |
| Bacteroides\_eggerthii | 6.86E-04 | 2.72E-03 | 2.78E-05 | 4.82E-05 | 2.20E-16 | 5.72E-16 |
| SHA\_98 | 9.11E-04 | 3.51E-03 | 1.66E-03 | 2.34E-03 | 1.27E-07 | 2.20E-07 |
| Ruminococcus\_bromii | 9.44E-04 | 3.53E-03 | 9.14E-13 | 2.80E-12 | 2.20E-16 | 5.72E-16 |
| Ruminococcaceae | 1.06E-03 | 3.85E-03 | 1.30E-01 | 1.57E-01 | 2.62E-04 | 3.68E-04 |
| ML615J\_28 | 1.30E-03 | 4.59E-03 | 1.51E-03 | 2.24E-03 | 7.10E-02 | 8.40E-02 |
| Christensenellaceae | 1.41E-03 | 4.84E-03 | 2.48E-10 | 6.14E-10 | 6.04E-01 | 6.28E-01 |
| Rothia\_mucilaginosa | 2.49E-03 | 8.32E-03 | 2.20E-16 | 8.17E-16 | 9.41E-01 | 9.41E-01 |
| RF32 | 2.60E-03 | 8.47E-03 | 9.25E-14 | 3.21E-13 | 2.20E-16 | 5.72E-16 |
| g\_\_Roseburia | 2.89E-03 | 9.18E-03 | 9.97E-01 | 9.97E-01 | 2.57E-07 | 4.30E-07 |
| Clostridium | 4.10E-03 | 1.22E-02 | 9.09E-05 | 1.53E-04 | 2.20E-16 | 5.72E-16 |
| g\_\_Ruminococcus | 4.14E-03 | 1.22E-02 | 4.19E-03 | 5.73E-03 | 1.31E-01 | 1.48E-01 |
| Phascolarctobacterium | 4.11E-03 | 1.22E-02 | 1.62E-05 | 3.01E-05 | 5.01E-02 | 6.06E-02 |
| Bifidobacterium\_adolescentis | 5.26E-03 | 1.52E-02 | 4.01E-01 | 4.26E-01 | 6.73E-11 | 1.30E-10 |
| f\_\_Ruminococcaceae | 5.63E-03 | 1.59E-02 | 7.80E-08 | 1.76E-07 | 1.10E-04 | 1.63E-04 |
| Clostridiales | 6.38E-03 | 1.76E-02 | 7.94E-10 | 1.88E-09 | 7.29E-01 | 7.43E-01 |
| Veillonella\_dispar | 7.73E-03 | 2.09E-02 | 2.20E-16 | 8.17E-16 | 2.20E-16 | 5.72E-16 |
| Megasphaera | 1.03E-02 | 2.72E-02 | 1.02E-10 | 2.79E-10 | 8.89E-09 | 1.65E-08 |
| Alistipes\_indistinctus | 1.14E-02 | 2.89E-02 | 1.44E-03 | 2.20E-03 | 4.84E-01 | 5.13E-01 |
| Enterobacteriaceae | 1.12E-02 | 2.89E-02 | 2.20E-16 | 8.17E-16 | 3.87E-04 | 5.30E-04 |
| Oribacterium | 1.26E-02 | 3.13E-02 | 6.09E-01 | 6.33E-01 | 3.05E-03 | 4.07E-03 |
| Parabacteroides | 1.93E-02 | 4.72E-02 | 1.39E-01 | 1.64E-01 | 8.22E-03 | 1.04E-02 |

## Slide 15
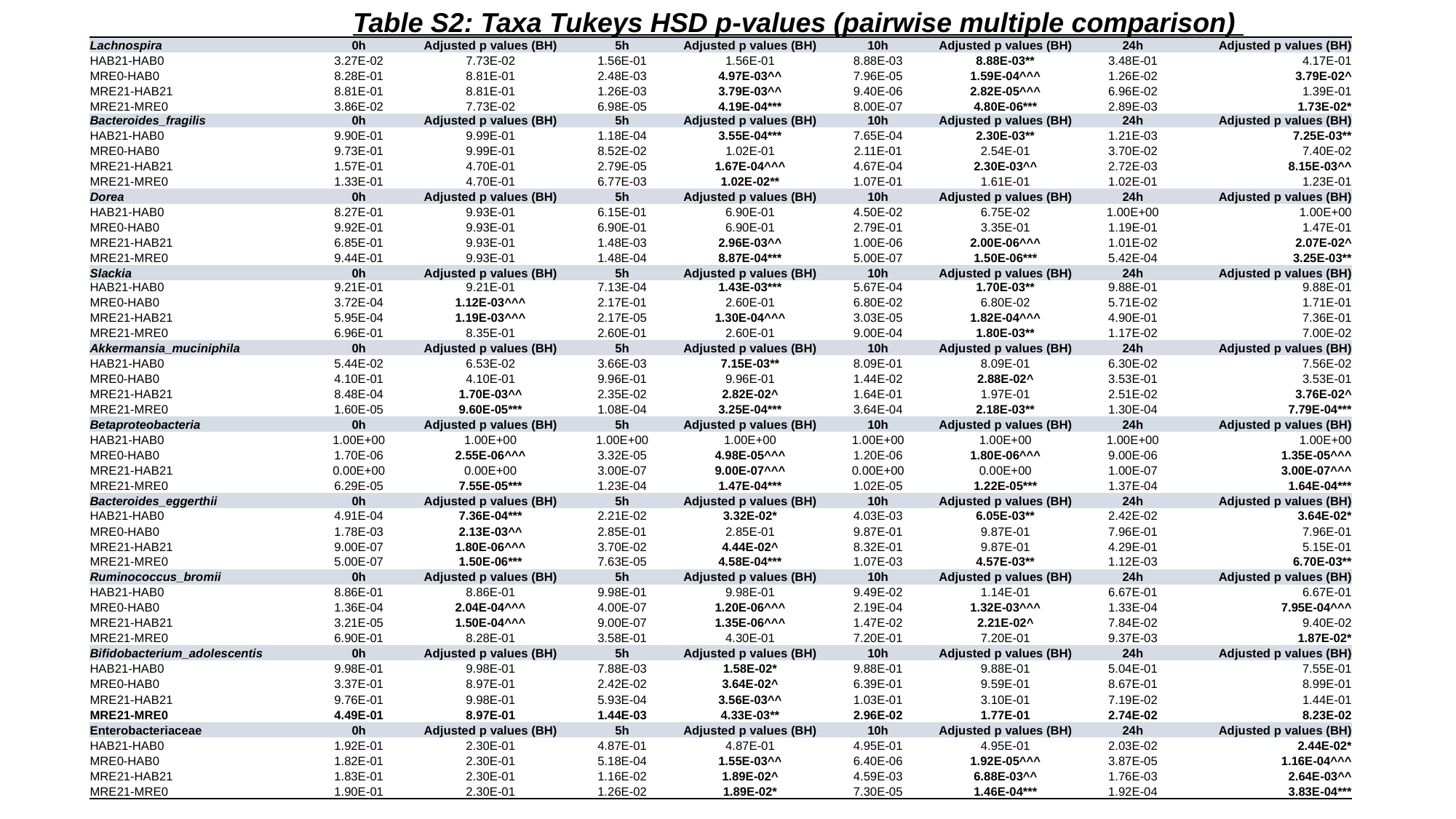

Table S2: Taxa Tukeys HSD p-values (pairwise multiple comparison)
| Lachnospira | 0h | Adjusted p values (BH) | 5h | Adjusted p values (BH) | 10h | Adjusted p values (BH) | 24h | Adjusted p values (BH) |
| --- | --- | --- | --- | --- | --- | --- | --- | --- |
| HAB21-HAB0 | 3.27E-02 | 7.73E-02 | 1.56E-01 | 1.56E-01 | 8.88E-03 | 8.88E-03\*\* | 3.48E-01 | 4.17E-01 |
| MRE0-HAB0 | 8.28E-01 | 8.81E-01 | 2.48E-03 | 4.97E-03^^ | 7.96E-05 | 1.59E-04^^^ | 1.26E-02 | 3.79E-02^ |
| MRE21-HAB21 | 8.81E-01 | 8.81E-01 | 1.26E-03 | 3.79E-03^^ | 9.40E-06 | 2.82E-05^^^ | 6.96E-02 | 1.39E-01 |
| MRE21-MRE0 | 3.86E-02 | 7.73E-02 | 6.98E-05 | 4.19E-04\*\*\* | 8.00E-07 | 4.80E-06\*\*\* | 2.89E-03 | 1.73E-02\* |
| Bacteroides\_fragilis | 0h | Adjusted p values (BH) | 5h | Adjusted p values (BH) | 10h | Adjusted p values (BH) | 24h | Adjusted p values (BH) |
| HAB21-HAB0 | 9.90E-01 | 9.99E-01 | 1.18E-04 | 3.55E-04\*\*\* | 7.65E-04 | 2.30E-03\*\* | 1.21E-03 | 7.25E-03\*\* |
| MRE0-HAB0 | 9.73E-01 | 9.99E-01 | 8.52E-02 | 1.02E-01 | 2.11E-01 | 2.54E-01 | 3.70E-02 | 7.40E-02 |
| MRE21-HAB21 | 1.57E-01 | 4.70E-01 | 2.79E-05 | 1.67E-04^^^ | 4.67E-04 | 2.30E-03^^ | 2.72E-03 | 8.15E-03^^ |
| MRE21-MRE0 | 1.33E-01 | 4.70E-01 | 6.77E-03 | 1.02E-02\*\* | 1.07E-01 | 1.61E-01 | 1.02E-01 | 1.23E-01 |
| Dorea | 0h | Adjusted p values (BH) | 5h | Adjusted p values (BH) | 10h | Adjusted p values (BH) | 24h | Adjusted p values (BH) |
| HAB21-HAB0 | 8.27E-01 | 9.93E-01 | 6.15E-01 | 6.90E-01 | 4.50E-02 | 6.75E-02 | 1.00E+00 | 1.00E+00 |
| MRE0-HAB0 | 9.92E-01 | 9.93E-01 | 6.90E-01 | 6.90E-01 | 2.79E-01 | 3.35E-01 | 1.19E-01 | 1.47E-01 |
| MRE21-HAB21 | 6.85E-01 | 9.93E-01 | 1.48E-03 | 2.96E-03^^ | 1.00E-06 | 2.00E-06^^^ | 1.01E-02 | 2.07E-02^ |
| MRE21-MRE0 | 9.44E-01 | 9.93E-01 | 1.48E-04 | 8.87E-04\*\*\* | 5.00E-07 | 1.50E-06\*\*\* | 5.42E-04 | 3.25E-03\*\* |
| Slackia | 0h | Adjusted p values (BH) | 5h | Adjusted p values (BH) | 10h | Adjusted p values (BH) | 24h | Adjusted p values (BH) |
| HAB21-HAB0 | 9.21E-01 | 9.21E-01 | 7.13E-04 | 1.43E-03\*\*\* | 5.67E-04 | 1.70E-03\*\* | 9.88E-01 | 9.88E-01 |
| MRE0-HAB0 | 3.72E-04 | 1.12E-03^^^ | 2.17E-01 | 2.60E-01 | 6.80E-02 | 6.80E-02 | 5.71E-02 | 1.71E-01 |
| MRE21-HAB21 | 5.95E-04 | 1.19E-03^^^ | 2.17E-05 | 1.30E-04^^^ | 3.03E-05 | 1.82E-04^^^ | 4.90E-01 | 7.36E-01 |
| MRE21-MRE0 | 6.96E-01 | 8.35E-01 | 2.60E-01 | 2.60E-01 | 9.00E-04 | 1.80E-03\*\* | 1.17E-02 | 7.00E-02 |
| Akkermansia\_muciniphila | 0h | Adjusted p values (BH) | 5h | Adjusted p values (BH) | 10h | Adjusted p values (BH) | 24h | Adjusted p values (BH) |
| HAB21-HAB0 | 5.44E-02 | 6.53E-02 | 3.66E-03 | 7.15E-03\*\* | 8.09E-01 | 8.09E-01 | 6.30E-02 | 7.56E-02 |
| MRE0-HAB0 | 4.10E-01 | 4.10E-01 | 9.96E-01 | 9.96E-01 | 1.44E-02 | 2.88E-02^ | 3.53E-01 | 3.53E-01 |
| MRE21-HAB21 | 8.48E-04 | 1.70E-03^^ | 2.35E-02 | 2.82E-02^ | 1.64E-01 | 1.97E-01 | 2.51E-02 | 3.76E-02^ |
| MRE21-MRE0 | 1.60E-05 | 9.60E-05\*\*\* | 1.08E-04 | 3.25E-04\*\*\* | 3.64E-04 | 2.18E-03\*\* | 1.30E-04 | 7.79E-04\*\*\* |
| Betaproteobacteria | 0h | Adjusted p values (BH) | 5h | Adjusted p values (BH) | 10h | Adjusted p values (BH) | 24h | Adjusted p values (BH) |
| HAB21-HAB0 | 1.00E+00 | 1.00E+00 | 1.00E+00 | 1.00E+00 | 1.00E+00 | 1.00E+00 | 1.00E+00 | 1.00E+00 |
| MRE0-HAB0 | 1.70E-06 | 2.55E-06^^^ | 3.32E-05 | 4.98E-05^^^ | 1.20E-06 | 1.80E-06^^^ | 9.00E-06 | 1.35E-05^^^ |
| MRE21-HAB21 | 0.00E+00 | 0.00E+00 | 3.00E-07 | 9.00E-07^^^ | 0.00E+00 | 0.00E+00 | 1.00E-07 | 3.00E-07^^^ |
| MRE21-MRE0 | 6.29E-05 | 7.55E-05\*\*\* | 1.23E-04 | 1.47E-04\*\*\* | 1.02E-05 | 1.22E-05\*\*\* | 1.37E-04 | 1.64E-04\*\*\* |
| Bacteroides\_eggerthii | 0h | Adjusted p values (BH) | 5h | Adjusted p values (BH) | 10h | Adjusted p values (BH) | 24h | Adjusted p values (BH) |
| HAB21-HAB0 | 4.91E-04 | 7.36E-04\*\*\* | 2.21E-02 | 3.32E-02\* | 4.03E-03 | 6.05E-03\*\* | 2.42E-02 | 3.64E-02\* |
| MRE0-HAB0 | 1.78E-03 | 2.13E-03^^ | 2.85E-01 | 2.85E-01 | 9.87E-01 | 9.87E-01 | 7.96E-01 | 7.96E-01 |
| MRE21-HAB21 | 9.00E-07 | 1.80E-06^^^ | 3.70E-02 | 4.44E-02^ | 8.32E-01 | 9.87E-01 | 4.29E-01 | 5.15E-01 |
| MRE21-MRE0 | 5.00E-07 | 1.50E-06\*\*\* | 7.63E-05 | 4.58E-04\*\*\* | 1.07E-03 | 4.57E-03\*\* | 1.12E-03 | 6.70E-03\*\* |
| Ruminococcus\_bromii | 0h | Adjusted p values (BH) | 5h | Adjusted p values (BH) | 10h | Adjusted p values (BH) | 24h | Adjusted p values (BH) |
| HAB21-HAB0 | 8.86E-01 | 8.86E-01 | 9.98E-01 | 9.98E-01 | 9.49E-02 | 1.14E-01 | 6.67E-01 | 6.67E-01 |
| MRE0-HAB0 | 1.36E-04 | 2.04E-04^^^ | 4.00E-07 | 1.20E-06^^^ | 2.19E-04 | 1.32E-03^^^ | 1.33E-04 | 7.95E-04^^^ |
| MRE21-HAB21 | 3.21E-05 | 1.50E-04^^^ | 9.00E-07 | 1.35E-06^^^ | 1.47E-02 | 2.21E-02^ | 7.84E-02 | 9.40E-02 |
| MRE21-MRE0 | 6.90E-01 | 8.28E-01 | 3.58E-01 | 4.30E-01 | 7.20E-01 | 7.20E-01 | 9.37E-03 | 1.87E-02\* |
| Bifidobacterium\_adolescentis | 0h | Adjusted p values (BH) | 5h | Adjusted p values (BH) | 10h | Adjusted p values (BH) | 24h | Adjusted p values (BH) |
| HAB21-HAB0 | 9.98E-01 | 9.98E-01 | 7.88E-03 | 1.58E-02\* | 9.88E-01 | 9.88E-01 | 5.04E-01 | 7.55E-01 |
| MRE0-HAB0 | 3.37E-01 | 8.97E-01 | 2.42E-02 | 3.64E-02^ | 6.39E-01 | 9.59E-01 | 8.67E-01 | 8.99E-01 |
| MRE21-HAB21 | 9.76E-01 | 9.98E-01 | 5.93E-04 | 3.56E-03^^ | 1.03E-01 | 3.10E-01 | 7.19E-02 | 1.44E-01 |
| MRE21-MRE0 | 4.49E-01 | 8.97E-01 | 1.44E-03 | 4.33E-03\*\* | 2.96E-02 | 1.77E-01 | 2.74E-02 | 8.23E-02 |
| Enterobacteriaceae | 0h | Adjusted p values (BH) | 5h | Adjusted p values (BH) | 10h | Adjusted p values (BH) | 24h | Adjusted p values (BH) |
| HAB21-HAB0 | 1.92E-01 | 2.30E-01 | 4.87E-01 | 4.87E-01 | 4.95E-01 | 4.95E-01 | 2.03E-02 | 2.44E-02\* |
| MRE0-HAB0 | 1.82E-01 | 2.30E-01 | 5.18E-04 | 1.55E-03^^ | 6.40E-06 | 1.92E-05^^^ | 3.87E-05 | 1.16E-04^^^ |
| MRE21-HAB21 | 1.83E-01 | 2.30E-01 | 1.16E-02 | 1.89E-02^ | 4.59E-03 | 6.88E-03^^ | 1.76E-03 | 2.64E-03^^ |
| MRE21-MRE0 | 1.90E-01 | 2.30E-01 | 1.26E-02 | 1.89E-02\* | 7.30E-05 | 1.46E-04\*\*\* | 1.92E-04 | 3.83E-04\*\*\* |

## Slide 16
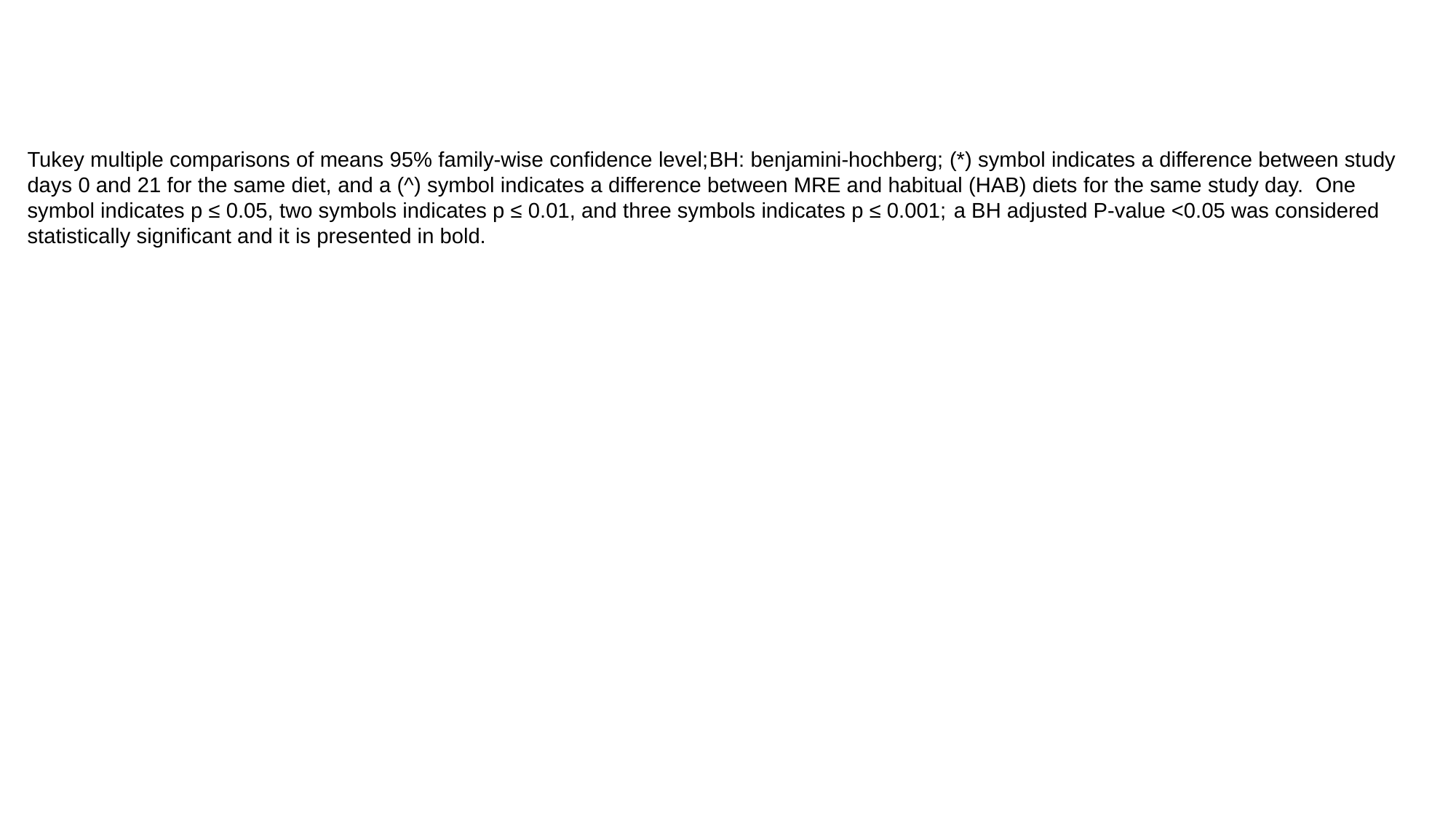

Tukey multiple comparisons of means 95% family-wise confidence level;BH: benjamini-hochberg; (*) symbol indicates a difference between study days 0 and 21 for the same diet, and a (^) symbol indicates a difference between MRE and habitual (HAB) diets for the same study day. One symbol indicates p ≤ 0.05, two symbols indicates p ≤ 0.01, and three symbols indicates p ≤ 0.001; a BH adjusted P-value <0.05 was considered statistically significant and it is presented in bold.

## Slide 17
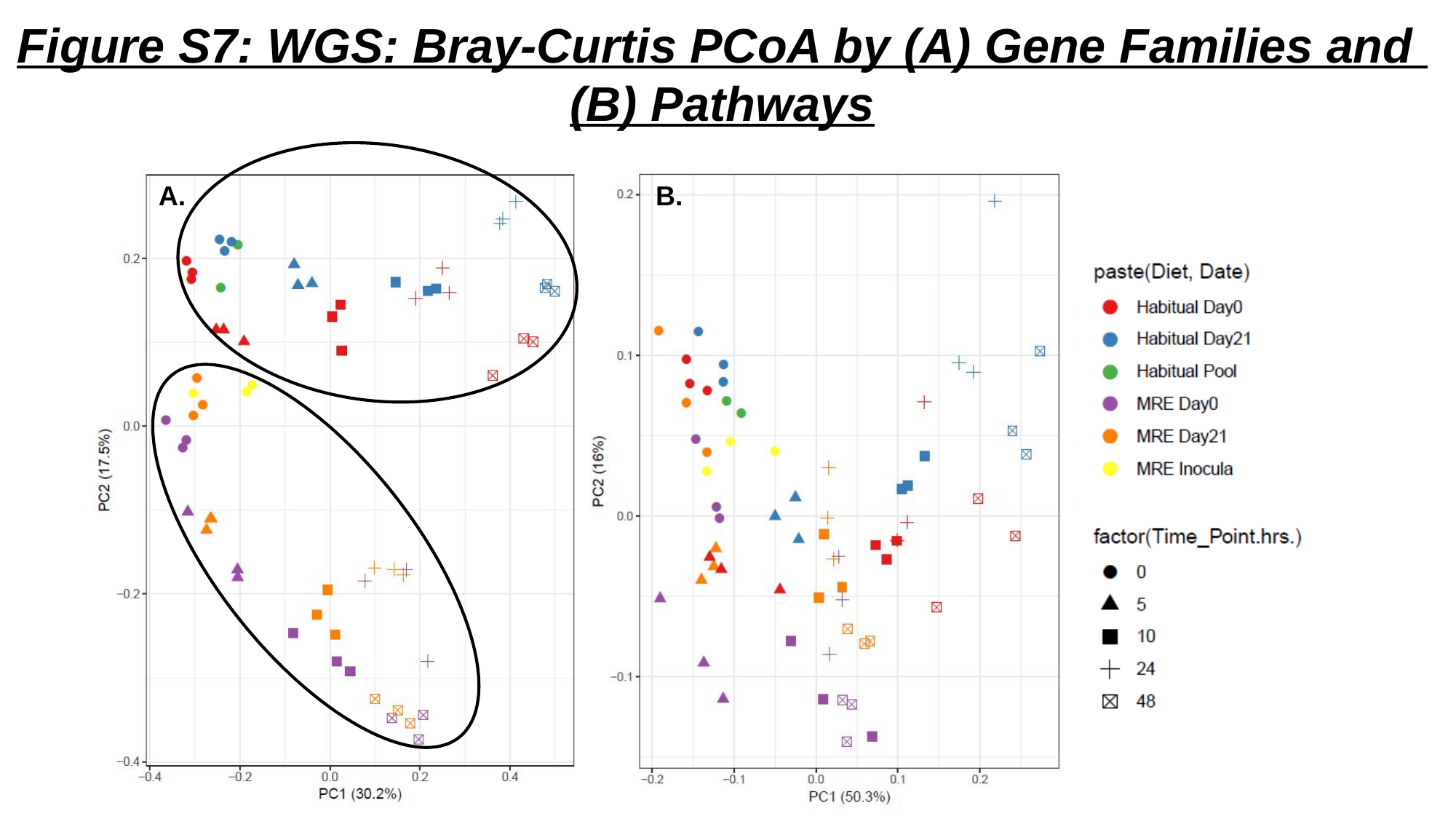

Figure S7: WGS: Bray-Curtis PCoA by (A) Gene Families and
(B) Pathways
A.
B.

## Slide 18
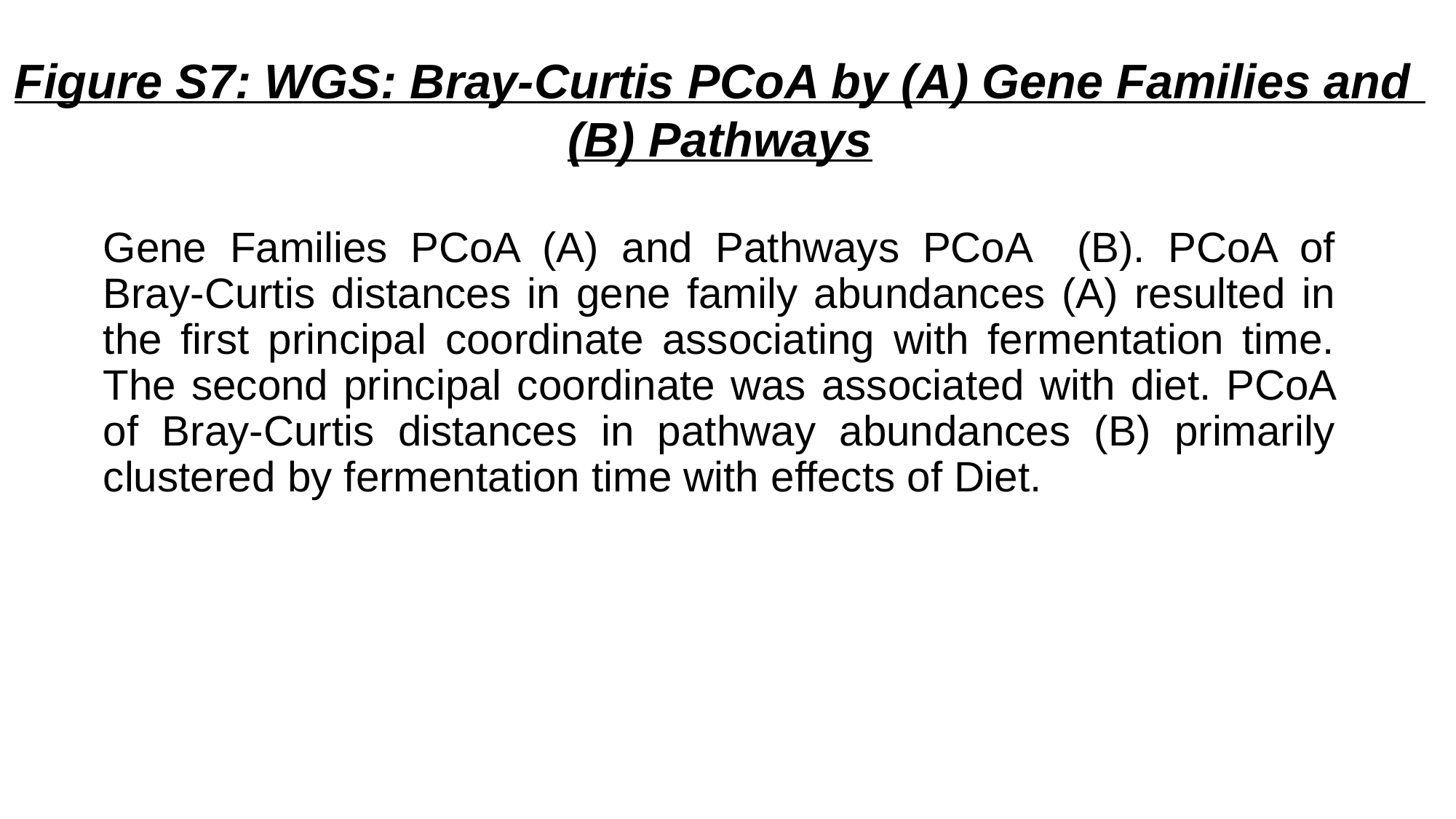

Figure S7: WGS: Bray-Curtis PCoA by (A) Gene Families and
(B) Pathways
Gene Families PCoA (A) and Pathways PCoA (B). PCoA of Bray-Curtis distances in gene family abundances (A) resulted in the first principal coordinate associating with fermentation time. The second principal coordinate was associated with diet. PCoA of Bray-Curtis distances in pathway abundances (B) primarily clustered by fermentation time with effects of Diet.

## Slide 19
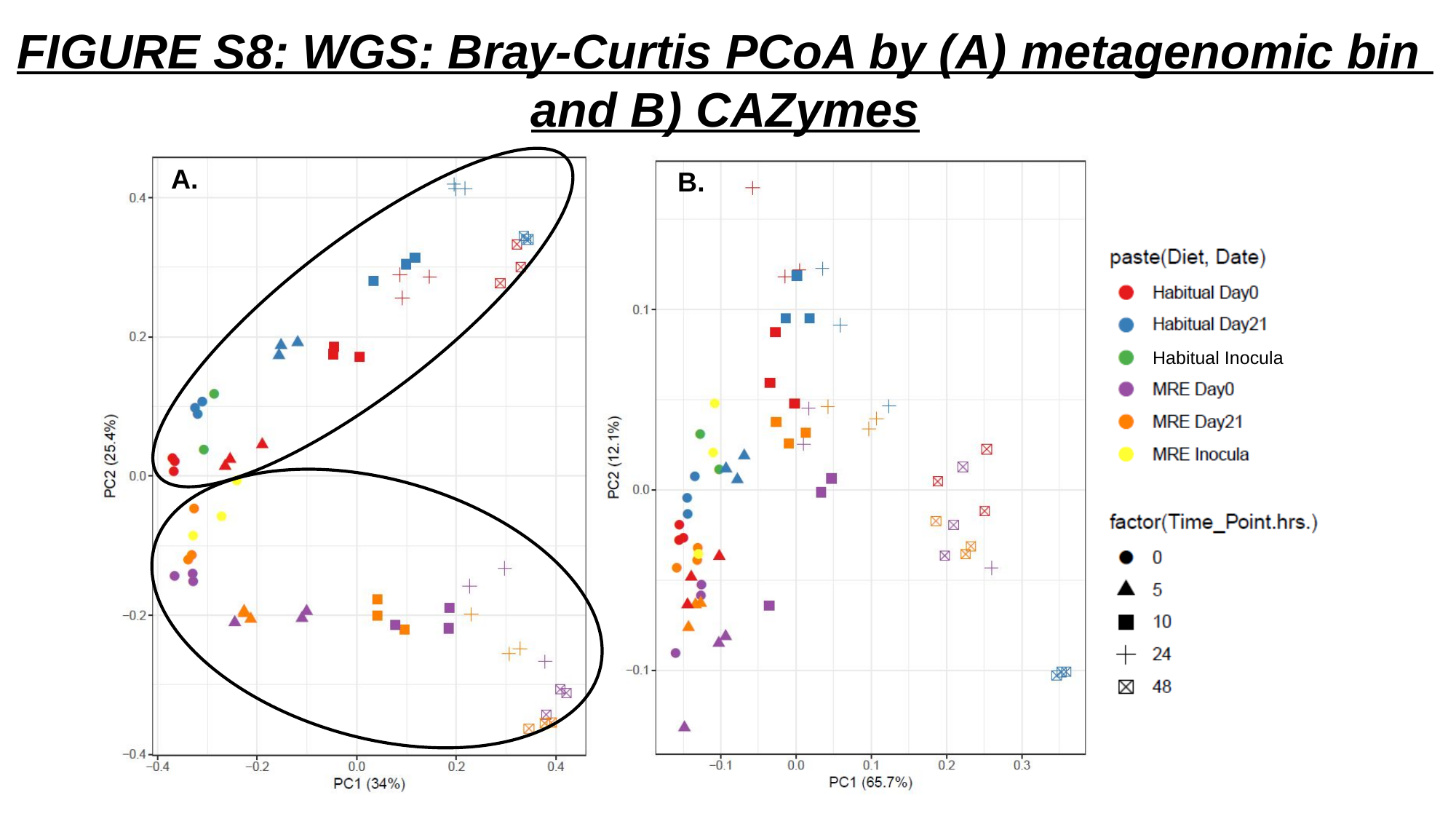

FIGURE S8: WGS: Bray-Curtis PCoA by (A) metagenomic bin
and B) CAZymes
A.
B.
Habitual Inocula

## Slide 20
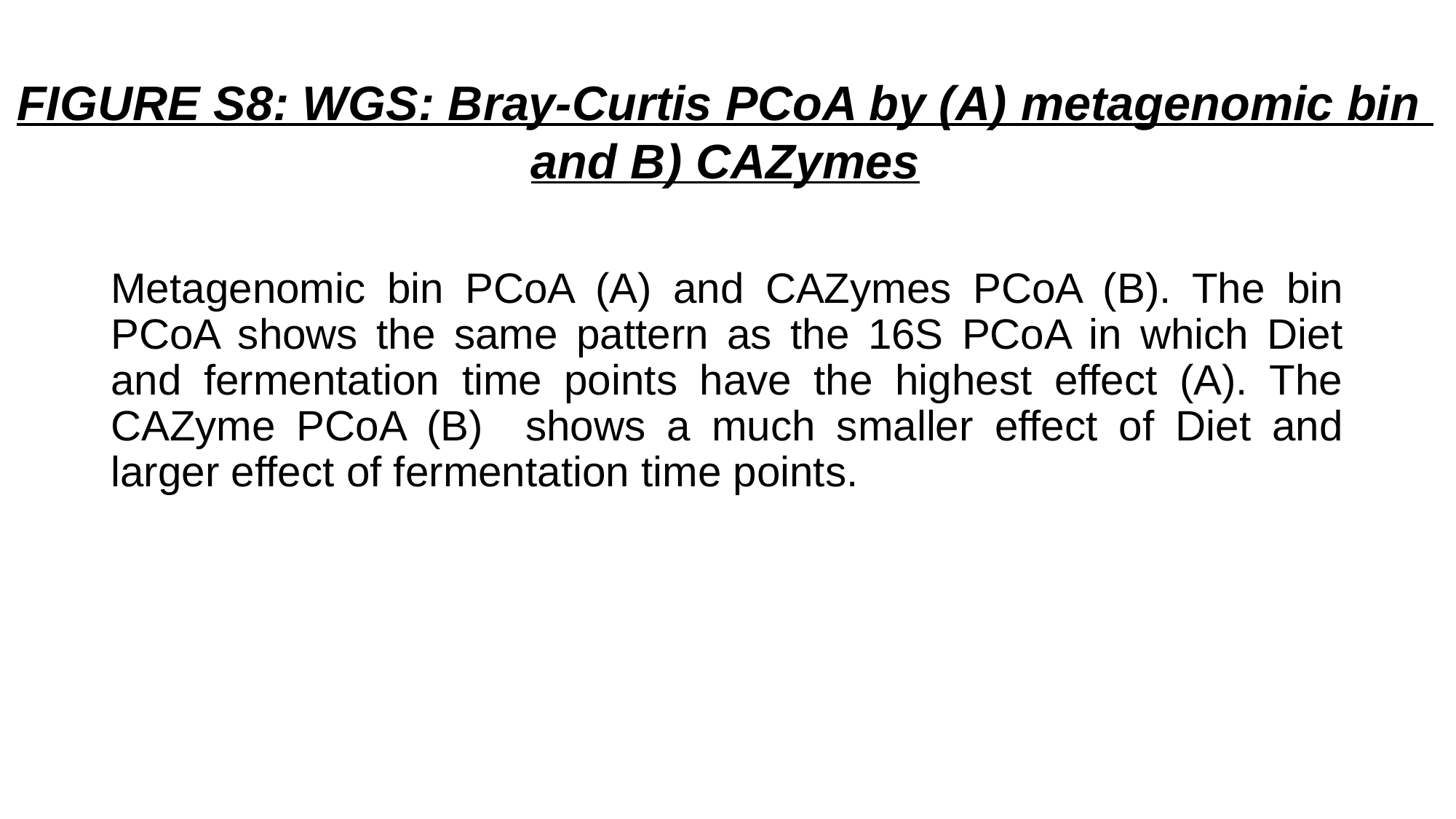

FIGURE S8: WGS: Bray-Curtis PCoA by (A) metagenomic bin
and B) CAZymes
Metagenomic bin PCoA (A) and CAZymes PCoA (B). The bin PCoA shows the same pattern as the 16S PCoA in which Diet and fermentation time points have the highest effect (A). The CAZyme PCoA (B) shows a much smaller effect of Diet and larger effect of fermentation time points.

## Slide 21
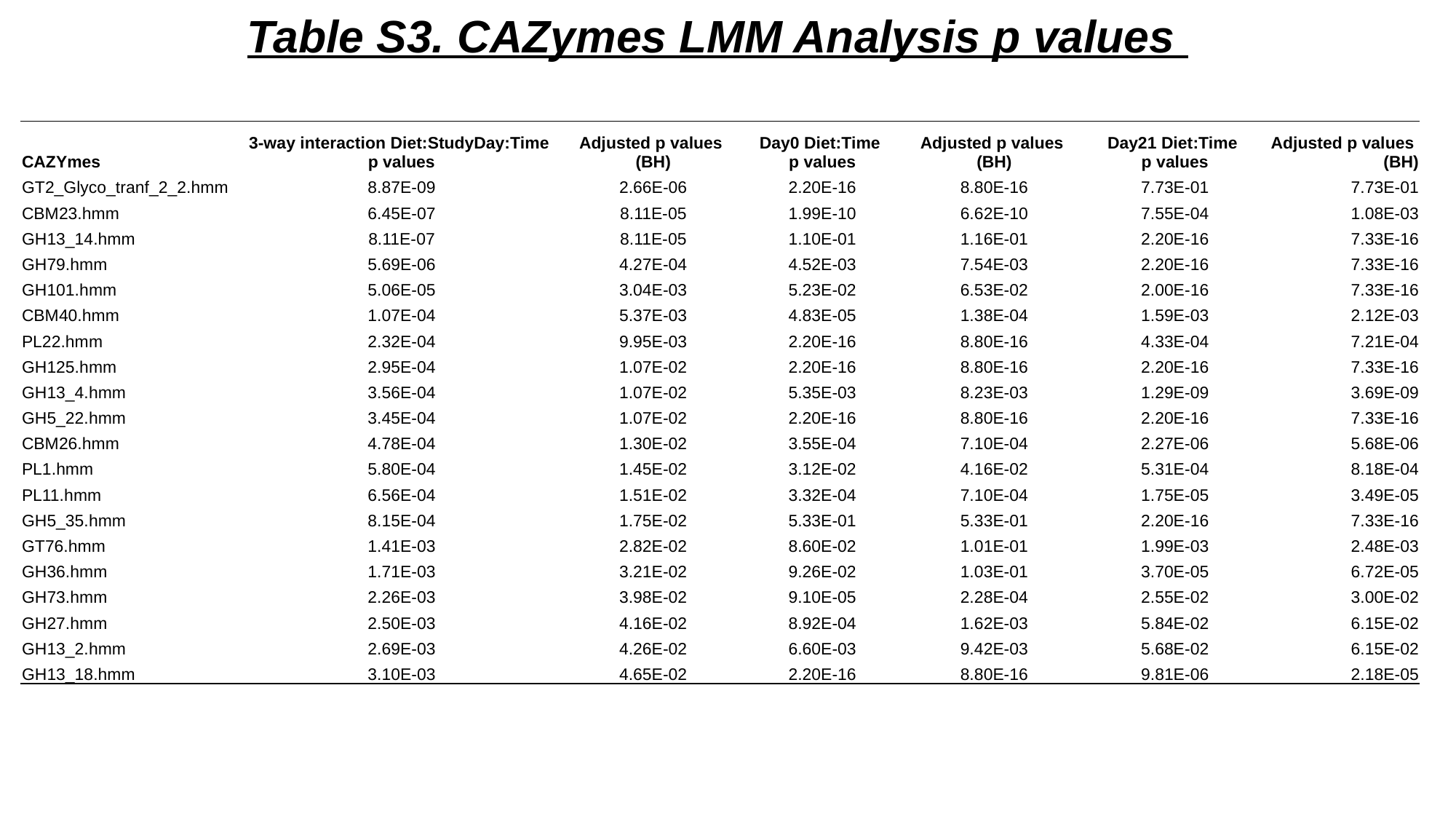

Table S3. CAZymes LMM Analysis p values
| CAZYmes | 3-way interaction Diet:StudyDay:Time p values | Adjusted p values (BH) | Day0 Diet:Time p values | Adjusted p values (BH) | Day21 Diet:Time p values | Adjusted p values (BH) |
| --- | --- | --- | --- | --- | --- | --- |
| GT2\_Glyco\_tranf\_2\_2.hmm | 8.87E-09 | 2.66E-06 | 2.20E-16 | 8.80E-16 | 7.73E-01 | 7.73E-01 |
| CBM23.hmm | 6.45E-07 | 8.11E-05 | 1.99E-10 | 6.62E-10 | 7.55E-04 | 1.08E-03 |
| GH13\_14.hmm | 8.11E-07 | 8.11E-05 | 1.10E-01 | 1.16E-01 | 2.20E-16 | 7.33E-16 |
| GH79.hmm | 5.69E-06 | 4.27E-04 | 4.52E-03 | 7.54E-03 | 2.20E-16 | 7.33E-16 |
| GH101.hmm | 5.06E-05 | 3.04E-03 | 5.23E-02 | 6.53E-02 | 2.00E-16 | 7.33E-16 |
| CBM40.hmm | 1.07E-04 | 5.37E-03 | 4.83E-05 | 1.38E-04 | 1.59E-03 | 2.12E-03 |
| PL22.hmm | 2.32E-04 | 9.95E-03 | 2.20E-16 | 8.80E-16 | 4.33E-04 | 7.21E-04 |
| GH125.hmm | 2.95E-04 | 1.07E-02 | 2.20E-16 | 8.80E-16 | 2.20E-16 | 7.33E-16 |
| GH13\_4.hmm | 3.56E-04 | 1.07E-02 | 5.35E-03 | 8.23E-03 | 1.29E-09 | 3.69E-09 |
| GH5\_22.hmm | 3.45E-04 | 1.07E-02 | 2.20E-16 | 8.80E-16 | 2.20E-16 | 7.33E-16 |
| CBM26.hmm | 4.78E-04 | 1.30E-02 | 3.55E-04 | 7.10E-04 | 2.27E-06 | 5.68E-06 |
| PL1.hmm | 5.80E-04 | 1.45E-02 | 3.12E-02 | 4.16E-02 | 5.31E-04 | 8.18E-04 |
| PL11.hmm | 6.56E-04 | 1.51E-02 | 3.32E-04 | 7.10E-04 | 1.75E-05 | 3.49E-05 |
| GH5\_35.hmm | 8.15E-04 | 1.75E-02 | 5.33E-01 | 5.33E-01 | 2.20E-16 | 7.33E-16 |
| GT76.hmm | 1.41E-03 | 2.82E-02 | 8.60E-02 | 1.01E-01 | 1.99E-03 | 2.48E-03 |
| GH36.hmm | 1.71E-03 | 3.21E-02 | 9.26E-02 | 1.03E-01 | 3.70E-05 | 6.72E-05 |
| GH73.hmm | 2.26E-03 | 3.98E-02 | 9.10E-05 | 2.28E-04 | 2.55E-02 | 3.00E-02 |
| GH27.hmm | 2.50E-03 | 4.16E-02 | 8.92E-04 | 1.62E-03 | 5.84E-02 | 6.15E-02 |
| GH13\_2.hmm | 2.69E-03 | 4.26E-02 | 6.60E-03 | 9.42E-03 | 5.68E-02 | 6.15E-02 |
| GH13\_18.hmm | 3.10E-03 | 4.65E-02 | 2.20E-16 | 8.80E-16 | 9.81E-06 | 2.18E-05 |

## Slide 22
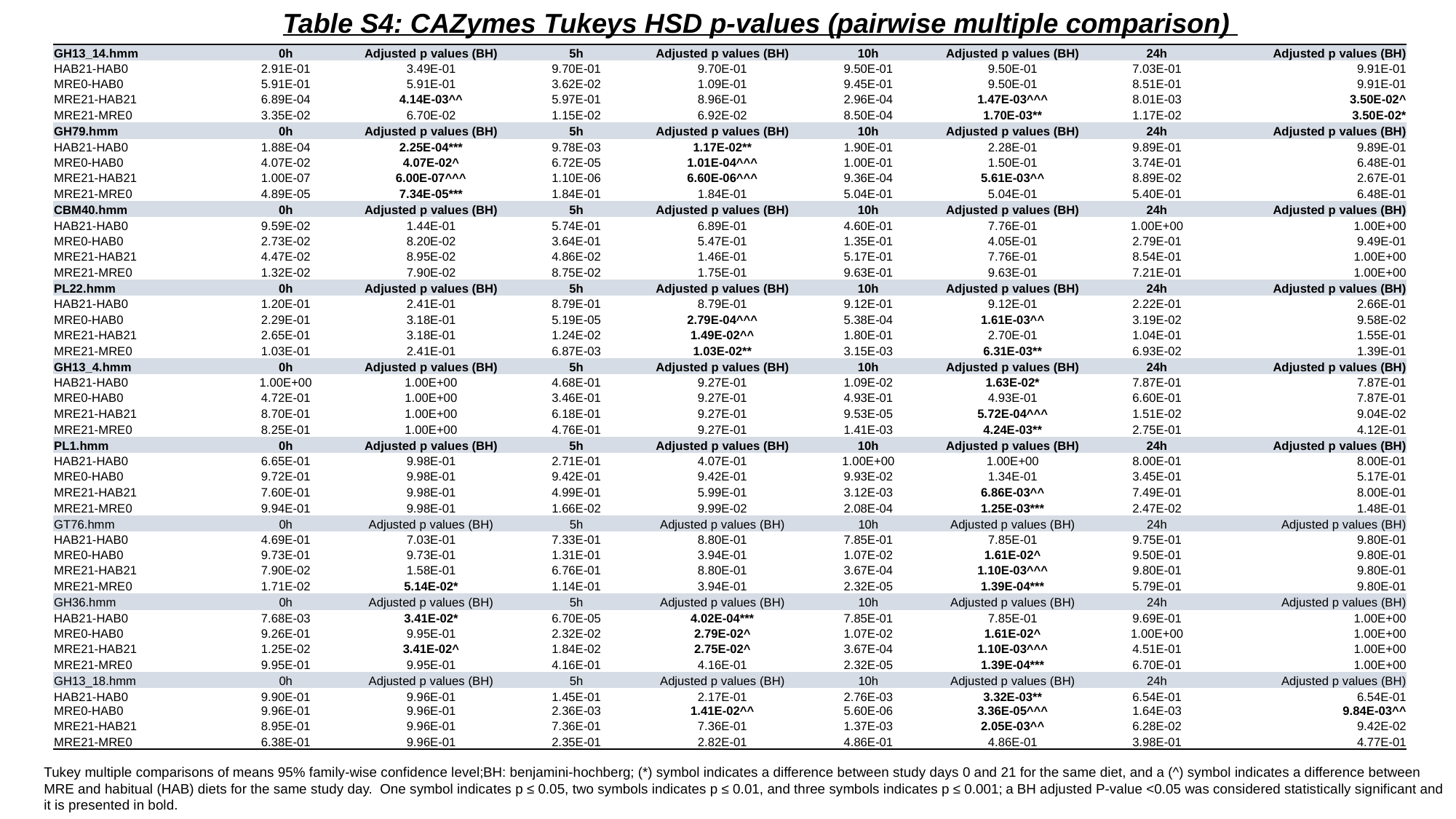

Table S4: CAZymes Tukeys HSD p-values (pairwise multiple comparison)
| GH13\_14.hmm | 0h | Adjusted p values (BH) | 5h | Adjusted p values (BH) | 10h | Adjusted p values (BH) | 24h | Adjusted p values (BH) |
| --- | --- | --- | --- | --- | --- | --- | --- | --- |
| HAB21-HAB0 | 2.91E-01 | 3.49E-01 | 9.70E-01 | 9.70E-01 | 9.50E-01 | 9.50E-01 | 7.03E-01 | 9.91E-01 |
| MRE0-HAB0 | 5.91E-01 | 5.91E-01 | 3.62E-02 | 1.09E-01 | 9.45E-01 | 9.50E-01 | 8.51E-01 | 9.91E-01 |
| MRE21-HAB21 | 6.89E-04 | 4.14E-03^^ | 5.97E-01 | 8.96E-01 | 2.96E-04 | 1.47E-03^^^ | 8.01E-03 | 3.50E-02^ |
| MRE21-MRE0 | 3.35E-02 | 6.70E-02 | 1.15E-02 | 6.92E-02 | 8.50E-04 | 1.70E-03\*\* | 1.17E-02 | 3.50E-02\* |
| GH79.hmm | 0h | Adjusted p values (BH) | 5h | Adjusted p values (BH) | 10h | Adjusted p values (BH) | 24h | Adjusted p values (BH) |
| HAB21-HAB0 | 1.88E-04 | 2.25E-04\*\*\* | 9.78E-03 | 1.17E-02\*\* | 1.90E-01 | 2.28E-01 | 9.89E-01 | 9.89E-01 |
| MRE0-HAB0 | 4.07E-02 | 4.07E-02^ | 6.72E-05 | 1.01E-04^^^ | 1.00E-01 | 1.50E-01 | 3.74E-01 | 6.48E-01 |
| MRE21-HAB21 | 1.00E-07 | 6.00E-07^^^ | 1.10E-06 | 6.60E-06^^^ | 9.36E-04 | 5.61E-03^^ | 8.89E-02 | 2.67E-01 |
| MRE21-MRE0 | 4.89E-05 | 7.34E-05\*\*\* | 1.84E-01 | 1.84E-01 | 5.04E-01 | 5.04E-01 | 5.40E-01 | 6.48E-01 |
| CBM40.hmm | 0h | Adjusted p values (BH) | 5h | Adjusted p values (BH) | 10h | Adjusted p values (BH) | 24h | Adjusted p values (BH) |
| HAB21-HAB0 | 9.59E-02 | 1.44E-01 | 5.74E-01 | 6.89E-01 | 4.60E-01 | 7.76E-01 | 1.00E+00 | 1.00E+00 |
| MRE0-HAB0 | 2.73E-02 | 8.20E-02 | 3.64E-01 | 5.47E-01 | 1.35E-01 | 4.05E-01 | 2.79E-01 | 9.49E-01 |
| MRE21-HAB21 | 4.47E-02 | 8.95E-02 | 4.86E-02 | 1.46E-01 | 5.17E-01 | 7.76E-01 | 8.54E-01 | 1.00E+00 |
| MRE21-MRE0 | 1.32E-02 | 7.90E-02 | 8.75E-02 | 1.75E-01 | 9.63E-01 | 9.63E-01 | 7.21E-01 | 1.00E+00 |
| PL22.hmm | 0h | Adjusted p values (BH) | 5h | Adjusted p values (BH) | 10h | Adjusted p values (BH) | 24h | Adjusted p values (BH) |
| HAB21-HAB0 | 1.20E-01 | 2.41E-01 | 8.79E-01 | 8.79E-01 | 9.12E-01 | 9.12E-01 | 2.22E-01 | 2.66E-01 |
| MRE0-HAB0 | 2.29E-01 | 3.18E-01 | 5.19E-05 | 2.79E-04^^^ | 5.38E-04 | 1.61E-03^^ | 3.19E-02 | 9.58E-02 |
| MRE21-HAB21 | 2.65E-01 | 3.18E-01 | 1.24E-02 | 1.49E-02^^ | 1.80E-01 | 2.70E-01 | 1.04E-01 | 1.55E-01 |
| MRE21-MRE0 | 1.03E-01 | 2.41E-01 | 6.87E-03 | 1.03E-02\*\* | 3.15E-03 | 6.31E-03\*\* | 6.93E-02 | 1.39E-01 |
| GH13\_4.hmm | 0h | Adjusted p values (BH) | 5h | Adjusted p values (BH) | 10h | Adjusted p values (BH) | 24h | Adjusted p values (BH) |
| HAB21-HAB0 | 1.00E+00 | 1.00E+00 | 4.68E-01 | 9.27E-01 | 1.09E-02 | 1.63E-02\* | 7.87E-01 | 7.87E-01 |
| MRE0-HAB0 | 4.72E-01 | 1.00E+00 | 3.46E-01 | 9.27E-01 | 4.93E-01 | 4.93E-01 | 6.60E-01 | 7.87E-01 |
| MRE21-HAB21 | 8.70E-01 | 1.00E+00 | 6.18E-01 | 9.27E-01 | 9.53E-05 | 5.72E-04^^^ | 1.51E-02 | 9.04E-02 |
| MRE21-MRE0 | 8.25E-01 | 1.00E+00 | 4.76E-01 | 9.27E-01 | 1.41E-03 | 4.24E-03\*\* | 2.75E-01 | 4.12E-01 |
| PL1.hmm | 0h | Adjusted p values (BH) | 5h | Adjusted p values (BH) | 10h | Adjusted p values (BH) | 24h | Adjusted p values (BH) |
| HAB21-HAB0 | 6.65E-01 | 9.98E-01 | 2.71E-01 | 4.07E-01 | 1.00E+00 | 1.00E+00 | 8.00E-01 | 8.00E-01 |
| MRE0-HAB0 | 9.72E-01 | 9.98E-01 | 9.42E-01 | 9.42E-01 | 9.93E-02 | 1.34E-01 | 3.45E-01 | 5.17E-01 |
| MRE21-HAB21 | 7.60E-01 | 9.98E-01 | 4.99E-01 | 5.99E-01 | 3.12E-03 | 6.86E-03^^ | 7.49E-01 | 8.00E-01 |
| MRE21-MRE0 | 9.94E-01 | 9.98E-01 | 1.66E-02 | 9.99E-02 | 2.08E-04 | 1.25E-03\*\*\* | 2.47E-02 | 1.48E-01 |
| GT76.hmm | 0h | Adjusted p values (BH) | 5h | Adjusted p values (BH) | 10h | Adjusted p values (BH) | 24h | Adjusted p values (BH) |
| HAB21-HAB0 | 4.69E-01 | 7.03E-01 | 7.33E-01 | 8.80E-01 | 7.85E-01 | 7.85E-01 | 9.75E-01 | 9.80E-01 |
| MRE0-HAB0 | 9.73E-01 | 9.73E-01 | 1.31E-01 | 3.94E-01 | 1.07E-02 | 1.61E-02^ | 9.50E-01 | 9.80E-01 |
| MRE21-HAB21 | 7.90E-02 | 1.58E-01 | 6.76E-01 | 8.80E-01 | 3.67E-04 | 1.10E-03^^^ | 9.80E-01 | 9.80E-01 |
| MRE21-MRE0 | 1.71E-02 | 5.14E-02\* | 1.14E-01 | 3.94E-01 | 2.32E-05 | 1.39E-04\*\*\* | 5.79E-01 | 9.80E-01 |
| GH36.hmm | 0h | Adjusted p values (BH) | 5h | Adjusted p values (BH) | 10h | Adjusted p values (BH) | 24h | Adjusted p values (BH) |
| HAB21-HAB0 | 7.68E-03 | 3.41E-02\* | 6.70E-05 | 4.02E-04\*\*\* | 7.85E-01 | 7.85E-01 | 9.69E-01 | 1.00E+00 |
| MRE0-HAB0 | 9.26E-01 | 9.95E-01 | 2.32E-02 | 2.79E-02^ | 1.07E-02 | 1.61E-02^ | 1.00E+00 | 1.00E+00 |
| MRE21-HAB21 | 1.25E-02 | 3.41E-02^ | 1.84E-02 | 2.75E-02^ | 3.67E-04 | 1.10E-03^^^ | 4.51E-01 | 1.00E+00 |
| MRE21-MRE0 | 9.95E-01 | 9.95E-01 | 4.16E-01 | 4.16E-01 | 2.32E-05 | 1.39E-04\*\*\* | 6.70E-01 | 1.00E+00 |
| GH13\_18.hmm | 0h | Adjusted p values (BH) | 5h | Adjusted p values (BH) | 10h | Adjusted p values (BH) | 24h | Adjusted p values (BH) |
| HAB21-HAB0 | 9.90E-01 | 9.96E-01 | 1.45E-01 | 2.17E-01 | 2.76E-03 | 3.32E-03\*\* | 6.54E-01 | 6.54E-01 |
| MRE0-HAB0 | 9.96E-01 | 9.96E-01 | 2.36E-03 | 1.41E-02^^ | 5.60E-06 | 3.36E-05^^^ | 1.64E-03 | 9.84E-03^^ |
| MRE21-HAB21 | 8.95E-01 | 9.96E-01 | 7.36E-01 | 7.36E-01 | 1.37E-03 | 2.05E-03^^ | 6.28E-02 | 9.42E-02 |
| MRE21-MRE0 | 6.38E-01 | 9.96E-01 | 2.35E-01 | 2.82E-01 | 4.86E-01 | 4.86E-01 | 3.98E-01 | 4.77E-01 |
Tukey multiple comparisons of means 95% family-wise confidence level;BH: benjamini-hochberg; (*) symbol indicates a difference between study days 0 and 21 for the same diet, and a (^) symbol indicates a difference between MRE and habitual (HAB) diets for the same study day. One symbol indicates p ≤ 0.05, two symbols indicates p ≤ 0.01, and three symbols indicates p ≤ 0.001; a BH adjusted P-value <0.05 was considered statistically significant and it is presented in bold.

## Slide 23
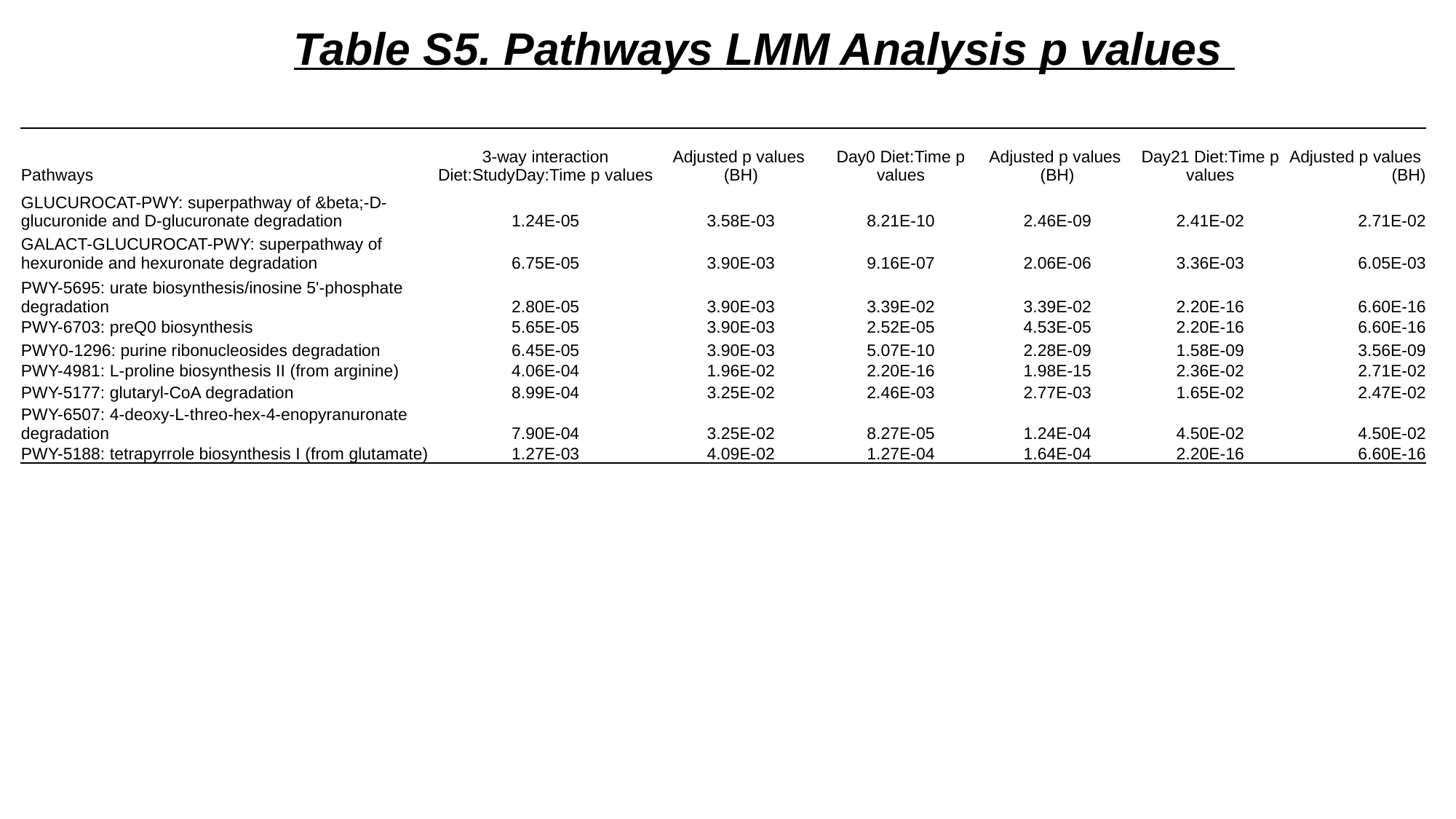

Table S5. Pathways LMM Analysis p values
| Pathways | 3-way interaction Diet:StudyDay:Time p values | Adjusted p values (BH) | Day0 Diet:Time p values | Adjusted p values (BH) | Day21 Diet:Time p values | Adjusted p values (BH) |
| --- | --- | --- | --- | --- | --- | --- |
| GLUCUROCAT-PWY: superpathway of &beta;-D-glucuronide and D-glucuronate degradation | 1.24E-05 | 3.58E-03 | 8.21E-10 | 2.46E-09 | 2.41E-02 | 2.71E-02 |
| GALACT-GLUCUROCAT-PWY: superpathway of hexuronide and hexuronate degradation | 6.75E-05 | 3.90E-03 | 9.16E-07 | 2.06E-06 | 3.36E-03 | 6.05E-03 |
| PWY-5695: urate biosynthesis/inosine 5'-phosphate degradation | 2.80E-05 | 3.90E-03 | 3.39E-02 | 3.39E-02 | 2.20E-16 | 6.60E-16 |
| PWY-6703: preQ0 biosynthesis | 5.65E-05 | 3.90E-03 | 2.52E-05 | 4.53E-05 | 2.20E-16 | 6.60E-16 |
| PWY0-1296: purine ribonucleosides degradation | 6.45E-05 | 3.90E-03 | 5.07E-10 | 2.28E-09 | 1.58E-09 | 3.56E-09 |
| PWY-4981: L-proline biosynthesis II (from arginine) | 4.06E-04 | 1.96E-02 | 2.20E-16 | 1.98E-15 | 2.36E-02 | 2.71E-02 |
| PWY-5177: glutaryl-CoA degradation | 8.99E-04 | 3.25E-02 | 2.46E-03 | 2.77E-03 | 1.65E-02 | 2.47E-02 |
| PWY-6507: 4-deoxy-L-threo-hex-4-enopyranuronate degradation | 7.90E-04 | 3.25E-02 | 8.27E-05 | 1.24E-04 | 4.50E-02 | 4.50E-02 |
| PWY-5188: tetrapyrrole biosynthesis I (from glutamate) | 1.27E-03 | 4.09E-02 | 1.27E-04 | 1.64E-04 | 2.20E-16 | 6.60E-16 |
